# Identification of Purple Acid Phosphatases in Chickpea and Potential Roles of *CaPAP7* in Seed Phytate Accumulation

**DOI:** 10.1038/s41598-017-11490-9

**Published:** 2017-09-08

**Authors:** Jyoti Bhadouria, Ajit Pal Singh, Poonam Mehra, Lokesh Verma, Rishi Srivastawa, Swarup K. Parida, Jitender Giri

**Affiliations:** National Institute of Plant Genome Research, Aruna Asaf Ali Marg, New Delhi, 110067 India

## Abstract

Purple acid phosphatases (PAPs) play important roles in phosphate (Pi) acquisition and utilization. These PAPs hydrolyze organic Phosphorus (P) containing compounds in rhizosphere as well as inside the plant cell. However, roles of PAPs in one of the most widely cultivated legumes, chickpea (*Cicer arietnum L*.), have not been unraveled so far. In the present study, we identified 25 putative PAPs in chickpea (CaPAPs) which possess functional PAP motifs and domains. Differential regulation of *CaPAP*s under different nutrient deficiencies revealed their roles under multiple nutrient stresses including Pi deficiency. Interestingly, most of the *CaPAP*s were prominently expressed in flowers and young pods indicating their roles in flower and seed development. Association mapping of SNPs underlying *CaPAP*s with seed traits revealed significant association of low Pi inducible *CaPAP*7 with seed weight and phytate content. Biochemical characterization of recombinant CaPAP7 established it to be a functional acid phosphatase with highest activity on most abundant organic-P substrate, phytate. Exogenous application of recombinant CaPAP7 enhanced biomass and Pi content of Arabidopsis seedlings supplemented with phytate as sole P source. Taken together, our results uncover the PAPs in chickpea and potential roles of CaPAP7 in seed phytate accumulation.

## Introduction

Phosphorus (P) is essential for plant growth; however, its bio-availability is limited as the available inorganic phosphate (Pi) constitutes only 20–50% of total soil P and the rest remains fixed in the form of organic and inorganic complexes. Pi taken up by roots is subsequently translocated to vegetative and reproductive tissues including developing seeds. Mature plants store the majority of this Pi in the form of highly complex phytic acid and related salts (phytate) in seeds. Processes like seed germination require large amount of Pi where phytate is hydrolysed to release Pi. Few Acid Phosphatases (APases) like Purple Acid Phosphatases (PAPs) are involved in the hydrolysis of phytate complexes to release Pi for plant use in cell or rhizosphere^1, 2^. Secretory PAPs hydrolyse P in soil while non-secretory ones release Pi from cellular organic-P complexes.

PAPs belong to the metallophosphoesterases superfamily, members of which are cosmopolitan in animals, plants, bacteria and fungi^3, 4^. The active form of these enzymes has a binuclear metal center which consists of Fe(III)-Fe(II) in animals and either Fe(III)-Zn(II) or Fe(III)-Mn(II) in plants^5^. These chromophoric metal ions are critical for the functioning of PAPs as their replacement leads to the loss of enzymatic activity^6^. The five conserved motifs contain seven metal ligating residues (**D**xG–G**D**XX**Y**–G**N**H(D/E)–VXX**H**–G**H**X**H**; bold letters indicate metal ligating residues) which form a bimetallic center and are responsible for their activity^7^. While most of the PAPs are active on wide range of substrate, some PAPs (NtPAP, AtPAP15, AtPAP23, GmPhy and OsPHY1) are reported to exhibit higher activity on phytate^8–12^. Nonspecific PAPs exhibit high activity against different phoshomonoesters which include ATP, phosphoenolpyruvate and phosphoproteins^13–15^. Functionally, PAPs play key roles in Pi acquisition and utilization. One common feature of PAPs is their inducibility by low Pi in plants. Many of them also conferred low Pi tolerance on overexpression^11, 13–25^. However, their other roles also include ROS metabolism, nitrogen fixation, cellulose and carbon metabolism^22, 26, 27^. Since, the functions of all known PAPs have not been elucidated, it is difficult to assign specific role for many PAPs. Further, PAPs often exist as multigene family members in plants^7, 23, 28^.

Chickpea is the world’s third-most important cultivated legume crop. Around 6.2 mha of arable land in India is under chickpea cultivation. However, majority of chickpea growing areas are either marginal or sub-marginal lands with sub-optimal Pi levels. Thus, Pi deficiency is a critical constraint for chickpea production in India^29^. Amidst this scenario, organic-P mobilizing PAPs can be an effective resource to improve Pi utilization efficiency of chickpea. However, information on  chickpea PAPs (*CaPAP*s) is missing so far. Therefore, in the present study, we first identified 25 novel *CaPAP*s and investigated their potential roles in Pi homeostasis. Many of the *CaPAP*s were found to be differentially regulated by Pi deficiency. However, unlike some of the past studies, *CaPAP*s did not show specific induction to Pi deficiency. This intrigued us to explore broader roles of CaPAPs in chickpea. Interestingly, majority of the *CaPAP*s were highly expressed in reproductive tissues. Further, association studies revealed strong association of low Pi inducible *CaPAP7* with seed weight and phytate content. Therefore, in the present study, we investigated *CaPAP7* for its possible roles in low Pi tolerance and regulation of seed weight/phytate content.

## Methods

### Identification of PAPs in chickpea

The PAP sequences from 17 different organisms (Table S1) were identified from sequence databases like TAIR, TIGR, Plant GDB, Uniprot; using phrase “purple acid phosphatase”. The organisms selected were *Arabidopsis thaliana*, *Oryza sativa*, *Solanum lycospersicum*, *Medicago trunculata*, *Hordeum vulgare*, *Physcomitrella patens*, *Vitis vinifera*, *Caenorhabditis elegans*, *Homo sapiens*, *Dictyostelum discordeum*, *Vigna radiata*, *Rhizobium galegae*, *Phaseolus vulgaris*, *Glycine max*, *Zea mays*, *Solanum tuberosum* and *Chlamydomonas reinharbtii*. The sequences so obtained were searched for the presence of metallophos domain using SMART tool (http://smart.embl-heidelberg.de/). Total sequences retrieved with metallophos domain were 348 (Text S1). The sequences with metallophos domain were selected, aligned and used to generate a Hidden Markov Model (HMM). The HMM profile was used to identify PAPs in chickpea using HMMER search in chickpea (Kabuli) protein database (http://ceg.icrisat.org/gt-bt/ICGGC/GenomeManuscript.htm). Sequences so obtained were then screened for the presence of metallophos domain and five conserved motifs (**D**XG/G**D**XXY/G**N**H(D/E)/VXX**H**/G**H**X**H**), and finally 25 putative CaPAPs were identified. These PAPs were aligned with Arabidopsis PAPs (AtPAPs) using Clustal X2 and annotated in accordance to their homology with corresponding AtPAPs.

###  Analysis of *CaPAP*s sequence

For comprehensive nucleotide level structural annotation of CaPAPs, like chromosomal/pseudomolecule location, genomic DNA sequence and coding sequence (CDS) were obtained from kabuli database (http://ceg.icrisat.org/gt-bt/ICGGC/GenomeManuscript.htm). The number of exons and introns were identified by aligning cDNA and genomic DNA sequences in GSDS server (http://gsds.cbi.pku.edu.cn/). Number of amino acid, molecular weight and pI of the sequences were predicted using bioinformatics tool, Compute pI (http://web.expasy.org/compute_pi/). To analyse the homology between rice, chickpea, soybean and Arabidopsis PAPs, the amino acid sequences were aligned using Clustal X2 and an unrooted phylogenetic tree was generated using neighbour joining method with bootstrap value 1000. The phylogenetic trees were visualised in MEGA6. For *cis*-element analysis, 3 kb upstream region was selected and P1BS elements (PHR1 binding sites) (GNATATNC) were searched manually using Gene runner (http://www.generunner.net/). The presence or absence of signal peptide was predicted using Signal P4.1 (http://www.cbs.dtu.dk/services/SignalP/). Subcellular localization was predicted using CELLO v2.5 (http://cello.life.nctu.edu.tw/). Glycosylation sites were predicted using GlycoEP (http://www.imtech.res.in/cgibin/glycoep/glyechk). Peroxisome Targeted Signal (PTS) was predicted using PredPlantPTS1 as described previously^30^.

### Expression analysis of *CaPAPs* in different plant tissues

We utilized the transcriptome data from CTDB (chickpea transcriptome database) (http://www.nipgr.res.in/ctdb.html) generated by Garg *et al*.^31^ in order to identify the expression pattern of *CaPAP*s in different tissues (root, shoot, flower bud and young pod). Heat map was generated on RPM (number of unique reads mapped to each transcript per million) values using MeV 4.6.0 tool (http://mev.tm4.org/#/welcome).

### Trait association analysis of *CaPAPs*

For association mapping, seed weight (100 seed weight in gm) and phytic acid content (in mg/100gm) data were retrieved from Kujur *et al*.^32^ and Joshi-Shah and Reddy^33^, respectively. *CaPAP*s based SNP genotyping information (MAF 5%) as well as kinship matrix (K), PCA (P) data and population structure ancestry coefficient (Q matrix) generated from 92 chickpea accessions (association panel) were analysed using TASSEL v5.0, as described^32^. The potential SNP loci in the diverse coding and non-coding sequence components of *CaPAP*s revealing significant association with seed weight and phytic acid traits at a highest R^2^ (degree of SNP marker-trait association) and lowest FDR adjusted P-values (threshold P ≥ 10^−6^) were selected.

### Plant growth condition and nutrient deficiency treatments

Chickpea seeds of accession ICC 4958 were used in all the experiments. For generating nutrient deficiency conditions, seedlings were raised in Hoagland media according to the conditions and protocol as described^34^. Briefly, plant growth conditions were maintained at 12/12 h photoperiod, 23/18 °C, 200-300 µM photons/m^2^/s photon density and ~70% relative humidity. Tissues were harvested after 7 and 15 days of treatment and unless stated otherwise experiments were performed in three biological replicates.

### qRT-PCR based analysis of gene expression

Gene expression analysis was done as described, previously^34^. Briefly, root samples were taken at 7 days and 15 days after treatment for each nutrient deficiency. Tissues were frozen in liquid nitrogen and stored at −80 °C. The TRIzol method was used to extract total RNA from root. Prior to cDNA synthesis, DNaseI treatment was given to get rid of genomic DNA contamination from RNA. cDNA was synthesised using High Capacity cDNA Reverse Transcription Kit (Applied Biosystems) according to manufacturer’s instructions.

The primers for quantitative real time PCR (qRT-PCR) were designed using PRIMER EXPRESS version 2.0 (PE Applied Biosystems^TM^, USA) with default parameters from coding region of the genes (Table S2). Each primer pair was analysed for its specificity using NCBI BLAST. The reaction was performed in Applied Biosystems 7500 Fast Real Time PCR. The relative expression was calculated using the ^ΔΔCt^ method. The elongation factor 1-α (AJ004960) was used as endogenous control and three biological replicates were considered for the experiment. To test the level of significance, Student’s *t*-test was applied.

### Cloning and subcellular localization of CaPAP7

The coding region of *CaPAP7* was cloned in pSITE3CA vector to express it as N-terminal YFP fusion protein (YFP:CaPAP7). Plasmid carrying this construct was then transformed into onion epidermal cells as described^25^. Fluorescence was visualised under confocal microscope, AOBS TCS-SP2 (Leica, Germany).

### Biochemical characterization of recombinant CaPAP7

For biochemical characterization, ORF sequence of *CaPAP7* was amplified using gene-specific primers (Table S2). The PCR product of 1008 bp was cloned into pET28a vector and confirmed by sequencing. BL21 (DE3) pLysS cells were used for protein induction. The recombinant protein was induced with 0.3 mM IPTG at 16 °C for 12-16 h. The induced protein was purified using affinity chromatography (Ni-NTA). To confirm the identity of induced protein, coomassie-stained protein bands were digested with trypsin as per manufacturer’s protocol and then identified by 4000Q TRAP LC/MS/MS.

The phosphatase activity assays were performed to study optimum temperature, pH, cofactor and substrate for CaPAP7 as described^25^. The optimum temperature for the PAP activity was measured over a temperature range of 25 °C to 60 °C in 50 mM sodium acetate buffer (pH 4.9), MgCl_2_ (5 mM) using 10 mM *para*-nitrophenylphosphate (pNPP), as substrate for 30 min. To identify the optimum pH for CaPAP7 activity, a pH range of 4.0 to 8.0 was set using different buffers with varying pH and pNPP (10 mM) as substrate at 37 °C for 30 min. In order to determine the substrate specificity, CaPAP7 activity was assayed on different substrate namely, ATP, ADP, AMP, PPi, P-serine, P-threonine, glucose-6-P, fructose-6-P, pNPP, 2-deoxyriboseP and phytic acid at 37 °C for 30 min. In order to identify cofactors and inhibitors of CaPAP7, activity assays were performed with 5 mM of chloride salts of different metal ions and sodium salts of different cations as described earlier^25^.

### Assessment of *CaPAP7* phytase activity using Arabidopsis seedlings

In order to assess the impact of recombinant CaPAP7 (phytase activity) on plant growth, Arabidopsis seeds were germinated on ½MS media in plates vertically. Four-days-old seedlings with uniform root growth were transferred to ½ MS plates containing 1 µM KH_2_PO_4_ (−Pi), 625 µM KH_2_PO_4_ (+Pi), 5 µM phytate (Phytate) and 5 µM phytate + 150 ng/ml enzyme (Phytate + E). CaPAP7 protein in Tris-Cl buffer was spread on 5 µM phytate plates before seedlings transfer. All the growth parameters (root length, biomass, and lateral root length) were analysed after 7 days of transfer. Soluble Pi estimation was done as described^35^. Root surface associated APase activity was visualized using 0.015% BCIP (5-bromo-4-chloro-3- indolyl-phosphate) as described^25^.

### Gene expression profile *of CaPAP7* in chickpea genotypes with contrasting seed weight

Four contrasting accessions for seed weight and phytate content were selected based on the information from previous reports^32, 33^. In order to reveal the role of CaPAP7 in phytate remobilisation, we analysed the expression of *CaPAP7* in young pod of these contrasting accessions. Tissues for young pod were collected in three replicates pooled from multiple plants, frozen in liquid nitrogen and thereafter stored at −80 °C. RNA was isolated using TRIzol and cDNA was prepared as described above. The qRT-PCR was done and relative expression was calculated using 2^−ΔCt^ method.

## Results

### PAPs form multigene family in chickpea

**Figure 1:**
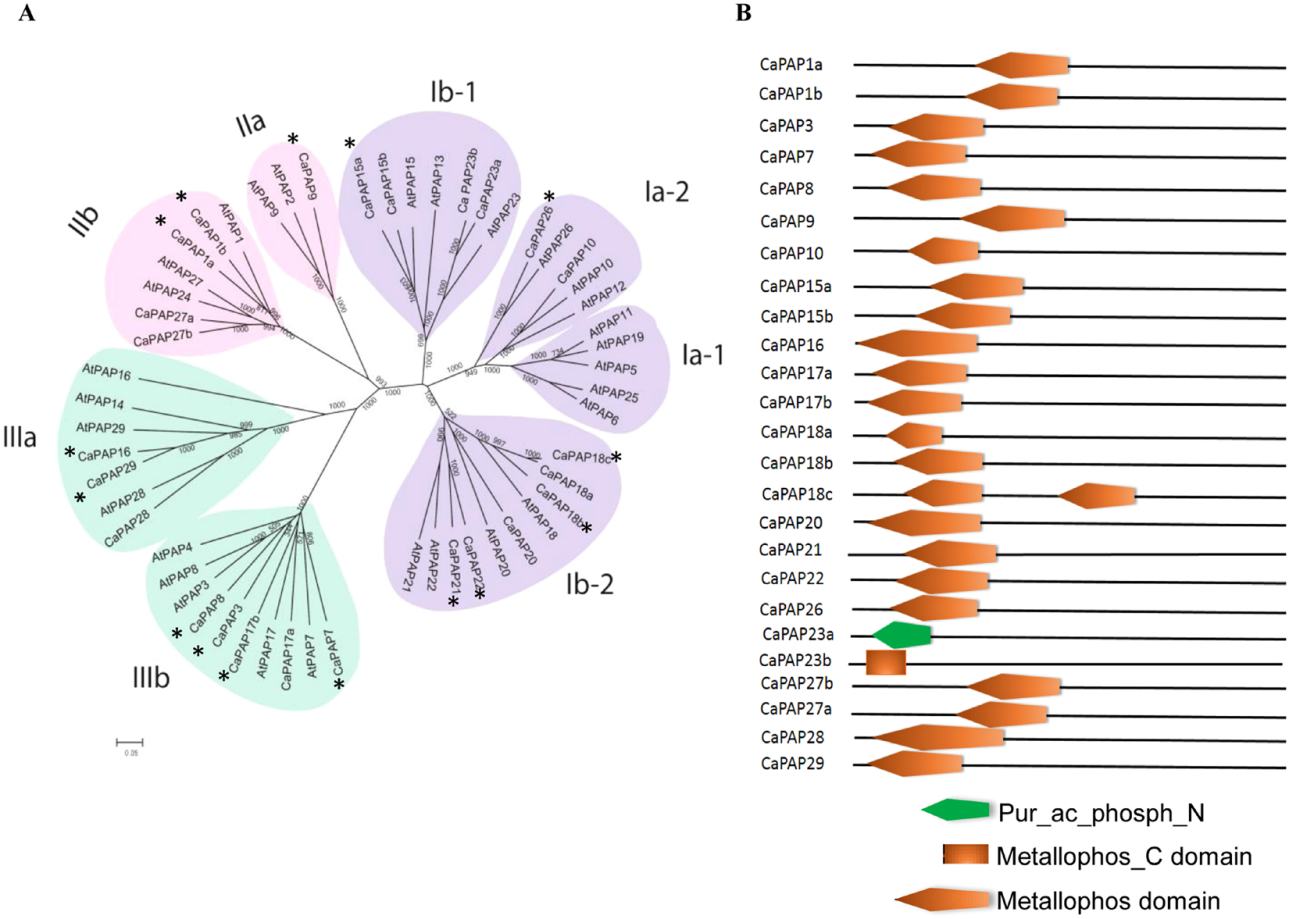
Phylogenetic relationship and domain architecture of CaPAPs. (**A**) Phylogenetic relationship of chickpea and Arabidopsis PAPs. The amino acid sequences of CaPAPs and AtPAPs were aligned using Clustal X2 and the phylogenetic tree was constructed using NJ method with bootstrap value 1000. Bootstrap value mentioned at each node. “*” indicates CaPAPs with signal peptide (**B**) Conserved domains present in CaPAPs. The amino acid sequence of CaPAPs were analysed in SMART (http://smart.embl-heidelberg.de/) tool. Pur_ac_phosph_N; N terminal domain of purple acid phosphatase, Metallophos_C domain; C terminal domain of metallophos domain.

Using sequence homology approach and conserved domain analysis, we identified 25 putative PAPs in chickpea genome which were annotated according to their homology to Arabidopsis PAPs (Fig. 1A). Of these, complete metallophos domain was present in twenty-three while two (CaPAP23a and CaPAP23b) have partial domain. CaPAP23a had only pur_ac_phosphN terminal domain and CaPAP23b had only metallophos C domain which is essential for the phosphatase activity (Fig. 1B). The sequence analysis revealed that 21 CaPAPs have all the five blocks of conserved motifs (**D**XG/G**D**XX**Y**/G**N**H(D/E)/VXX**H**/G**H**X**H**) and seven metal ligating residues which are characteristics of known PAPs. In CaPAP16, 28 and 29 “**Y**” residue is replaced by “**F**” in the second block (G**D**XX**Y**). Among the remaining PAPs, CaPAP16, 23b lack the fourth block (VXX**H**), CaPAP18a lacks fifth block (G**H**X**H)**, whereas CaPAP23a has two blocks missing; third (G**N**H(D/E) and fifth (G**H**X**H**) (Table S3 **)**. But both the sequences showed significant homology with known PAPs. So we considered them as putative PAPs.

Based on phylogenetic relationship between chickpea and Arabidopsis PAPs, CaPAPs were classified into three major (I, II and III) groups as per earlier classification^7^ **(**Fig. 1A **)**. The twenty- five CaPAPs were divided into seven subgroups (Ia-2, Ib-1, Ib-2, IIa, IIb, IIIa and IIIb) except subgroup Ia-1 where none of the CaPAPs was grouped. Further, phylogenetic analysis of CaPAPs with soybean, Arabidopsis and rice PAPs together suggested that CaPAPs are closer to dicots PAPs as only few of them were grouped with rice PAPs (Fig. S1).

Further, *CaPAP*s were found to be localised to different chromosomes, whereas five genes (*CaPAP15a*, *17a*, *17b*, *18c* and *22*) were present on scaffolds (scaffold 128, 88, 40, 6367 and 1710, respectively) (Table S4). Analysis of gene structure of *CaPAPs* showed that number of exons varies from 2 (*CaPAP9*) to 12 (*CaPAP1a*, *1b*, *27a* and *27b*) (Table S4). Molecular mass of identified PAPs varies from 17.33 kDa (CaPAP23a) to 73.91 kDa (CaPAP9). On prediction of secretory or non-secretory PAPs, we found the presence of signal peptide in 15 CaPAPs namely, CaPAP1a, 1b, 3, 7, 8, 9, 15a, 16, 18b, 18c, 21, 22, 24, 26 and 29, whereas no signal peptide was identified in remaining 10 PAPs i.e. CaPAP10, 15b, 17a, 18a, 20, 23a, 23b, 27a, 27b and 28. In order to correlate the nature of signal peptide, we predicted the subcellular localization of CaPAPs with the help of CELLO tool (http://cello.life.nctu.edu.tw/). It turned out that most of the CaPAPs i.e. 22 out of 25 were either secretory or localised to lysosomes. CaPAP7 and 16 were predicted to be localised to cytoplasm while CaPAP28 to plasma membrane. Interestingly, CaPAP18b was predicted to be localised to nucleus and also has potential secretory nature **(**Table S5 **)**. Since, some of the PAPs have been reported to be localised in peroxisomes^30^, we determined presence of PTS1 (Peroxisome Targeted Signal) in CaPAPs. Interestingly, only low P responsive CaPAP17a showed presence of PTS in its C terminal region. Furthermore, glycosylation sites were present in all the CaPAPs varying in numbers 1 to 8 (Table S5).

As PAPs are largely reported as Phosphate Starvation Response (PSR) genes, we analysed *CaPAPs* promoters (3 kb upstream) for any putative *cis*-acting elements which can act as binding sites for transcription factors involved in Pi deficiency response. *P1BS* (PHR1 binding sites) elements are considered to be highly Pi responsive as AtPHR1 (master regulator of P deficiency response) binds to this region and regulate its downstream targets. Analysis of promoter regions of all chickpea PAPs for the presence of *P1BS* elements revealed that *P1BS* elements were present in the promoter region of 18 CaPAPs including *CaPAP1a*, *1b*, *3*, *8*, *9*, *10*, *15a*, *15b*, *17a*, *17b*, *18c*, *21*, *22*, *23a*, *23b*, *27b*, *28* and *29* while we did not find *P1BS* elements in seven CaPAPs including *CaPAP7*, 16, *18a*, *18b*, *20*, *26* and *27a* **(**Table S5 **)**.

### *CaPAP*s are differentially expressed under Pi deficiency

**Figure 2:**
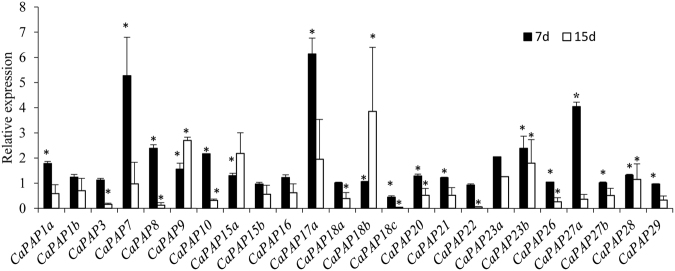
Relative expression profile of *CaPAPs* under Pi deficiency after 7d (early response) and 15d (late response) of treatments. qRT-PCR was used for quantification of gene expression. The relative gene expression in stressed plants was calculated considering untreated plants as control. *EF1α* was used as endogenous control. Error bars represent SE of average of three replicates (n = 3). *p < 0.05.

Since most of *CaPAPs* have *P1BS* elements in their promoter region, therefore, we studied their expression behaviour under Pi deficiency. Most of the *CaPAPs* (12 out of 25 genes which include *CaPAP1a*, *1b*, *7*, *8*, *10*, *15a*, *17a*, *20*, *21*, *23b*, *27a* and *28*) showed significant upregulation except *CaPAP1*8*c* which was downregulated after 7 days of deficiency (Fig. 2). *CaPAP17b* did not show detectable expression. The expression of ten genes (*CaPAP3*, *8*, *10*, *18a*, *18c*, *20*, *22*, *26*, *27a* and *29*) found to be downregulated after 15 days of deficiency except two genes, *CaPAP9* and *CaPAP1*8*b* which were found upregulated. This indicated that most of the chickpea *PAP* genes are early Pi deficiency inducible, while *CaPAP9b* and *CaPAP18b* were late inducible to the Pi deficiency (Fig. 2). Moreover, four genes including *CaPAP1b*, *8*, *15* and *27a* were found exclusively upregulated under Pi deficiency showing their Pi specific behaviour (Figs S2 and S3). In addition to low Pi; *CaPAPs* also showed differential expression under low N, K, Fe and Zn indicating their roles in these deficiencies (Figs S2 and S3).

### *CaPAPs* showed preferential high expression in reproductive tissues

**Figure 3:**
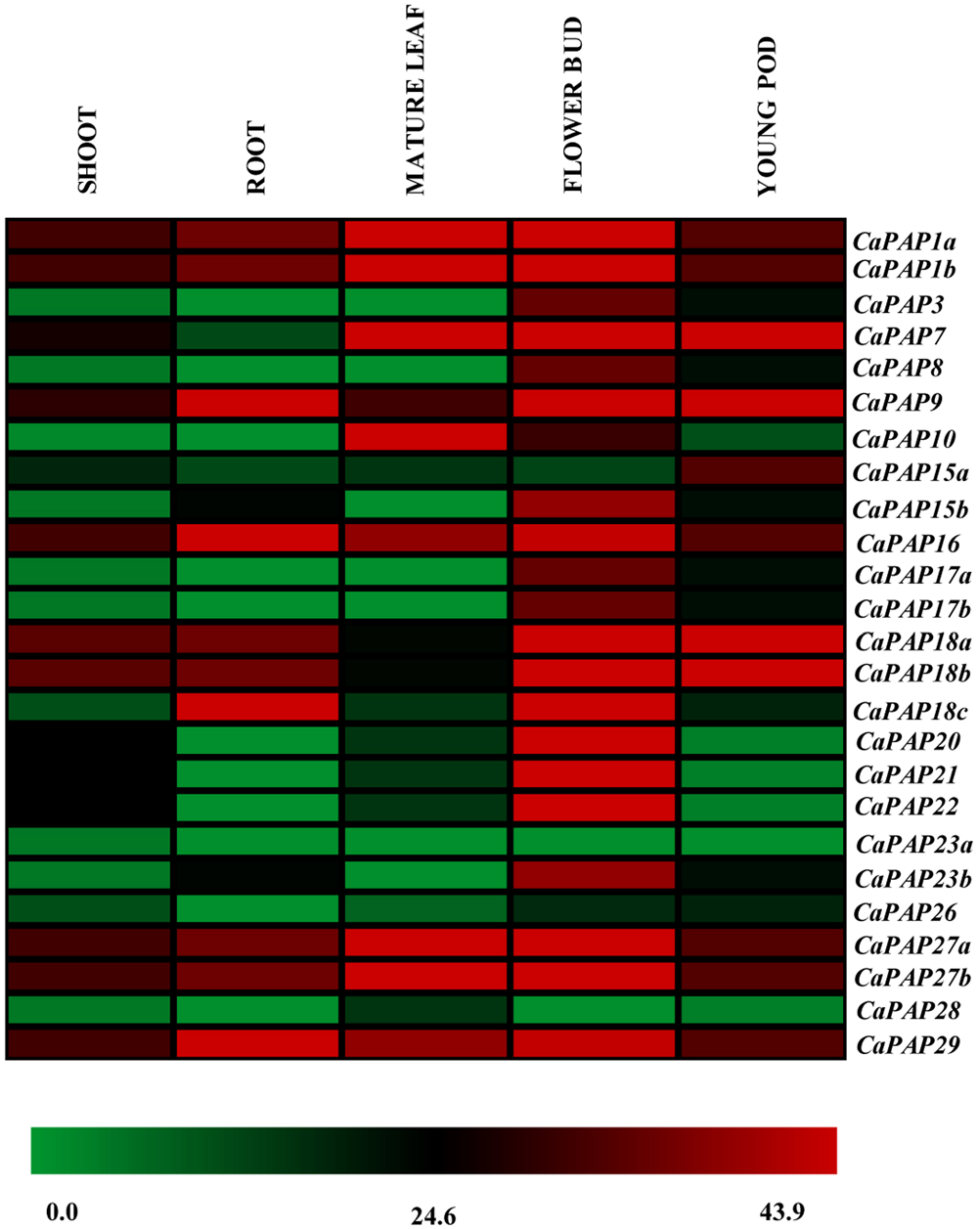
Gene expression profile of *CaPAPs* in shoot, root, mature leaf, flower bud and young pod of chickpea. Heat map was generated with RPM (Reads Per Million) values retrieved from CTDB (http://www.nipgr.res.in/ctdb.html). Scale bar represents RPM values.

Spatial expression of *CaPAPs* in different tissues (root, shoot, mature leaf, flower bud and young pod) retrieved from CTDB database showed that most of the *PAPs* express in all the tissues but have exceptionally high expression in flower bud and young pod **(**Fig. 3). Further, except four *CaPAPs*, other root expressing ones did not express as high in roots as they did in reproductive tissues. Among all the tissues, *CaPAP*s showed least expression in shoot. This strong expression in flower bud and young pod indicates their important functions during reproductive development.

### *CaPAP*s showed strong association with seed phytate content and seed weight in diverse chickpea accessions

**Figure 4:**
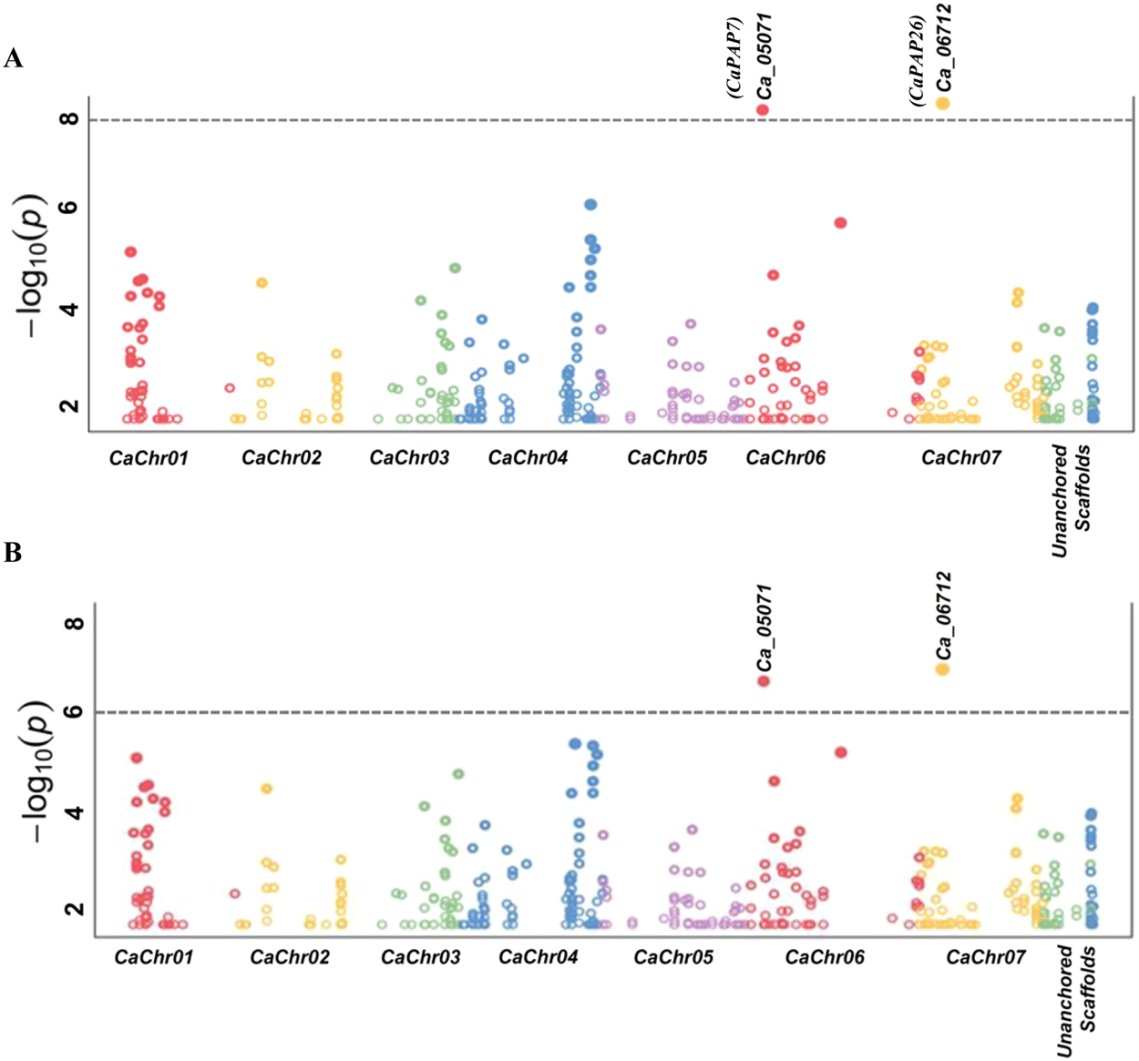
Association of *CaPAP7* and *CaPAP26* with seed weight (**A**) and seed phytate content (**B**). The x-axis indicates the relative density of PAP gene based SNPs physically mapped on eight chromosomes and unannotated scaffold of Kabuli genome. The y-axis represents the −log_10_ p-value for significant association with traits. The SNPs with p-value ≤ 1 × 10^−8^ for seed weight (**A**) and 1 × 10^−6^ for seed phytate content (**B**) showing strong association are demarcated with dotted line.

Since most of the *CaPAPs* showed high expression in reproductive tissues (flowering bud and young pod), we performed candidate gene based association mapping in diverse chickpea accessions for phytate content and seed weight. For this, genotyping data of SNPs from diverse coding and non-coding sequence components of *CaPAPs* along with phenotypic data (phytic acid and seed weight) of 92 accessions were collected. SNPs underlying *CaPAPs* were retrieved from 92 chickpea accessions and associated with seed weight data as described, previously^32^. Interestingly, we found strong association of a SNP (G/A) of Ca_05071 (*CaPAP7*) with seed weight (P value 1.8 × 10^−9^) and seed phytate content (P value 1.4 × 10^−6^). The proportion of phenotypic variation explained (PVE) for seed weight and phytate content by this SNP was found to be 25 and 19%, respectively (Table 1, Fig. 4). Additionally, another DRR (down-stream regulatory region) SNP, (A/C) in Ca_06712 (*CaPAP26*) was associated with seed weight trait (28% PVE with 2.4 × 10^−10^) and phytate content (21% PVE with P value 1.8 × 10^−7^). Closer analysis revealed that allele ‘G’ in URR of *CaPAP7* mapped on chromosome 6 was associated with high seed weight and phytate, whereas allele ‘A’ is associated with low seed weight and low phytate content (Table 1). Similarly, allele ‘A’ in DRR of *CaPAP26* mapped to chromosome 7 was associated with high seed weight and high phytate, whereas allele ‘C’ is associated with low seed weight and low phytate content.

**Table 1:** PAP gene-derived SNP loci regulating seed weight and phytate content identified by association mapping.

Chromosomes	SNP physical positions (bp)	SNPs	Gene accession IDs	Known/putative functions	Association analysis		Traits associated
					P	PVE (%)	
*Ca_Kabuli_Chr6*	12978236	[G/A]	Ca_05071	CaPAP7	1.8 × 10^−9^	25	100-seed weight
*Ca_Kabuli_Chr6*	12978236	[G/A]	Ca_05071	CaPAP7	1.4 × 10^−6^	19	Phytate content
*Ca_Kabuli_Chr7*	6395883	[A/C]	Ca_06712	CaPAP26	2.4 × 10^−10^	28	100-seed weight
*Ca_Kabuli_Chr7*	6395883	[A/C]	Ca_06712	CaPAP26	1.8 × 10^−7^	21	Phytate content

### *CaPAP7* is differentially regulated in contrasting genotypes for seed weight and phytate content

**Figure 5:**
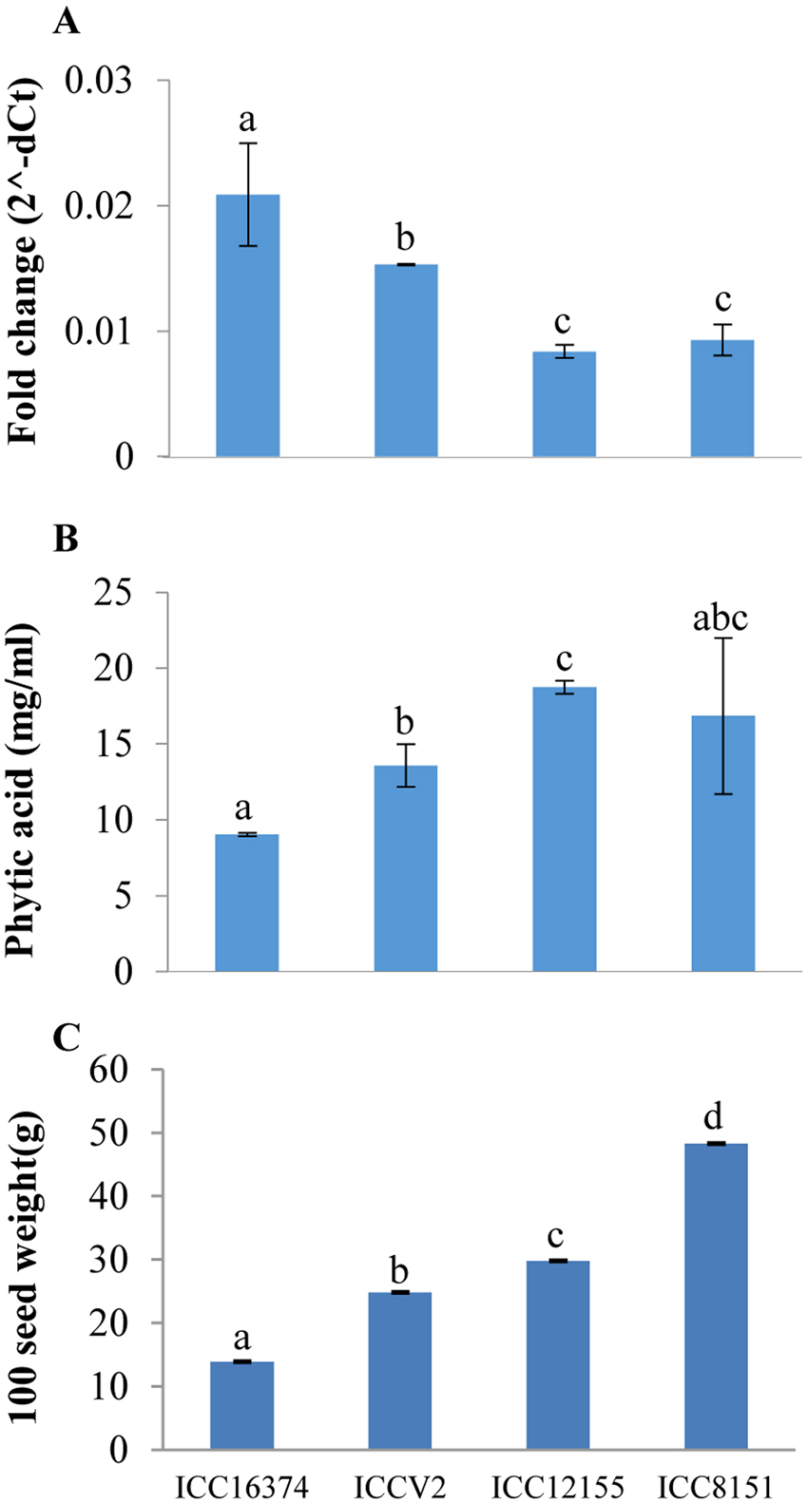
Expression profile of *CaPAP7* in genotypes differing for seed weight and phytate content. (**A**) Expression pattern of *CaPAP7* in young pod in contrasting chickpea accessions. Fold change was calculated using EF1-α as reference gene. Three biological replicates were considered for expression analysis. Each biological replicate comprise of pooled sample from multiple plants. Error bars indicate SE (n = 3). (**B**) Phytic acid content of contrasting chickpea accessions (Joshi-Saha *et al*.^33^). Error bars represent SE (n = 2) (**C**) Average seed weight (100 seed weight in g) of contrasting chickpea accessions (Kujur *et al*.^32^). Error bars represent SE (n = 3) *CaPAP7* expression was in negative correlation with the seed weight (r = −0.82) and seed phytate content (r = −0.99). Different letters at the top of bar indicates different significant classes determined by one-way ANOVA followed by Duncan’s multiple range test.

Due to its induction in response to low Pi, conservation of all key residues and strong association with seed weight and seed phytate content traits, *CaPAP7* was selected for further characterization. In order to validate association of URR SNP of *CaPAP7* with seed weight and phytate content, we analysed the expression pattern of *CaPAP7* in four accessions of chickpea varying in seed weight and phytate content (ICC16374, ICCV2, ICC12155 and ICC8151). Further, seed weight and phytate content were found strongly correlated with each other. Therefore, we analysed the expression pattern of *CaPAP7* in young pod where phytate synthesis/accumulation is expected to be high. *CaPAP7* showed higher expression in accessions with low seed weight and phytate content. While its expression was lower in accessions with higher seed weight and phytate content (Fig. 5). Therefore, a negative correlation of *CaPAP7* expression was obtained with the seed weight (r = −0.82) and seed phytate content (r = −0.99). This data further strengthen the important role of CaPAP7 in chickpea seed phosphate/phytate accumulation.

### *CaPAP7* is localized in cytoplasm and encodes a functional acid phosphatase

*In-silico* tools predicted CaPAP7 to be localised in the cytoplasm. To confirm this *in vivo*, we cloned *CaPAP7* CDS in pSITE3CA vector to produce an YFP fused protein and analysed its localisation in onion epidermal cells. YFP fluorescence analysis confirmed its cytoplasmic localisation as predicted **(**Fig. S4 **)**.

**Figure 6:**
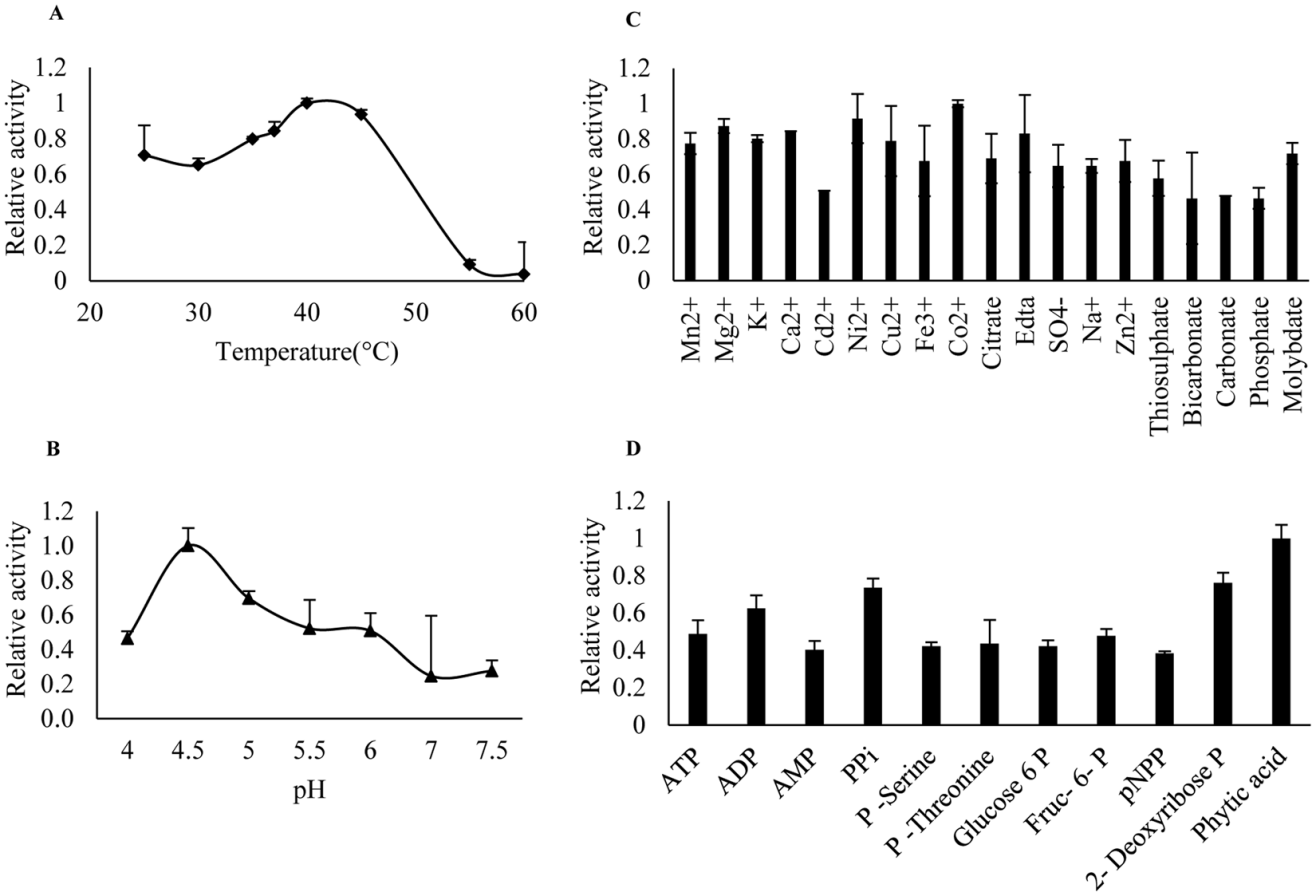
Biochemical properties of recombinant CaPAP7. (**A**) The temperature profile of CaPAP7 on pNPP in sodium acetate buffer at various temperatures for 30 min. (**B**) pH profile. The CaPAP7 activity was assayed in various buffers (50 mM) with different pH at 37 °C for 30 min. (**C**) Effect of different cofactors on CaPAP7 activity. (**D**) CaPAP7 activity on different substrates (10 mM) at 37 °C for 30 min. Values represent average of two replicates with std. error. Experiment was repeated three times with similar results (n = 2).

We next studied, whether CaPAP7 is a functional enzyme to further contemplate on its functions. Recombinant protein was produced in bacteria and purified. A band of expected size ~38 kDa was detected on SDS-page (Fig. S5). The identity of induced recombinant CaPAP7 was confirmed with LC-MS/MS (MASCOT score 120) **(**Fig. S6 **)**. CaPAP7 showed activity in the temperature range of 25–45 °C and pH range of 4–6 (Fig. 6). Its high phosphatase activity in acidic pH confirmed it as APase. Further, activity assays with different divalent cations and anions revealed no specific cofactor preference of CaPAP7. However, high phosphate and carbonate concentration seemed to be slightly inhibitory for phosphatase activity of CaPAP7 (Fig. 6C). The enzyme activity assay showed that CaPAP7 has a broad substrate specificity hydrolysing different Pi containing compounds namely, ATP, ADP, AMP, PPi, P-serine, P-threonine, glucose-6-P, fructose-6-P, pNPP, 2-deoxyribose-P and phytic acid. Intriguingly, it exhibited maximum catalytic activity for phytic acid **(**Fig. 6D **)**. The specific activity of recombinant CaPAP7 with pNPP and phytate was 0.3825 and 0.620 nmolug^−1^ min^−1^, respectively.

### Exogenous application of recombinant CaPAP7 protein improved Arabidopsis growth on phytate containing media

**Figure 7:**
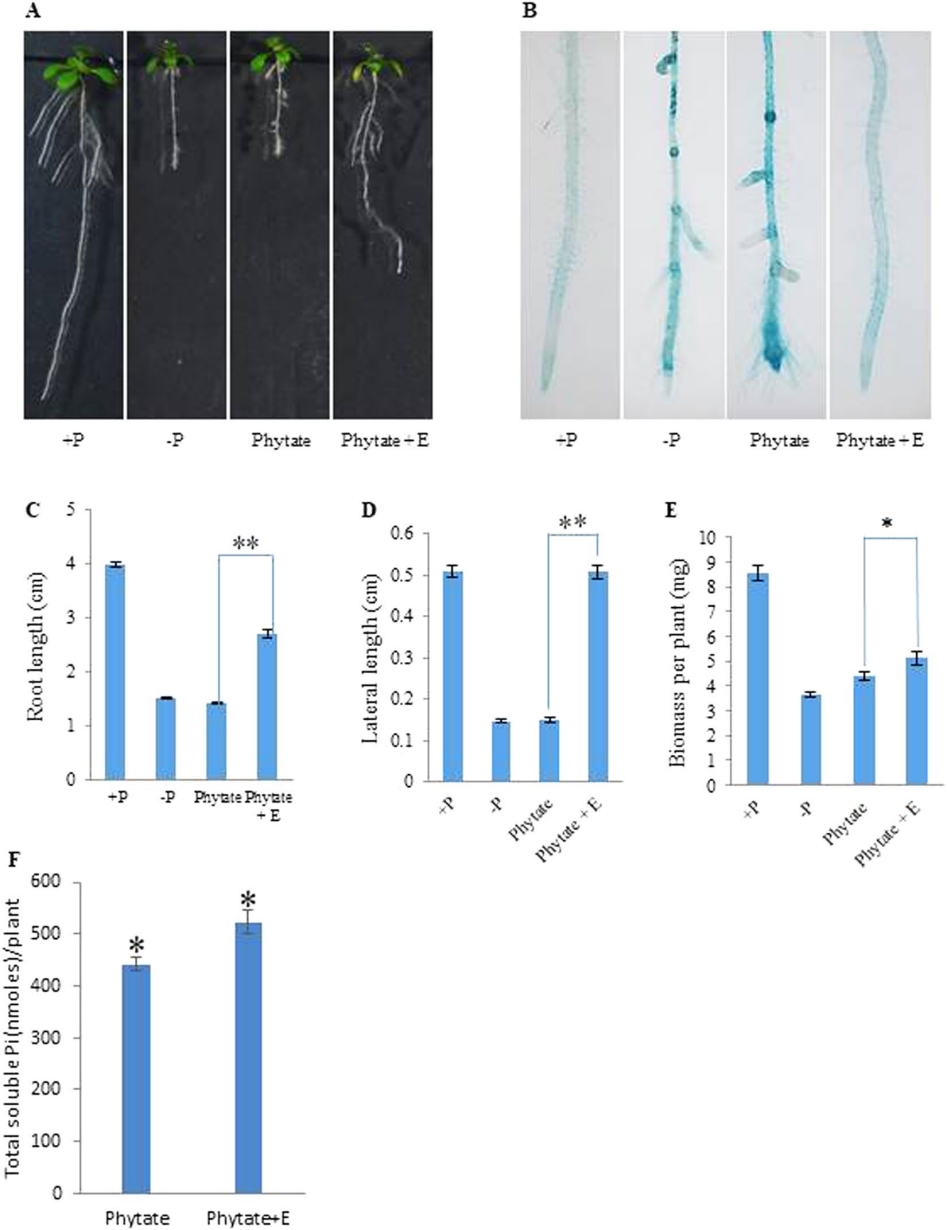
Effect of purified recombinant CaPAP7 on phytate hydrolysis and Arabidopsis growth parameters. (**A**) Phenotypic deference in P supplemented ( + P), P deficient (−P), P deficient + phytate (phytate) and P deficient + phytate + CaPAP7 (phytate + E) grown Arabidopsis seedlings. (**B**) Visualization of acid phosphatase activity by BCIP overlay on roots of Arabidopsis seedlings. (**C**) Average root length (n = 7 in six replicates). (**D**) Average lateral root length (n = 7 in six replicates). (**E**) Average biomass per plant (n = 7 in six replicates). (**F**) Total soluble Pi (nmoles per plant) (n = 6 in six replicates). All analysis was done after 7 days of growth on above said media. Error bars represent SE. Students t-test was performed for the statistical analysis **p < 0.001, *p < 0.05.

As CaPAP7 has highest activity for phytic acid which is an abundant non-available form of P in soil. So to validate its phytase activity and its impact on hydrolysis of phytate in plant, we grew Arabidopsis seedlings on phytate containing media. As expected, seedlings showed ~50% reduction in root elongation on media without Pi (−P) and phytate (as P source) as compared to +P media (Fig. 7). To further test whether phytate can induce low Pi stress in our set-up, we visualized root APase activity using BCIP and found increased APase activity under −P and phytate supplemented conditions. However, when seedlings were grown on phytate media overlaid with purified CaPAP7 enzyme; root length increased by 50% as compared to seedling on phytate without enzyme. Seedlings supplemented with CaPAP7 also showed increased lateral roots length (~233%) and higher biomass (~11%) than ones without enzyme (Fig. 7). Pi accumulation per plant was also increased (~22%) in seedlings with the application of CaPAP7 in phytate media in comparison to phytate only media. All these results strongly suggest that CaPAP7 was able to hydrolyze phytate to release Pi for better plant growth **(**Fig. 7 **)**. To this end, we conclude that CaPAP7 encodes a functional enzyme which is able to hydrolyze phytate and potentially influences seed phytate accumulation, and thereby seed weight.

## Discussion

Chickpea is one of the most important legume crops cultivated in the semiarid regions of the world. Chickpea being a leguminous crop has higher ability to mobilise the soil P^36^. Along with phosphate, legumes are known to restore other nutrients to the soil such as nitrogen through symbiotic N_2_ fixation which makes them highly important for the sustainable agriculture^37^. Legumes demand more Pi than non nodulating nitrogen-fixing plants as it is critically important for legume- rhizobia symbiosis^38–40^. Therefore, Pi deficiency affects chickpea development in dual manner; limits both growth and nitrogen fixation. One of the adaptive strategies evolved in diverse plants to increase labile Pi pool is secretion of APases^41, 42^. PAPs are APases with broad substrate specificity and are reported to hydrolyse various organic-P compounds in different plants (Arabidopsis, rice, soybean, kidney bean)^4, 8, 11, 12^. Here we identified 25 novel PAPs in chickpea which form three main groups (I, II, III) and seven subgroups (Ia-2, Ib-1, Ib-2, IIa, IIb, IIIa and IIIb) as described for Arabidopsis^7^. This indicates similarity in their function and behaviour; except clade Ia-1 which includes only AtPAPs. CaPAPs exhibited more similarity to AtPAPs and GmPAPs than to OsPAPs, suggesting that their behaviour and function are similar to other dicots. While all CaPAPs had an Arabidopsis homolog, a lower number of PAPs were retrieved in chickpea as compared to other legumes and Arabidopsis^7, 23^. This could be due to either incomplete genome sequence in chickpea or peculiar genetic constitution of chickpea, and raise the possibility of identification of more PAPs in future.

PAPs are known to be induced by Pi deficiency to help plants utilize organic sources of P^13, 17, 25, 43, 44^. *CaPAP*s also had *P1BS cis*-elements in their promoter which is involved in low Pi inducibility. Although *CaPAPs* were induced under Pi deficiency; however, level of induction was not very high. Moreover, low Pi inducible *CaPAP7* didn’t possess any *P1BS* element. Further, a direct correlation wasn’t seen between existence of *P1BS* element and low Pi inducibility of PAPs in other plants^28, 45^ indicating role of other unknown regulators. Notably, many of the low Pi inducible genes possess other low Pi responsive *cis-*elements [such as W box (TTGACY), TC element (TCTCTCT) or NIT 2-like elements (AAATATCT)] instead of *P1BS* ^46^. This indicates that *P1BS* elements are not obligatory for low Pi responsiveness of many such genes. Analysis of low Pi inducible CaPAP7 revealed presence of two TC elements in its promoter which might be involved in its low Pi dependent transcriptional regulation.

Most of the *CaPAP*s were also upregulated by nitrogen deficiency, which can be correlated with the large amount of Pi requirement during nitrogen fixation by the nitrogenase complex. Their differential expression in other nutrient deficiencies hinted towards other roles of *CaPAP*s. This deviation from their well-established roles in Pi deficiency is in quite agreement with other observations. For instance, GmPAP21 is involved in P metabolism in root nodules. AtPAP17 is involved in the metabolism of reactive oxygen species while AtPAP26 plays a role in senescence driven Pi remobilization. Similarly, GmPAP3 participates in ROS response while AtPAP2 plays key roles in carbon metabolism^16, 47–49^. In rice PAPs have also been reported to play role in grain filling^50^. Heat inducible PgPAP18 was also reported to play defensive role against several environmental stresses^51^. Some PAPs such as NtPAP12 are involved in root system modulation by degradation of xyloglucan oligosaccharides and cello-oligosaccharides in the cell walls^52^. These evidences suggest diverse roles of PAPs in plants. On such similar lines CaPAPs may also be playing other roles besides Pi deficiency response.

To contemplate on these roles, we studied expression patterns of *CaPAPs* in different tissues. Interestingly, they showed least expression in root. Majority of them showed high expression in flower buds and young pods suggesting their important roles in reproductive development. Incidentally, several *AtPAPs* are also reported to be expressed during reproductive development^9^. High expression of *PAPs* in Arabidopsis pollens has been correlated with increased hydrolysis of phytate and germination of pollen^11^. Function of these enzymes therefore, could also be intracellular; in remobilizing Pi towards seed as seed development requires more P in comparision to normal cellular processes. Seeds also accumulate more P than other organs^53^ and increasing seed P content has been suggested to improve seedling vigour^54^. Interestingly, among 25 identified *CaPAPs*, two *PAPs*, *CaPAP7* and *CaPAP26* showed strong association with both seed weight and phytate content. Chickpea seeds are high in phytate content, and seed size directly correlates with the phytate content of the seed^33^. It is noteworthy here that CaPAP26 is a close homologue of AtPAP26 which is known to be involved in Pi remobilization and senescence^48^. *CaPAP7* showed high low Pi inducibility, localizes to cytoplasm and is of non-secretory nature. Recently, the Arabidopsis homologue of CaPAP7 (AtPAP7) was found to localise in peroxisomes^30^. However, we did not find any PTS in CaPAP7. Further, particle bombardment assay revealed no co-localisation of CaPAP7 with peroxisome marker in onion epidermal cells (data not shown). Therefore, CaPAP7 may not be a true homolog of AtPAP7. Further studies revealed that CaPAP7 to be enzymatically active at pH range (4–6) with optima at 4.5, validating its function as acid phosphatase. Intriguingly, while CaPAP7 showed broad substrate specificity, highest activity was observed on phytate, concluding CaPAP7 as PAP with phytase activity. PAPs are known to be glycosylated which affect their stability, targeting and enzyme kinetics^2^. Therefore, a high phytase activity of CaPAP7 needs further investigation using plant purified native CaPAP7. Nevertheless, PAPs with phytase activity do exist in plants^8, 11^. Few purple acid phosphatases (PAPs) have also been reported to possess phytase activity in soybean, Arabidopsis, rice and tobacco^8, 9, 11, 12, 19^. Recently, OsHAD1, an APase has also been reported to possess both acid phosphatase and phytase activity^55^. Further, improvement of Arabidopsis growth on phytate, when supplemented with CaPAP7 enzyme validated CaPAP7 efficacy to release Pi from phytate by its hydrolysis. Phytases from different sources have also been earlier reported to enhance the plant growth by increasing Pi acquisition and biomass accumulation^10, 56–58^.

In plants, P used by vegetative tissues is ultimately mobilized to developing seeds, where nearly 75% of it is stored in the form of phytic acid^59^. During germination and seedling growth, this phytic acid is broken down by the phytases for utilization by seedlings^60^. Phytate content of chickpea seed is quite high which again corresponds to the need of these phytases during seed development and seed germination. Also, expression of few *CaPAPs* was quite high in mature leaf tissue, where their role can be correlated with the Pi recycling from old tissues to the new ones. Phytate binds with other minerals like Fe, Mg, Zn and therefore, reduces their bioavailability^61, 62^. High consumption of phytate rich food is considered as one reason for mineral deficiency in humans in developing world^63^. Further, almost 50% of applied P fertilizers are removed from farmers field in the form of phytate in seeds every year^59^. Monogasteric animals cannot digest phytate which is excreted out and led to the eutrophication of water bodies. It is further shown that phytate accumulation is not absolutely necessary for seed functions. Rather, plants store excess of Pi in the form of phytate which seeds use during germination^53^. Therefore, low phytate food crops are a necessity and such crops can be engineered with new emerging resources.

Different approaches have been used for reducing seed phytate content by targeting phytate biosynthesis enzymes^63^, its transporter in tissue-specific manner^64^ or by blocking the translocation of Pi to developing seed^65^. One of the problems coupled with lowering phytate content by reducing its biosynthesis in seed is corresponding increase in Pi which inhibits starch biosynthesis^66^. Consequently, low starch accumulation leads to reduced seed weight and crop yield. However, lowering of seed total P by reducing Pi transporter activity does not lead to any significant yield loss while reducing the seed phytate by 50% in rice^64^. Therefore, while reducing phytate content may affect seed fitness in the wild, it may not be a problem for cultivated crops in fertilized farmers’ fields^63^. Here, *CaPAP7* emerged as potential new genetic/genomic regulator for seed phytate content in chickpea. CaPAP7, besides phytate, can also hydrolyse other organic Pi compounds abundant in cell cytoplasm. It showed high expression in tissues with high phytate content i.e. flower bud (pollens) and young pod, its expression levels correlate negatively with seed phytate and weight. Therefore, it is very likely that CaPAP7 mediated phytate hydrolysis in these tissues results in high soluble Pi content which inhibits starch biosynthesis and ultimately leads to low phytate and seed weight. How precisely CaPAP7 regulates this process, needs further investigations. Nevertheless, our data revealed that *CaPAPs* have functional diversification in chickpea while some of them may be involved in Pi acquisition, *CaPAP7* may play important roles in seed phytate accumulation and seed yield.

## Electronic supplementary material

Supplementary information
