# Supplementary material for: Identification of Purple Acid Phosphatases in Chickpea and Potential Roles of CaPAP7 in Seed Phytate Accumulation
Source: Sci Rep. 2017 Sep 8;7:11012. doi: 10.1038/s41598-017-11490-9 (PMC5591292; doi:10.1038/s41598-017-11490-9)
Supplement: Supplementary file 1 — Supplementary information [file 41598_2017_11490_MOESM1_ESM.pdf]

Identification of Purple Acid Phosphatases in Chickpea and Potential Roles of CaPAP7 in Seed Phytate Accumulation

Jyoti Bhadouria, Ajit Pal Singh, Poonam Mehra, Lokesh Verma, Rishi Srivastawa, Swarup K. Parida and Jitender Giri*

National Institute of Plant Genome Research, Aruna Asaf Ali Marg, New Delhi-110067, India

*Correspondence

Jitender Giri

National Institute of Plant Genome Research

Aruna Asaf Ali Marg, New Delhi 110067, India

E mail: jitender@nipgr.ac.in

91-11-26735227

Fax No. : 91-11-26742658

Keywords: PAPs, chickpea, Pi deficiency, seed phytate, association mapping

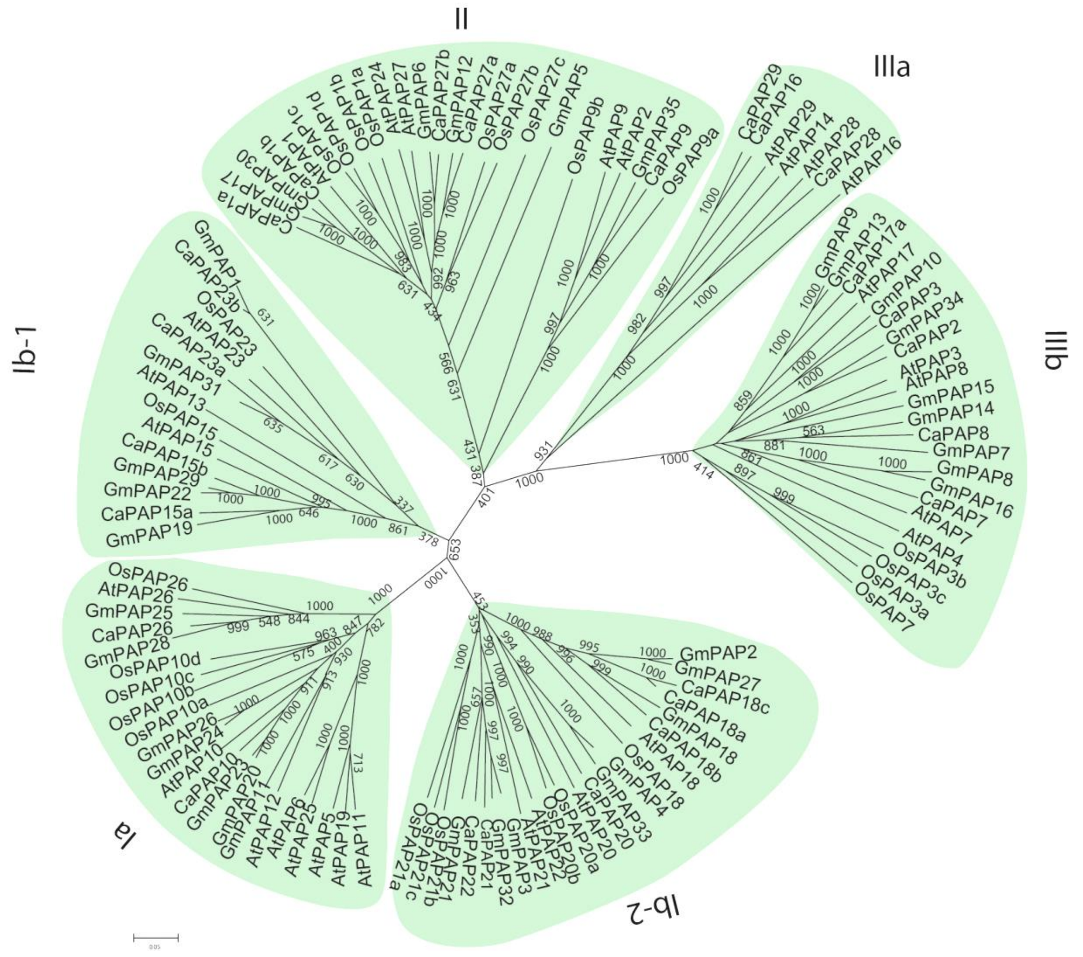

Figure S1. Phylogenetic relationship of rice (OsPAPs), soybean (GmPAPs), chickpea(CaPAPs) and Arabidopsis (AtPAPs). The phylogenetic tree was constructed using PAPs amino acid sequences from respective plants. The sequences of PAPs were aligned using ClustalX2 and the phylogenetic tree was constructed using NJ method with bootstrap value 1000. Bootstrap value is mentioned at each node.

A

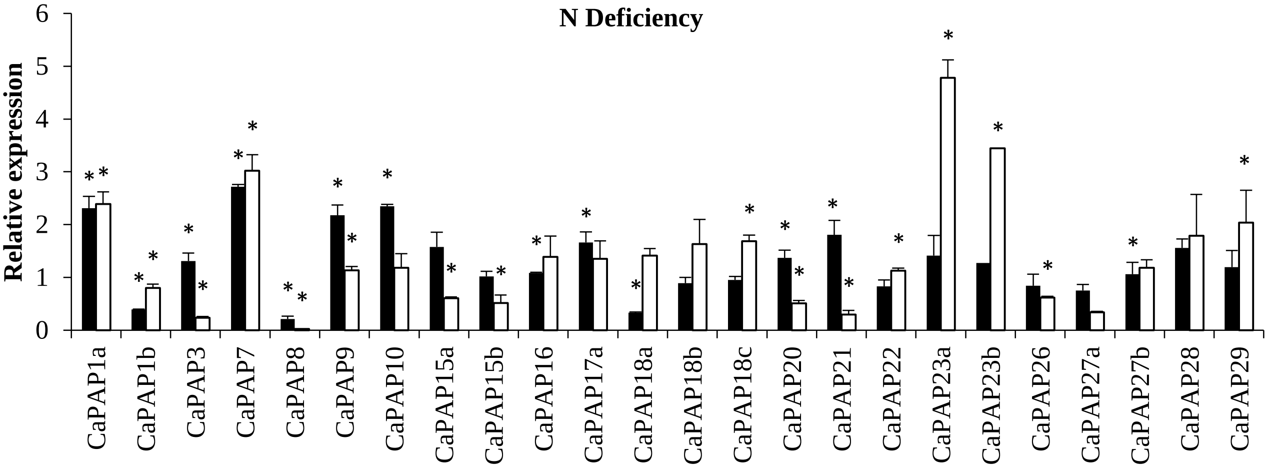

B

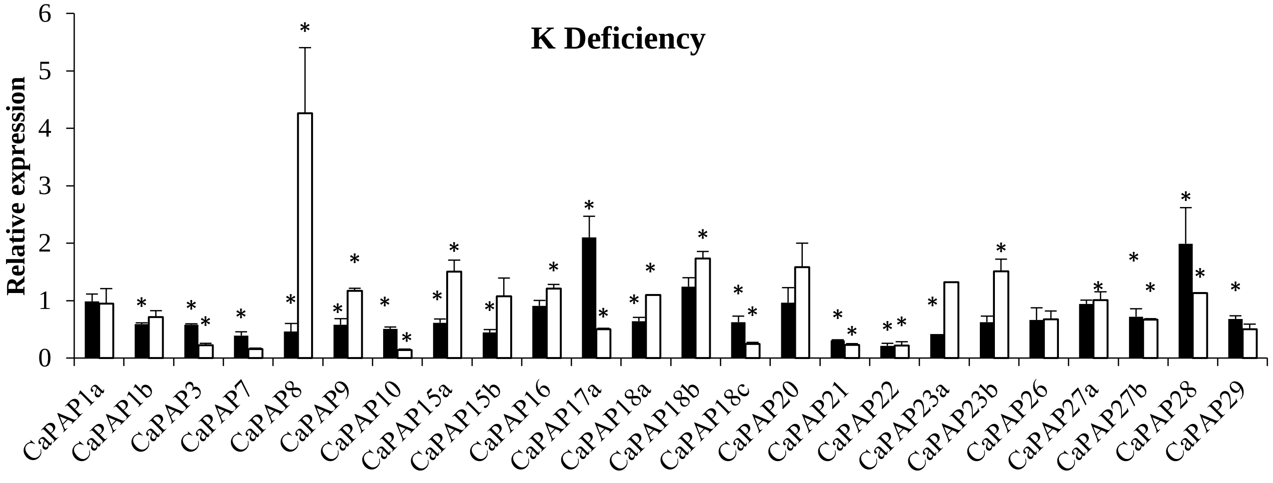

Figure S2. Relative expression profile of *CaPAPs* under **(**A) N and (B) K deficiency after 7d (early response) and 15d (late response) of respective treatments. qRT-PCR was used for quantification of gene expression. The relative gene expression in stressed plants was calculated considering untreated plants as control. EF1α was used as endogenous control. Error bars represent SE of average of two replicates (n=3). * $p < 0.05$.

A

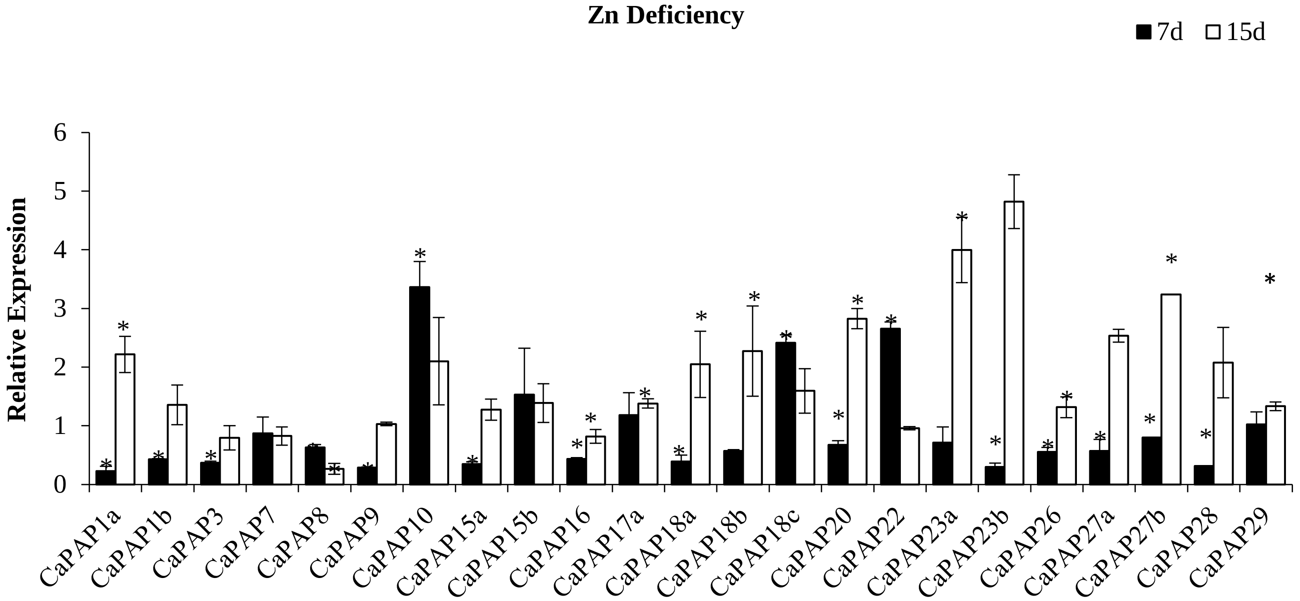

B

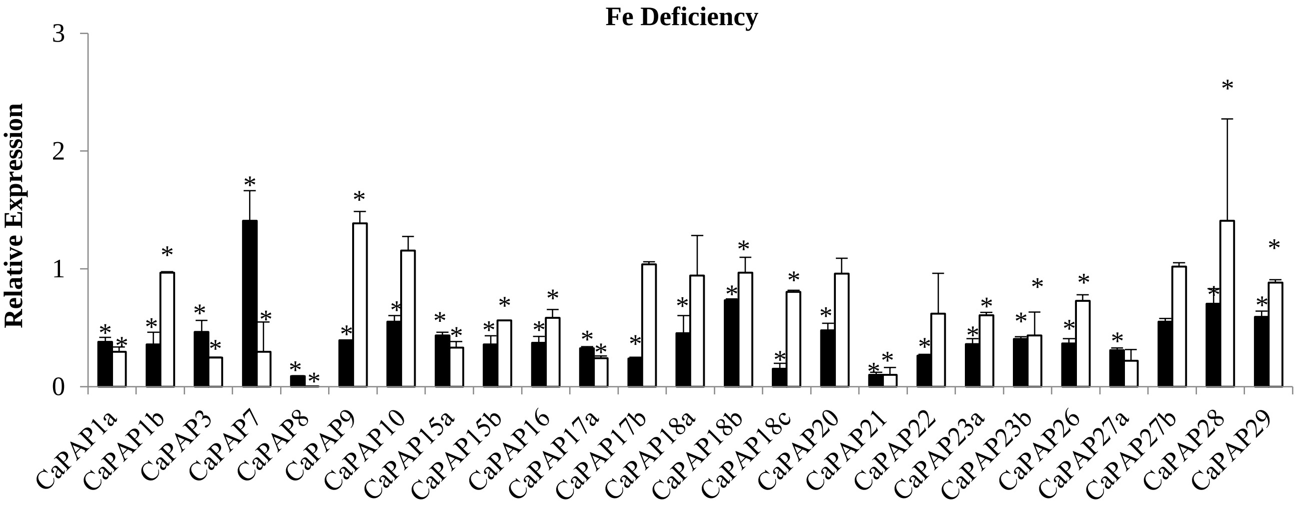

Figure S3. Relative expression profile of *CaPAPs* under **(**A) Zn, and (B) Fe deficiency after 7d (early response) and 15d (late response) of respective treatments. qRT-PCR was used for quantification of gene expression. The relative gene expression in stressed plants was calculated considering untreated plants as control. EF1α was used as endogenous control. Error bars represent SE of average of two replicates (n=3) * $p < 0.05$.

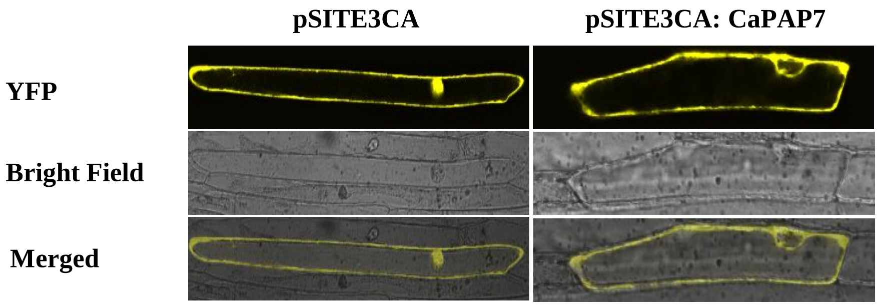

Figure S4. Subcellular localization of YFP-CaPAP7 in onion epidermal cell. Particle bombardment method was used for onion epidermal cell transformation with CaPAP7:YFP DNA coated gold particles. YFP fluorescence was analyzed with Leica SP2 confocal microscope.

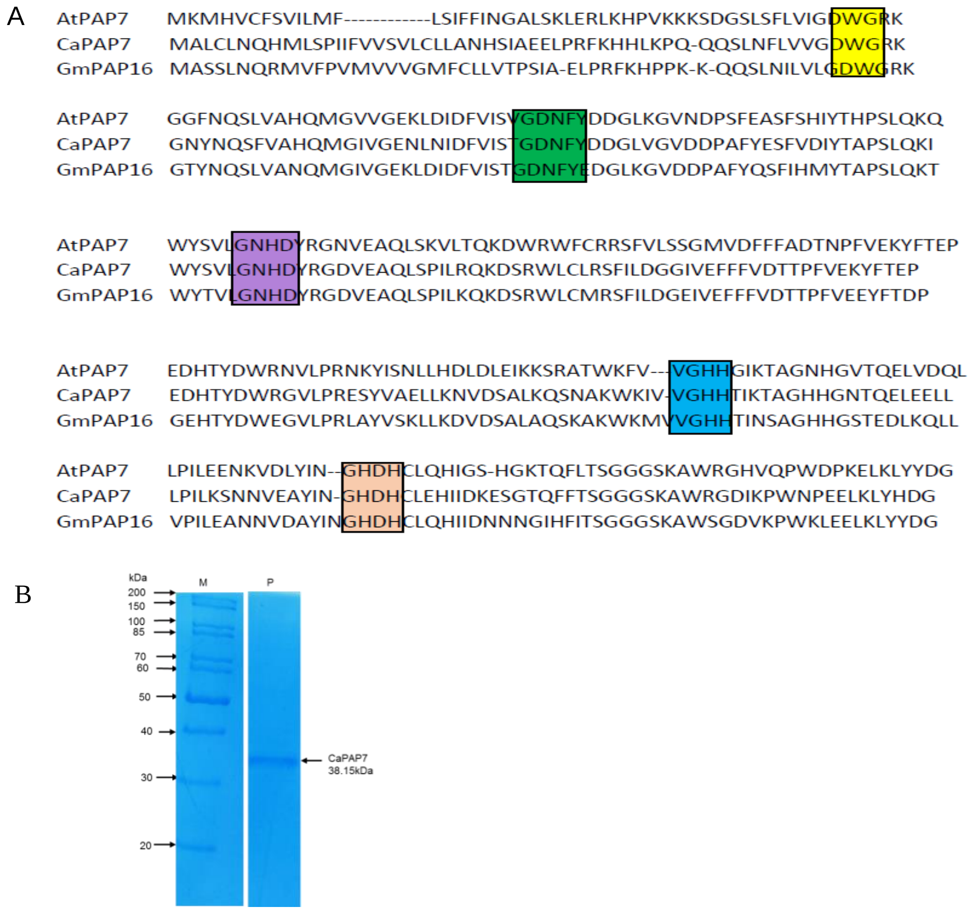

Figure S5. (A) Nearest Homologue of CaPAP7 in Arabidopsis (AtPAP7*)* and *Glycine max* (GmPAP16). All the five conserved domains (DXG/GDXXY/GNH(D/E)/VXXH/GHXH) are marked in the alignment performed with Clustal Omega. (B) SDS PAGE showing purified recombinant CaPAP7 (38.15kDa). Recombinant CaPAP7 was cloned in pET28a expression vector and expressed in BL21 (DB3.1) expression host. M-Marker, P-protein).

A **XP_004504591.1 Mass: 38349 Score: 120 Matches: 5(3) Sequences: 2(1)**
PREDICTED: purple acid phosphatase 7-like [*Cicer arietinum*]

```
  1 MALCLNQHML SPIIFVVSVL CLLANHSIAE ELPRFKHHLK PQQQSLNFLV
 51 VGDWGRKGNY NQSFVAHQMG IVGENLNIDF VISTGDNFYD DGLVGVDDPA
101 FYESFVDIYT APSLQKIWYS VLGNHDYRGD VEAQLSPILR QKDSRWLCLR
151 SFILDGGIVE FFFVDTTPFV EKYFTEPEDH TYDWRGVLPR ESYVAELLKN
201 VDSALKQSNA KWKIVVGHHT IKTAGHHGNT QELEELLLPI LKSNNVEAYI
251 NGHDHCLEHI IDKESGTQFF TSGGGSKAWR GDIKPWNPEE LKLYHDGQGF
301 MSVQITNTIA DFVFYDVFGK VLHTWTISKE HKAAE
```

B

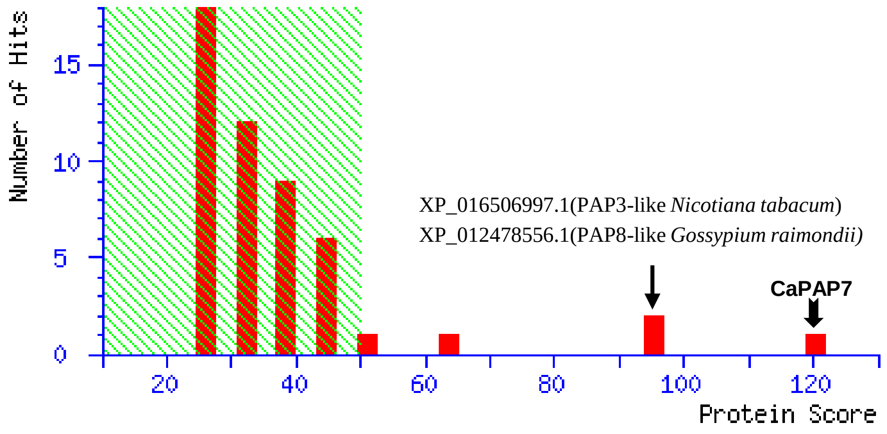

Figure S6. Confirmation of CaPAP7 using LC-MS/MS. (A) Snapshots of Mascot search result window showing identification of CaPAP7. Matched peptides in CaPAP7 protein sequence are highlighted in red. (B) Mass score histogram representing identification of CaPAP7 (indicated by block arrow) with a high mascot score of 120 at ($p<0.05$). Protein band was identified and analyzed using 4000Q TRAP LC/MS/MS and Mascot search engine (version 2.1).

Table S1. List of organisms and number of sequences

Organism Name	No. of PAP sequences
Arabidopsis thaliana	29
Oryzae sativa	26
Solanum lycopersicum	21
Hordeum vulgare	32
Medicago trunculata	30
Physcometrilla patens	20
Vitis vinifera	31
C.elegans	8
Homo sapiens	5
Dictyostelium discordeum	8
Vigna radiata	2
Phaseolus vulgaris	26
Rhizobium galegae	1
Glycine max	35
Zea mays	39
Solanum tuberosum	28
Chlamydomonas reinharbtii	7
Total	348

Table S2. List of primers used in qRT-PCR analysis and gene cloning

CaPAP locus	**Primer**	**For cDNA Amplification (5'-3')**
Ca_00785	qCaPAP21F	TCGTGAGGGACTTGCATTAAAG
	qCaPAP21R	AAGCTTGGTTCCCGAAACAA
Ca_00788	qCaPAP20F	TGGGATCTTACACCGATTTCG
	qCaPAP20R	CAAGTCCCCTTTAAGCCATTGA
Ca_05071	qCaPAP7F	TTGCTTGGAGCACATAATTGACA
	qCaPAP7R	GATCCGCCTCCACTTGTGA
Ca_05073	qCaPAP8_F	GGAGAGGCATAGGGCCTAGAA
	qCaPAP8R	AAGCCAAATCCACATCCTTGA
Ca_06578	qCaPAP9F	AAGACCGGATCATCCCAATG
	qCaPAP9R	CCGCACGGTACAGAGATCGT
Ca_06712	qCaPAP26F	AGCATGCGAGTGGTTTTCG
	qCaPAP26R	GACATGGCCAGCAAAGATCA
Ca_08756	qCaPAP27bF	ATCGCCTGCTGGAACATTG
	qCaPAP27bR	CAGTTCGTGCAGGTGAACCA
Ca_08833	qCaPAP28F	TGCGTCGTGTTTCTCATGAAC
	qCaPAP28R	GCGGTGTAGGATGAAGAGGATCT
Ca_10328	qCaPAP3F	CCCTGTCAACGCAGCAGAT
	qCaPAP3R	TCCTTCCCCAGTCTCCAATG
Ca_10489	qCaPAP23aF	ACCGGTGGAAAGGGTGTTC
	qCaPAP23aR	GGTCTCTCTGATTGGTGCATCTG
Ca_10490	qCaPAP23bF	TGCAGATGAACCTGGAAAGTGT
	qCaPAP23bF	TGACAAACTCCTCCAAATTCAGGTA
Ca_10865	qCaPAP18aF	GAATTCCGCGAAGCCAGTT
	qCaPAP18aR	GAAGGCATGAGTTGAGTTTACAATCT
Ca_11414	qCaPAP15bF	GATCCCTGCGGTCCTGTTTA
	qCaPAP15bR	TGCCATCTTTTCCCGGTTAC
Ca_14544	qCaPAP1bF	TGTTGGATGGCGTGATCCT
	qCaPAP1bR	TTGTTCGGCCACAACTCCTT
Ca_16657	qCaPAP27aF	TTTCTCCGGCAAACTTCAATTC
	qCaPAP27aR	CTCTAACTTGCCAACGCCATT
Ca_18569	qCaPAP1aF	TGCTGGGACACTGACTTTCACT
	qCaPAP1aR	TCACGCCCATCCTACTGATTTTG
Ca_18999	qCaPAP17bF	GACCATTGCCTCCAACACATAA
	qCaPAP17bR	GCCTGCTCCACTTGTTAAATACAA
Ca_20993	qCaPAP29F	TGCAGTTGCATCAAACATTCC
	qCaPAP29R	GACCCTTCTTGGTCATGGTTTC
Ca_20994	qCaPAP16F	AATCAATGGATGCTGCCTTTG
	qCaPAP16R	AAAACAGCCACCCAAGGAATG
Ca_21162	qCaPAP18bF	GGTCACGGTGAGCTAAAGATTGTA
	qCaPAP18bR	ATTCCGGTGCCAACTCCAA
Ca_21435	qCaPAP15aF	CTGTCACCGTTCCGTTCGA
	qCaPAP15aR	ATCGGTATCGGGCAAGTCAA
Ca_22060	qCaPAP10F	GAGGATGAACCGGGATCGA
	qCaPAP10R	CTTCCGATGGCTGTTCTCACT
Ca_22181	qCaPAP17aF	TCAATTGAGTCCATTCCTTAGACAAA
	qCaPAP17aR	CAGCAAGCTCTGAATCTACAATAAAAG
Ca_25726	qCaPAP22F	TCATCAACCTTTTTGGGATTCA
	qCaPAP22R	TGGTCGACGACTTGCATACG
Ca_27013	qCaPAP18cF	TCCGCGAAGCCAGTTTTG
	qCaPAP18cR	AGAAGGCGTGAGTTGAGTTTACAA
CaEF1	qEF1a_F	TCCACCACTTGGTCGTTTTG
	qEF1a_R	CTTAATGACACCGACAGCAACAG
Ca_05071	Ca_PAP7_pET28a_F	GACTCATATGGCTTTGTGTTTAAACCAAC
	Ca_PAP7_pET28a_R	CTGAGGATCCCTATTCAGCTGCTTTATGTTCTTTG

Table S3 Five conserved motifs {**D**XG/G**D**XX**Y**/G**N**H(D/E)/VXX**H**/G**H**X**H**} in the CaPAPs

	**Conserved Motifs**					
CaPAPs	**D**XG	G**D**XX**Y**	G**N**H(D/E)	G**H**X**H**	VXX**H**	FXXH
CaPAP1a	DPG,DMG,DSG,DYG	GDICY	GNHE	GHVH	VNIH,VWSH	
CaPAP1b	DQG,DPG,DMG,DYG,DFG	GDLCY	GNHE	GHVH	VAIH	FLAH
CaPAP3	DWG,DDG	GDNFY	GNHD	GHDH	VGHH	
CaPAP7	DWG ,DDG,DGG	GDNFY	GNHD	GHVH	VGHH	FVAH
CaPAP8	DWG,DNG	GDNFY	GNHD	GHDH	VFEH,VVGH	
CaPAP9	DGG,DPG,DMG,DYG	GDISY	GNHE,GNHD	GHVH	VAYH	
CaPAP10	DLG,DGG	GDLSY	GNHE	GHVH	VLMH	
CaPAP15a	DLG	GDIQY	GNHE,GNRE	GHVH	IIHH	
CaPAP15b	DGG,DLG	GDASY,GDAIY	GNHE,GNRE	GHVH	IIHH	
CaPAP16	DSG,DKG	GDNIF	GNHD	GHDH	-	
CaPAP17a	DWG,DDG	GDNFY	GNHD	GHDH	VVGH	
CaPAP17b	DWG	GDNFY	GNHD	GHDH	VVGH	
CaPAP18a	DLG	GDLSY	GNRE	-	VLFH	
CaPAp18b	DLG	GDLSY	GNHE	GHVH	VLFH	
CaPAP18c	DLG,DGG	GDLSY	GNHE,GNRE	GHVH	VLFH,VNVH,VLFH	
CaPAP20	DLG	GDLSY	GNHD,GNRE	GHIH,GHVH	VLVH	
CaPAP21	DLG	GDLSY	GNHE,GNRE	GHVH	ILLH	
CaPAP22	DLG	GDLSY	GNHE,GNRE	GHVH	VLLH	FLLH
CaPAP23a	DLG	GDLTY	-	-	IIHH	
CaPAP23b	DGG	GDQIY	GNIE	GHVH	-	
CaPAP26	DLG,DVG,DGG,DDG	GDLSY	GNHE	GHVH	VLMH	
CaPAP27a	DMG,DPG,DYG	GDLPY	GNHE	GHVH	IAIH	FSAH
CaPAP27b	DPG,DYG,DSG,DMG	GDISY	GNHE	GHVH	VFAH	
CaPAP28	DSG	GDNIF	GNHD	GHDH	AILH	
CaPAP29	DSG	GDNIF	GNHD	GHDH	VMKH	

Residues in bold letters indicate metal binding residues essential for PAP activity.

Table S4. *In-silico* analysis of *CaPAPs.* Chromosomal localization, orientation, CDS length, exon number, number of amino acid, protein molecular weight and their Arabidopsis homologue

CaPAPs	Chr Locus	Position	Strand	CDS	No. of exon	No. of amino acid	Molecular weight(kD)	pI	Probable Name	Arabidopsis Homologue
**Ca18569**	Chr2	15174612:15181081	+	1839	12	612	68.9	6.98	CaPAP1a	AtPAP1
**Ca14544**	Chr7	22157983:22164686	-	1785	12	594	66.39	6.44	CaPAP1b	AtPAP1
**Ca10328**	Chr6	2073980:2075557	+	1008	7	335	38.05	5.98	CaPAP3	AtPAP3
**Ca05071**	Chr6	12978476:12982866	-	1008	7	335	38.15	5.52	CaPAP7	AtPAP7
**Ca05073**	Chr6	12990415:12995717	+	975	7	324	36.9	5.28	CaPAP8	AtPAP8
**Ca06578**	Chr6	19713878:19716828	-	1974	2	657	73.91	6.3	CaPAP9	AtPAP9
**Ca22060**	Chr6	33781022:33784934	-	1215	8	404	47.19	6.25	CaPAP10	AtPAP10
**Ca21435**	Scaffold128	146651:153277	+	1665	7	554	63.49	5.23	CaPAP15a	AtPAP15
**Ca11414**	Chr5	41984839:41990090	-	1560	7	519	58.35	5.13	CaPAP15b	AtPAP15
**Ca20994**	Chr7	34262894:34265814	+	1272	5	423	47.52	5.89	CaPAP16	AtPAP16
**Ca22181**	Scaffold88	201739:204255	-	998	7	332	38.02	5.06	CaPAP17a	AtPAP17
**Ca18999**	Scaffold40	1030859:1033543	-	990	7	329	37.73	8.55	CaPAP17b	AtPAP17
**Ca10865**	Chr4	47145345:47147113	-	999	5	332	37.67	5.46	CaPAP18a	AtPAP18
**Ca21162**	Chr5	22045511:22049272	-	1287	5	428	48.52	5.9	CaPAP18b	AtPAP18
**Ca27013**	Scaffold6367	21891:26350	+	2301	9	766	87.33	5.45	CaPAP18c	AtPAP18
**Ca00788**	Chr3	35141804:35143185	+	1107	4	368	41.67	5.24	CaPAP20	AtPAP20
**Ca00785**	Chr3	35117435:35120745	-	1335	5	444	50.68	5.5	CaPAP21	AtPAP21
**Ca25726**	Scaffold1710	121883:126468	-	1335	5	444	51.15	5.82	CaPAP22	AtPAP22
**Ca10489**	Chr2	3734966:3736818	-	741	3	246	27.07	5.7	CaPAP23a	AtPAP23
**Ca06712**	Chr7	6389656:6393906	+	1362	8	453	52.27	5.98	CaPAP26	AtPAP26
**Ca08756**	Chr1	20858417:20862942	-	1872	12	623	69.96	6.02	CaPAP27b	AtPAP27
**Ca16657**	Chr5	29751506:29758150	+	1695	12	564	63.42	6.39	CaPAP27a	AtPAP27
**Ca08833**	Chr1	20111337:20115043	+	1191	4	396	44.96	6.9	CaPAP28	AtPAP28
**Ca20993**	Chr7	34267082:34269769	+	1122	4	373	41.4	7.6	CaPAP29	AtPAP29
**Ca10490**	Chr2	3732188:3733389	-	462	3	153	17.33	5.57	CaPAP23b	AtPAP23

Table S5. Summary of CaPAPs localization, presence of glycosylation sites and *P1BS* element

**CaPAPs**	**Signal Peptide (Length, Cleave site)**	**Localisation**	**Glycosylation site**	***P1BS* element**	
				Number	Location
CaPAP1a	23,VWS-HG	Extracellular/Lysosomal	5	4	-2272,-2341,-2351,-2547
CaPAP1b	17,VVS-DV	Extracellular	7	1	-546
CaPAP3	30,VSA-EL	Extracellular	2	1	-1080
CaPAP7	29,SIA-EE	Cytoplasmic	2	0	-
CaPAP8	20,SSA-VL	Extracellular	2	1	-21
CaPAP9	21,NLA-QS	Extracellular/Lysosomal	7	1	-162
CaPAP10	No	Extracellular	6	2	-264,1940
CaPAP15a	35,TNC-HI	Extracellular/Plasma Membrane/Lysosomal	6	3	-1247,-1880,-2117
CaPAP15b	No	Extracellular	8	1	-548
CaPAP16	15,TSS-SS	Cytoplasmic	5	0	-
CaPAP17a	No	Extracellular/Peroxisome	2	4	-602,-623,-666,-1981
CaPAP17b	25,IFA-EL	Extracellular	3	1	-2385
CaPAP18a	No	Extracellular	2	0	-
CaPAp18b	20,TIA-DD	Extracellular/Nuclear	1	0	-
CaPAP18c	22,ITA-EY	Extracellular/Plasma Membrane	2	1	-1504
CaPAP20	No	Extracellular	1	0	-
CaPAP21	29,LLS-QD	Extracellular/Cytoplasmic	3	2	-1648.-2699
CaPAP22	29,IQS-QG	Extracellular	5	3	-950,-2528,-2756
CaPAP23a	No	Extracellular	5	1	-240
CaPAP23b	No	Extracellular/Cytoplasmic	3	1	-2318
CaPAP26	24,GCA-GI	Extracellular	3	0	-
CaPAP27a	No	Extracellular/Lysosomal	5	0	-
CaPAP27b	No	Extracellular/Lysosomal	8	1	-1939
CaPAP28	No	Plasma Membrane	3	1	-2260
CaPAP29	21,STT-CV	Extracellular	3	3	-1621,-2418,-2588

Text S1. Protein sequences of PAPs from different organisms used for generating HMM profile.

>AtPAP11
MELSHLALVCAAIAFSSIFVVSQAGITSTHARVSEPSEEMSLETFPPPAGYNAPEQVHITQGDNAG
RAMIISWVMPLNEDGSNVVTYWIASSDGSDNKNAIATTSSYRYFNYTSGYLHHATIKKLEYDPS
KSRSRCSLHIRYYSDLGQTYASNQTLYNYMSNPKGQAVLFVGDLSYADDHPNHDQRKWDSYG
RFVEPSAAYQPWSWAAGNYEIDYAQSISETQPFKPYKNRYHVPYKASQSTSPLWYSIKRASTYII
VLSSYSAYDKYTPQNSWLQDELKKVNRSETSWLIVLVHAPWYNSNNYHYMEGESMRVTFEPW
FVENKVDIVFAGHVHAYERSKRISNIHYNITDGMSTPVKDQNAPIYITIGDGGNIEGIANSFTDPQP
SYSAFREASFGHALLEIKNRTHAHYTWHRNKEDEAVIADSIWLKKRYYLPEEETA
>AtPAP12
MSSRSDLKIKRVSLIIFLLSVLVEFCYGGFTSEYVRGSDLPDDMPLDSDVFEVPPGPNSPQQVHVT
QGNHEGNGVIISWVTPVKPGSKTVQYWCENEKSRKQAEATVNTYRFFNYTSGYIHHCLIDDLEF
DTKYYYEIGSGKWSRRFWFFIPPKSGPDVPYTFGLIGDLGQTYDSNSTLSHYEMNPGKGQAVLFV
GDLSYADRYPNHDNNRWDTWGRFVERSVAYQPWIWTAGNHEIDFVPDIGEIEPFKPFMNRYHT
PHKASGSISPLWYSIKRASAYIIVMSCYSSYGIYTPQYKWLEKELQGVNRTETPWLIVLVHSPFYS
SYVHHYMEGETLRVMYEQWFVKYKVDVVFAGHVHAYERSERVSNIAYNIVNGLCEPISDESAPI
YITIGDGGNSEGLLTDMMQPQPKYSAFREASFGHGLLEIKNRTHAYFSWNRNQDGNAVAADSV
WLLNRFWRAQKKTWLDAF
>AtPAP13
MVVKYTMSMSFFVIFASTVTIIVHGFPSTLDGPLNPVTAPLDPNLNPIAFDLPESDPSFVKPISEFLL
PEQISVSLSYSFDSVWISWVTGEYQIGEKDSAPLDPNCVQSIVQYREFDVRRTRKQNATGHSIVY
NQQYSSENGFMNYTSGIIHHVQLTGLKPNTLYRYQCGDPSLSAMSKEYYFRTMPKSTSENYPHRI
VVAGDLGLTYNTSTVLGHILSNHPDLVVLLGGFSYADTYLANKTKLDCSSCHCDQNGTSSDCGS
CYSSGETYQPRWDYWGRFMEPLTANVPTMMVAGEHEIEPQTENNLTFAAYSSRFAFPSNESGSF
SPLYYSFNAGGAHFIVLNSYTLYDNSSDQYIWLESDLIKINRSETPWVVATWSLPWYSTFKGHYR
EAESMRIHLEDLLYNYRVDIVFNSHVDAYERSNRVYNYTLDQCGPVYITTGAGGAGKLETQHV
DDPGNIPDPSQNYSCRSSGLNSTLEPVKDETCPVKQPEYSAYRESSFGFGILEVKNETHALWSWN
RNQDLYYLAADVIHIVRQPEMCSVCN
>AtPAP14
MEETRRRFVISSVLSVSLIYLCLSTCHVSAFDFGRRQLRFNTDGRFKILQVSDMHYGFGKETQCSD
VSPAEFPYCSDLNTTSFLQRTIASEKPDLIVFSGDNVYGLCETSDVAKSMDMAFAPAIESGIPWVA
ILGNHDQESDMTRETMMKYIMKLPNSLSQVNPPDAWLYQIDGFGNYNLQIEGPFGSPLFFKSILN
LYLLDGGSYTKLDGFGYKYDWVKTSQQNWYEHTSKWLEMEHKRWPFPQNSTAPGLVYLHIPM
PEFALFNKSTEMTGVRQESTCSPPINSGFFTKLVERGEVKGVFSGHDHVNDFCAELHGINLCYAG
GAGYHGYGQVGWARRVRVVEAQLEKTMYGRWGAVDTIKTWKRLDDKNHSLIDTQLLWTKNT
TLEPNFGFSCSTIPQH
>AtPAP10
MGRVRKSDFGSIVLVLCCVLNSLLCNGGITSRYVRKLEATVDMPLDSDVFRVPCGYNAPQQVHI
TQGDVEGKAVIVSWVTQEAKGSNKVIYWKENSTKKHKAHGKTNTYKFYNYTSGFIHHCPIRNL
EYDTKYYYVLGVGQTERKFWFFTPPEIGPDVPYTFGLIGDLGQSYDSNITLTHYENNPTKGQAV
LFVGDISYADTYPDHDNRRWDSWGRFAERSTAYQPWIWTTGNHELDFAPEIGENRPFKPFTHR
YRTPYRSSGSTEPFWYSIKRGPAYIIVLASYSAYGKYTPQYQWLEEEFPKVNRTETPWLIVLMHS
PWYNSYDYHYMEGETMRVMYEAWFVKYKVDVVFAGHVHAYERSERVSNIAYNVVNGICTPV
KDQSAPVYITIGDGGNIEGLATKMTEPQPKYSAFREASFGHAIFSIKNRTHAYGWHRNHDGYA
VEGDRMWFYNRFWHPVDDSPSCNS
>AtPAP15

MTFLLLLLFCFLSPAISSAHSIPSTLDGPFVPVTVPLDTSLRGQAIDLPDTDPRVRRRVIGFEPEQISL
SLSSDHDSIWVSWITGEFQIGKKVKPLDPTSINSVVQFGTLRHSLSHEAKGHSLVYSQLYPFDGLL
NYTSGIIHHVRITGLKPSTIYYYRCGDPSRRAMSKIHHFRTMPVSSPSSYPGRIAVVGDLGLTYNT
TDTISHLIHNSPDLILLIGDVSYANLYLTNGTSSDCYSCSFPETPIHETYQPRWDYWGRFMENLTS
KVPLMVIEGNHEIELQAENKTFEAYSSRFAFPFNESGSSSTLYYSFNAGGIHFVMLGAYIAYDKSA
EQYEWLKKDLAKVDRSVTPWLVASWHPPWYSSYTAHYREAECMKEAMEELLYSYGTDIVFNG
HVHAYERSNRVYNYELDPCGPVYIVIGDGGNREKMAIEHADDPGKCPEPLTTPDPVMGGFCAW
NFTPSDKFCWDRQPDYSALRESSFGHGILEMKNETWALWTWYRNQDSSSEVGDQIYIVRQPDRC
PLHHRLVNHC
>AtPAP17
MNSGRRSLMSATASLSLLLCIFTTFVVVSNGELQRFIEPAKSDGSVSFIVIGDWGRRGSFNQSLVA
YQMGKIGEKIDLDFVVSTGDNFYDNGLFSEHDPNFEQSFSNIYTAPSLQKQWYSVLGNHDYRGD
AEAQLSSVLREIDSRWICLRSFVVDAELVEMFFVDTTPFVKEYYTEADGHSYDWRAVPSRNSYV
KALLRDLEVSLKSSKARWKIVVGHHAMRSIGHHGDTKELNEELLPILKENGVDLYMNGHDHCL
QHMSDEDSPIQFLTSGAGSKAWRGDINPVTINPKLLKFYYDGQGFMSARFTHSDAEIVFYDVFGE
ILHKWVTSKQLLHSSV
>AtPAP18
MEKWGILLLVTLSVSIIFTSAAADDYVRPKPRETLQFPWKQKSSSVPEQVHISLAGDKHMRVTW
VTNDKSSPSFVEYGTSPGKYSYLGQGESTSYSYIMYRSGKIHHTVIGPLEADTVYYYRCGGEGPE
FHLKTPPAQFPITFAVAGDLGQTGWTKSTLDHIDQCKYAVHLLPGDLSYADYMQHKWDTFGEL
VQPLASVRPWMVTQGNHEKESIPFIVDEFVSFNSRWKMPYEESGSNSNLYYSFEVAGVHAIMLG
SYTDYDRYSDQYSWLKADLSKVDRERTPWLIVLFHVPWYNSNNAHQHEGDEMMAEMEPLLYA
SGVDIVFTGHVHAYERTKRVNNGKSDPCGPVHITIGDGGNREGLARKYKDPSPEWSVFREASFG
HGELQMVNSTHALWTWHRNDDDEPTRSDEVWLNSLVNSGCLKKRPQELRKMLLEP
>AtPAP1
MRESLVAILVTVISVLGAIHQVKSHEDQPLSGIAVHKITFGLNEKAYVKASPTVLGSNGQHSELVL
VQYSSPKPSDDDWIGVFSPADFNASTCPGDNKMVQPPRLCSAPVKFQYANFSNPRYTNTGTGSL
KLQLINQRSDFSFALFSGGLLNPKLVAISNKVAFENPNAPVYPRLALGKEWDEMTVTWTSGYGL
NLAEPVVEWGVKGGERKLSPAGTLTFARNSMCGAPARTVGWRDPGYIHTAFLKELWPNSKYTY
RVGHRLSNGALIWSKEYQFKSSPFPGQNSVQQVVIFGDMGKAEVDGSSEYNDFQRASLNTTKQL
IKDLKKTDAVFHIGDICYANGYLSQWDQFIAQIEPIASTVPYMIASGNHERVWPNSGSFYEGLDS
GGECGVPAETMFYVPAQNRAKVWYSSDYGMFRFCVADTEHDWREGTEQYNFIEHCLASVDRQ
KQPWLIFLAHRVLGYSSTYFYAEEGSFAEPMGRESLQKLWQKYKVDIAIYGHAHNYERTCPVYQ
SVCTSHEKSNYKAPLNGTIHIVAGGGGAGLAEFSDLQPNWSLFRDYDYGFLKLTAIDHSNLLFEY
KKSSDGRVHDSFTISKDYRDILACAVDSCPATTLAS
>AtPAP22
MKLFGLFLSFTLLFLCPFISQADVPELSRQPPRPIVFVHNDRSKSDPQQVHISLAGKDHMRVTFITE
DNKVESVVEYGKQPGKYDGKATGECTSYKYFFYKSGKIHHVKIGPLQANTTYYYRCGGNGPEF
SFKTPPSTFPVEFAIVGDLGQTEWTAATLSHINSQDYDVFLLPGDLSYADTHQPLWDSFGRLVEP
LASKRPWMVTEGNHEIEFFPIIEHTTFKSYNARWLMPHTESFSTSNLYYSFDVAGVHTVMLGSYT
DFDCESDQYQWLQADLAKVDRKTTPWVVVLLHAPWYNTNEAHEGEGESMREAMESLLFNAR
VDVVFSGHVHAYERFKRVYNNKADPCGPIHITIGDGGNREGLALSFKKPPSPLSEFRESSFGHGRL
KVMDGKRAHWSWHRNNDSNSLLADEVWLDSLSTSSSCWPSSRSNDEL
>AtPAP25
MRMNKILLVFVFLSIATVINSGTTSNFVRTAQPSTEMSLETFPSPAGHNAPEQVHIVQGDYNGRGI
IISWVTPLNLAGSNVVTYWKAVDGDVKPKKKRGHASTSSYRFYDYTSGFLHHATIKGLEYDTKY
IYEVGTDGSVRQFSFTSPPKVGPDVPYTFGIIGDLGQTLASNETLYHYMSNPKGQAVLFPGDLSY

ADDHPNHDQRKWDSWGRFVEPCAAYQTFIYAAGNHEIDFVPNIGEPHAFKPYIHRYHNAYKAS
KSISPLWYSIRRASAHIIVLSSYSAYGKYTPQYVWLEQELKKVNREETPWLIVMVHSPWYNSNNY
HYMEGESMRAMFESWFVNSKVDLVLSGHVHSYERSERVSNIKYNITNGLSYPVKDPSAPIYITIG
DGGNIEGIANSFTDPQPSYSAYREASFGHAVLEIYNRTHAYYTWHRNQDNEPVAADSIMLHNRY
FFPVEELESGNTRA
>AtPAP20
MVKVLGLVAILLIVLAGNVLSYDRQGTRKNLVIHPTNEDDPTFPDQVHISLVGPDKMRISWITQS
SISPSVVYGTVSGKYEGSANGTSSSYHYLLIYRSGQINDVVIGPLKPNTVYYYKCGGPSSTQEFSF
RTPPSKFPIKFAVSGDLGTSEWSKSTLEHVSKWDYDVFILPGDLSYANMYQPLWDTFGRLVQPL
ASQRPWMVTHGNHELEKIPILHSNPFTAYNKRWRMPFEESGSSSNLYYSFNVYGVHIIMLGSYTD
FEPGSEQYQWLENNLKKIDRKTTPWVVAVVHAPWYNSNEAHQGEKESVEMKESMETLLYKAR
VDLVFAGHVHAYERFSRVYQDKFDKCGPVYINIGDGGNLEGLATKYRDPNPEISLFREASFGHG
QLVVENATHARWEWHRNDDDVSVEKDSVWLTSLLADSSCKI
>AtPAP26
MNHLVIISVFLSSVLLLYRGESGITSSFIRSEWPAVDIPLDHHVFKVPKGYNAPQQVHITQGDYDG
KAVIISWVTPDEPGSSQVHYGAVQGKYEFVAQGTYHNYTFYKYKSGFIHHCLVSDLEHDTKYY
YKIESGESSREFWFVTPPHVHPDASYKFGIIGDMGQTFNSLSTLEHYMESGAQAVLFLGDLSYAD
RYQYNDVGVRWDSWGRFVERSTAYQPWLWSAGNHEVDYMPYMGEVTPFRNYLQRYTTPYLA
SKSSSPLWYAVRRASAHIIVLSSYSPFVKYTPQWHWLSEELTRVDREKTPWLIVLMHVPIYNSNE
AHFMEGESMRAAFEEWFVQHKVDVIFAGHVHAYERSYRISNVRYNVSSGDRYPVPDKSAPVYIT
VGDGGNQEGLAGRFTEPQPDYSAFREASYGHSTLDIKNRTHAIYHWNRNDDGKKVATDEFVLH
NQYWGKNIRRRKLKKHYIRSVVGGWIAT
>AtPAP28
MNCSIGNWKHTVLYLTLIVSLLYFIESLISHKLHINYNKIRLKRSPNLPLRFRDDGTFKILQVADM
HFGMGMITRCRDVLDSEFEYCSDLNTTRFLRRMIESERPDLIAFTGDNIFGSSTTDAAESLLEAIGP
AIEYGIPWAAVLGNHDHESTLNRLELMTFLSLMDFSVSQINPLVEDETKGDTMRLIDGFGNYRVR
VYGAPGSVLANSTVFDLFFFDSGDREIVQGKRTYGWIKESQLRWLQDTSIQGHSQRIHVNPPALA
FFHIPILEVRELWYTPFIGQFQEGVACSIVQSGVLQTFVSMGNVKAAFMGHDHVNDFCGTLKGV
WFCYGGGFGYHAYGRPNWHRRARVIEAKLGKGRDTWEGIKLIKTWKRLDDEYLSKIDEQVLW
ETSDSFLK
>AtPAP24
MARVLGVLLCLLALFSSSLCLDHANGRGDQALAQINVYETSLALDSSVKLHASPQVLGSQGEDT
EWVNLAISNPKPTSDDWIGVFSPAKFDSGNCWPTSGGKEKTPYICSSPIKYMYCNSHPDYMKSG
NVTLKFQIINQRADVSFALFSNGVQEPHLLGVSNPVAFFNPKAPVYPRLALGKNWDEMTVTWTS
GYNIDEAVPFIEWSAKGLPARRSPAGTLTFNRNSMCGNPARGVGWRDPGFFHTSFLKELWPNRE
YIYRLGHDLVNGSTIWSKNYTFVSSPYPGQDSKQRVIIFGDMGKGERDGSNEYNDYQPGSLNTT
DQVIKDLKDIDIVFHIGDLTYSNGYLSQWDQFTAQVQPIASTVPYMIASGNHERDWPDTGSFYAG
TDSGGECGVPAETMFYFPAENRAKFWYKTDYGMFRFCVADSEHDWREGTEQYKFIENCLATVD
RKTQPWLIFIAHRVLGYSTNDWYGKEGTFEEPMGRESLQKLWQKYKVDLAFYGHVHNYERTCP
IYESQCVNNDKDHYSGTFKGTIHVVVGGAGSHLSPFSSLVPKWSLVRDYDFGFVKLTASDHSSLL
FEYKKSSTGQVYDSFNISRDYRDVLACTHDSCEPTTSAG
>AtPAP3
MTYIYRDTKITTKSTIPFLIFFLFCFSNLSMATLKHKPVNLVFYVYNLIIIFSSHSSTAELRRLLQPSK
TDGTVSFLVIGDWGRRGSYNQSQVALQMGEIGEKLDIDFVISTGDNFYDNGLTSLHDPLFQDSFT
NIYTAPSLQKPWYSVLGNHDYRGDVRAQLSPMLRALDNRWVCMRSFIVNAEIVDLFFVDTTPFV
DKYFIQPNKHVYDWSGVLPRQTYLNNLLKELDVALRESVAKWKIVIGHHTIKSAGHHGNTIELE

KHLLPILQANEVDLYVNGHDHCLEHISSVDSNIQFMTSGGGSKAWKGGDVNYVEPEEMRFYYD
GQGFMSVHVSEAELRVVFYDVFGHVLHHWKKTYKEALYFAS
>AtPAP4
MSSKFDIGSLSIVMTLLICFLLLSLAPKLEAELATVQHAPNPDGSISFLVIGDWGRHGLYNQSQVA
LQMGRIGEEMDINFVVSTGDNIYDNGMKSIDDPAFQLSFSNIYTSPSLQKPWYLVLGNHDYRGD
VEAQLSPILRSMDSRWICMRSFIVDAEIAELFFVDTTPFVDAYFLSPQDQTYDWSGVSPRKSYLQT
ILTELEMGLRESSAKWKIVVGHHAIKSASIHGNTKELESLLLPILEANKVDLYMNGHDHCLQHIST
SQSPIQFLTSGGGSKAWRGYYNWTTPEDMKFFYDGQGFMSVKITRSELSVVFYDVSGNSLHKW
DTSKMLDSDFYFPL
>AtPAP6
MKNLVIFAFLFLSITTVINGGITSKFVRQALPSIEMSLDTFPSPGGYNTPEQVHLTQGDHDGRGMI
VSWVTPLNLAGSNVVTYWIATNGSDVKPAKKRAHASTKSYRFYDYSSGFLHHATIKGLEYDTK
YIYEVGTDKSVRQFSFTTPPKIGPDVPYTFGIIGDLGQTYASNETLYHYMSNPKGQAVLFAGDLS
YADDHPNHDQRKWDTWGRFMEPCAAYQPFIFAAGNHEIDFVPNIGEPHAFKPYTHRYPNAYKA
SQSTSPLWYSVRRASAHIIVLSSYSAYGKYTPQYIWLEQELKNVNREETPWLIVIVHSPWYNSNN
YHYMEGESMRVMFESWLVNSKVDLVLSGHVHAYERSERISNIKYNITNGLSSPVKDPNAPIYITI
GDGGNIEGIANSFVDPQPSYSAYREASFGHAVLEIMNRTHAQYTWHRNQDNEPVAADSIMLHNR
HFFPVEEIVSSNIRA
>AtPAP8
MDSLRDVKPIKLIFSIFCLVIILSACNSTAELPRFVQPPEPDGSLSFLVVGDWGRRGSYNQSQVALQ
MGKIGKDLNIDFLISTGDNFYDDGIISPYDSQFQDSFTNIYTATSLQKPWYNVLGNHDYRGNVYA
QLSPILRDLDCRWICLRSYVVNAEIVDIFFVDTTPFVDRYFDEPKDHVYDWRGVLPRNKYLNSLL
TDVDVALQESMAKWKIVVGHHTIKSAGHHGNTIELEKQLLPILEANEVDLYINGHDHCLEHISSI
NSGIQFMTSGGGSKAWKGDVNDWNPQEMRFYYDGQGFMSVYTSEAELRVVFYDGLGHVLHR
WSTLKNGVYSDI
>AtPAP9
MIAAVYTLFFFFLLISSVYSKATISISPQTLNRSGDIVVIKWSGVESPSDLDWLGIYSPPDSPHDHFI
GYKFLSDSPTWQSGSGSISLPLTNLRSNYTFRIFHWTQSEINPKHQDHDHNPLPGTRHLLTESNQL
NFRFAVNRPEQIHLSYTDNINEMRVVFVTGDGEEREARYGEVKDKLDNIAVARGVRYEIEHMCH
APANSTVGWRDPGWTFDAVMKNLKQGIRYYYQVGSDLKGWSEIHSFVSRNEGSEETLAFMFGD
MGCYTPYTTFIRGEEESLSTVKWILRDIEALGDDKPVIVSHIGDISYARGYSWIWDEFFTQIEPIAS
KVPYHVCIGNHEYDWPNQPWKPDWAAYVYGKDSGGECGVPYSVKFNMPGNSTEATGMVKGP
QSRNLYYSYDMGSVHFVYISTETDFLKGGKQYSFLKSDLESVNRSKTPFVVVQGHRPMYTTSRK
IRDAAIREKMIEHLEPLLVKNNVTVALWGHVHRYERFCAISNNTCGERWQGNPVHLVIGMAGK
DSQPMWEPRANHEDVPIFPQPANSMYRGGEFGYIRLVANKERLTLSYVGNHDGEVHDVVEILAS
GEVISGSDDGTKDSNFGSESDFAVLWYIEGASVMVVGVIFGYFVGFLSRKKKESGVGSSNRSWIQ
VKNEET
>AtPAP7
MKMHVCFSVILMFLSIFFINGALSKLERLKHPVKKKSDGSLSFLVIGDWGRKGGFNQSLVAHQM
GVVGEKLDIDFVISVGDNFYDDGLKGVNDPSFEASFSHIYTHPSLQKQWYSVLGNHDYRGNVEA
QLSKVLTQKDWRWFCRRSFVLSSGMVDFFFADTNPFVEKYFTEPEDHTYDWRNVLPRNKYISNL
LHDLDLEIKKSRATWKFVVGHHGIKTAGNHGVTQELVDQLLPILEENKVDLYINGHDHCLQHIG
SHGKTQFLTSGGGSKAWRGHVQPWDPKELKLYYDGQGFMSLHITHSKAKFIYYDVSGNVLHRS
SLSKRSAHL
>AtPAP19
MGLNHLTLVCSAIALLSIFVVSQAGVTSTHVRVSEPSEEMPLETFPPPACYNAPEQVHITQGDHA
GRGMIISWVTPLNEDGSNVVTYWIANSDGSDNKSALATTSSYRYFNYTSGYLYHATIKGLETLY

NYMSNPKGQAVLFAGDLSYADDHPNHDQRKWDSYGRFVEPSAAYQPWIWAAGNHEIDYAESIP
HKVHLHFGTKSNELQLTSSYSPLTQLMDELKKVNRSETPWLIVLVHAPWYNSNNYHYMEGESM
RVTFEPWFVENKVDIVFAGHVHAYERSERISNIQYNITDGMSTPVKDQNAPVYITIGDGGNIEGIA
NNFIDPQPSYSAFREASFGHAILEIKNRTHAHYTWHRNKEDEFIPEAVIADSIWLKNRYYLREEET
S
>AtPAP21
MKKMKIFGFLISFSLFFLSPFVCQANYDSNFTRPPPRPLFIVSHGRPKFYPQQVHISLAGKDHMRV
TYTTDDLNVASMVEYGKHPKKYDKKTAGESTSYTYFFYNSGKIHHVKIGPLKPNTKYYYRCGG
HGDEFSFKTPPSKFPIEFAVAGDLGQTDWTVRTLDQIRKRDFDVFLLPGDLSYADTHQPLWDSFG
RLLETLASTRPWMVTEGNHEIESFPTNDHISFKSYNARWLMPHAESLSHSNLYYSFDVAGVHTV
MLGSYTPYESHSDQYHWLQADLRKVDRKKTPWLVVVMHTPWYSTNKAHYGEGEKMRSALES
LLYRAQVDVVFAGHVHTYERFKPIYNKKADPCGPMYITIGDGGNREGLALRFKKPQSPLSEFRES
SFGHGRLRIIDHKRAHWSWHRNNDEMSSIADEVSFESPRTSSHCHSNRYRGEI
>AtPAP27
MARNFLLVLLWFIVQVSSSHENGRGDQALSQIDIYAINLAQHHSAFIHVSPLVLGSQGQDTEWVN
VVISNPEPSSDDWVGVFSPAKFDSSSCAPTDDKEIAPFICSAPVKYMYAKSSPDYMKTGNAVLKF
MLINQRADFSFALFTGGLSNPTLVSVSNHVSFINPKAPVYPRLALGKKWDEMTVTWTSGYNIGE
AVPFVEWSRKGTRSRRSPAGTLTFTRNSMCGAPARTVGWRDPGFIHTASLKDLWPNLKYTYRM
GHELMNGSIVWSKNFTFKSSPYPGQDSLQRVIIFGDMGKGERDGSNEYNDYQPGSLNTTDQLIK
DLKNIDIVFHIGDITYANGYISQWDQFTAQVEPIASTVPYMVASGNHERDWPDSGSFYGGKDSGG
ECGVPAETMFDFPAENKAKFWYSADYGMFRFCVADTEHDWREGSEQYQFIERCLASVDRRAQP
WLIFIAHRVLGYSTNDWYGQEGSFEEPMGRESLQKLWQKYKVDIAFYGHVHNYERTCPIYQNQ
CMDNEKSHYSGAFKGTIHVVVGGAGSHLSSFSSLKPKWSIFRDYDYGFVKLTAFDHSSLLFEYK
KSSNGAVHDSFTIFREYRDVLACVRDSCEPTTLAS
>AtPAP29
MADNRRRRSLFDFLLFSVFLGLACLCLSPIPATAQRRKLRFSVNGEFKILQVADMHFANGAKTQC
QNVLPSQRAHCSDLNTTIFMSRVIAAEKPDLIVFTGDNIFGFDVKDALKSINAAFAPAIASKIPWV
AILGNHDQESTFTRQQVMNHIVKLPNTLSQVNPPEAAHYIDGFGNYNLQIHGAADSKLQNKSVL
NLYFLDSGDYSSVPYMEGYDWIKTSQQFWFDRTSKRLKREYNAKPNPQEGIAPGLAYFHIPLPEF
LSFDSKNATKGVRQEGTSAASTNSGFFTTLIARGDVKSVFVGHDHVNDFCGELKGLNLCYGGGF
GYHAYGKAGWERRARVVVVDLNKKRKGKWGAVKSIKTWKRLDDKHLSVIDSQVLWNNSAN
KLVVR
>AtPAP23
MTLLIMITLTSISLLLAAAETIPTTLDGPFKPLTRRFEPSLRRGSDDLPMDHPRLRKRNVSSDFPEQI
ALALSTPTSMWVSWVTGDAIVGKDVKPLDPSSIASEVWYGKEKGNYMLKKKGNATVYSQLYPS
DGLLNYTSGIIHHVLIDGLEPETRYYYRCGDSSVPAMSEEISFETLPLPSKDAYPHRIAFVGDLGLT
SNTTTTIDHLMENDPSLVIIVGDLTYANQYRTIGGKGVPCFSCSFPDAPIRETYQPRWDAWGRFM
EPLTSKVPTMVIEGNHEIEPQASGITFKSYSERFAVPASESGSNSNLYYSFDAGGVHFVMLGAYV
DYNNTGLQYAWLKEDLSKVDRAVTPWLVATMHPPWYNSYSSHYQEFECMRQEMEELLYQYR
VDIVFAGHVHAYERMNRIYNYTLDPCGPVYITIGDGGNIEKVDVDFADDPGKCHSSYDLFFFNSL
NLSN
>AtPAP16
MKKPSLFQIIIIVLSIPTTTGRTVGNLRVREGSPFKIAIFADLHFGEDTWTDWGPGQDVNSVNVMS
AVLDAETPDFVVYLGDVVTANNIAIQNASLFWDKAISPTRDRGIPWATLFGNHDDASFVWPLDW
LSSSGIPPLRCPAASDDDGCTFRGTTRVELIQEEIKSSNALSYSMISPKELWPSVSNYVLLVESSDH
SKPPVALLYFLDSGGGSYPEVISNAQVEWFKTKSNTLNPYLRIPELIFWHIPSKAYKKVAPRLWIT

KPCVGSINKEKVVAQEAENGMMRVLENRSSVKAVFVGHNHGLDWCCPYKDKLWLCFARHTG
YGGYGNWPRGSRILEISEMPFRIKTWIRMEDGSVHSEVNLTYD
>AtPAP2
MIVNFSFFLLLFVSVFVSSADSKATISISPNALNRSGDSVVIQWSGVDSPSDLDWLGLYSPPESPND
HFIGYKFLNESSTWKDGFGSISLPLTNLRSNYTFRIFRWSESEIDPKHKDHDQNPLPGTKHLLAESE
QLTFGSGVGMPEQIHLSFTNMVNTMRVMFVAGDGEERFVRYGESKDLLGNSAAARGMRYERE
HMCDSPANSTIGWRDPGWIFDTVMKNLNDGVRYYYQVGSDSKGWSEIHSYIARDVTAEETVAF
MFGDMGCATPYTTFIRTQDESISTVKWILRDIEALGDKPAMISHIGDISYARGYSWVWDEFFAQV
EPIASTVPYHVCIGNHEYDFSTQPWKPDWAASIYGNDGGGECGVPYSLKFNMPGNSSESTGMKA
PPTRNLYYSYDMGTVHFVYISTETNFLKGGSQYEFIKRDLESVDRKKTPFVVVQGHRPMYTTSN
EVRDTMIRQKMVEHLEPLFVKNNVTLALWGHVHRYERFCPISNNTCGTQWQGNPVHLVIGMAG
QDWQPIWQPRPNHPDLPIFPQPEQSMYRTGEFGYTRLVANKEKLTVSFVGNHDGEVHDTVEML
ASGVVISGSKESTKIPNLKTVPASATLMGKSESNALWYAKGAGLMVVGVLLGFIIGFFTRGKKSS
SGNRWIPVKNEET
>AtPAP5
MSLETFPPPAGYNAPEQVHITQGDHNGRGMIISWVTSLNEDGSNVVTYWIASSDGSDNKSVIATT
SSYRYFDYTSGYLHHAIIKELEYKTKYFYELGTGRSTRQFNLTPPKVGPDVPYTFGVIGDLGQTY
ASNQTLYNYMSNPKGQAVLFAGDLSYADDHPNHDQSKWDSYGRFVEPSAAYQPWIWAAGNHE
IDYAQSIGETQPFKPYKNRYHVPYRASQNKYTPQNSWLQDEFKKVNRSETPWLIVLVHAPWYNS
NNYHYMEGESMRVTFEPWFVENKVDIVFAGHVHAYERSERVSNIQYNITDGMSTPVKDQNAPV
YITIGDGGNIEGIANIFTDPQPSYSAFREASFGHALLEIKNRTHAHYTWHRNKEDEAVIADSIWLK
NRYYLPEEETI
>OsPAP1a
MAAAAAAVLHALVALSLAGAVAAAGRGGEQPLSRIGIHRTTFAIQPGASVDASPLLLGLEGQDR
EWVTLTYNNPKPSKDDWIGVFSPANFSDSTCPSESQWVEPPLLCTAPIKFIFANYKNLDYEKTGK
GSMKLQLINQREDFSFALFSGGLSNPKLIAHSKRVTFTNPKAPVYPRLAQGKSWNEMTVTWTSG
YGTNEATPFVKWGLQGQIQSLSPAGTLTFSRSTMCGPPARTVGWRDPGFIHTSFLKDLWPNFKY
TYRIGHRLSDGSIIWGHEYSFQAPPYPGEDSLQRVVIFGDMGKAEADGSNEFNDFEPGSLNTTYQ
LIKDLKNIDMVIHIGDICYANGYLSQWDQFTAQVEPIASSVPYMVGSGNHERDWPGSGSFYGNL
DSGGECGVPAQNMFYVPAENREQFWYSIDYGMFRFCIANTELDWRPGTEQYKFIEHCFSSVDRQ
KQPWLIFLAHRVLGYSSASFYVEEGTTEEPMGRESLQPLWQKYKVDIAMYGHVHGYERTCPVY
ENVCVAKAASHYSGAFTATTHVVVGGGGASLADYAGVRARWSHVQDRDYGFAKLTAFNHTA
LLFEYVRSRDGSVHDSFTVSRDYRDILACGVDNCPTTTLAS
>OsPAP1d
MIRLWVVATWLIVCAAAHPGEQPLSRIAVERTVLAVNESAHVKASPWVLGLKGQNSEWVEVEF
FHPSPSNDDWIGVFSPANFSAAICEPENKRQRPPVLCTAPIKYQFANFNNDGYNKSGKGYLKLQLI
NQREDFSFALFSGGLLKPKLIAVSNKVAFANPKAPVYPRLAQGKSWNEMTVTWTSGYDIKEAVP
FVEWGAKGGRSFLSPAGTLTFDRNSMCGAPARTVGWRHPGYIHTSYLKDLWPDSLYTYRLGHR
LPNSTLIWSKSYSFKASPYPGQDSLQRVVIFGDMGKAEADGSNEFNDFQPGSLNTTYQIIRDLENI
DMVVHIGDICYANGYLSQWDQFTAQIEPIASTVPYMIGSGNHERDWPGTGSFYGNLDSGGECGV
PAQTVFYTPAENRAKFWYATDYGMFRFCIAHTEEDWRPGTEQYKFIEQCLSSVDRQKQPWLIFL
AHRVLGYSSCSYYEEQGTFGEPMGRDTIEELLQKYRVDLAFYGHVHSYERTCPVYQGQCVVNA
SDHYNGPFKATTHVVVGGGGASLSEFTTSKIKWSHYTDFDFGFVKLTAFNHSSMLFEYKKSRDG
NVYDHFTISRDYRDILACSVDNCPRTTLAT
>OsPAP1c
MIRLWVVVVTWLVLWAAAAVHPGEQPLSRIAVERMVLAVNESAHVRASPLVLGLKGETNEWVE
VEFFNPNPSNTDWVGVFSPADFSSAICEAYGVPQYYPMLCTAPIKYQYANFNNNGYSKSGKGKL

KLQLINQREDFSFALFSGGLENPKLVAVSNKIAFANPKAPVYPRLAQGKSWNEMTVTWTSGYDF
KEAVPFVEWGAKGGQRVLSPAGTLTFDRNSMCGAPARTVGWRHPGYIHTSYLKELWPDSLYTY
RLGHRLPNGTHIWSKSYSFKASPYPGQDSVQRVVIFGDMGKAEADGSNEFNDFQPGSLNTTYQII
RDLKNIDMVVHIGDICYANGYLSQWDQFTAQIEPIASTVPYMIGMGNHERDWPGTGSFYGNLDS
GGECGVPAQTVFYTPAENRAKLWYATDYGMFRFCIANTEEDWRPGTEQYKFIEQCLSSVDRQK
QPWLIFLAHRVLGYSSCTFYEEEGTFEEPMGRESLQELWQKYKVDLAFYGHVHNYERTCPVYQ
NKCVVSGSDHYSGPFTATTHVVVGGAGAGTSDSEFTTSNIKWSYYRDFDYGFVKLTALNHSSLL
FEYKKSSDGNVYDHFTISRDYRDILACSIDNCPRTTLAT
>OsPAP27a
MAMPLGGILLLFLVLLAAAAAGGGGGVWAFSSSSSSSSYSRIGEQPLSLIGIHRATVGIDAAASVQ
ASPRLLGVKGEDTAWVTVDFAAPHASDGDWIGVFSPSNFNASTCPGPSGSDSGPVICSAPIKYQL
ANYSSDYGKTGKGTLKFQLINQRQDFSFALFTGGLSNPKLIAVSNKIAFANPKAPVYPRLAQGKS
WNEMTVTWTSGYDIKEAYPFVEWGMKWSPPTRTAAGTVTFDRESLCGEPARTVGWRDPGFIHT
AFLTDLWPNKEYYYKIGHMLPDGKIVWGKFYSFKAPPFPGQKSLQRVVIFGDMGKAERDGSNE
YSNYQPGSLNTTDTLIKDLDNIDIVFHIGDITYANGYISQWDQFTQQVEPITARVPYMIASGNHER
DWPNSGSFFNGTDSGGECGVLAETMYYTPTENRANYWYKTDYGMFRFCVADSEHDWREGTEQ
YAFIESCLATVDRKKQPWLVFIAHRVLGYSSGFFYGAGGAFAEPTARQSLQRLWQRHRVDLAFY
GHVHNYERTCPVYDGRCASPERSRYSGAVGGTIHAVVGGGGSHLSNFTAEAPPWSVYREMDYG
FVKLTAFNYTSLLYEYRRSSDGEVHDSFTVHREYRDVLACVADSCPPTIPPAT
>OsPAP1b
MRFLIMAAIRWVVLAYIVVIGCATIARGDEQPLSRIAIERATVAAVDSASVKAQPTVLGLKFQYA
NFNNADYNRSGKGLLRLQLINQREDFSFALFSGGLSAPKLIAISNKVSFQNPKAPVYPRLAQGKS
WNEMTVTWTSGYSIKEAIPFVEWGHKGGNQMLSPAGTLTFSRNSMCGSPARTVGWRDPGYIHT
SFLKELWPDSLYTYRLGHRLLDGTHIWSKSYSFRASPYPGQDSVQRVVIFGDMGKAEIDGSDEY
GNYEQASLYTTNQLIKELDSIDMVIHIGDLSYANGYLSQWDQFTQQIEPIASTVPYMIGSGNHERD
WPGSGSFYGHNDSGGECGVPTQTMFYVPAENRAKLWYSTDYGMFRFCIADTEQDWRPGTEQY
KFIEQCLSSVDRSKQPWLIFLAHRVLGYSSASWYEIMMGSYGEPMGRDGLEELWQKYKVDLAV
FGHIHSYERTCPIYQNRCVQDGSNLYTGQFNATTHVIVGGGGAMLSPFRATVPYWSFFRDYDFG
FSKLTALNHSTLLFEYKKSRDGKVYDHFTISRDYRDIMACSIDNCPRTTLAV
>OsPAP27b
MVSRKRGGGGGVAMAVAMLLAAASASRPSSSLEGFQPLSKIAVHKATVDLHGSAFVSATPALL
GDQGEDTEWVTVKYGWANPSADDWIAVFSPADFISGSCPNPSRYPDEPLLCTAPIKYQFANYSA
NYVVYWGKGSIRFQLINQRYDFSFALFTGGLENPKLVAVSEAISFKNPKAPVYPRLAQGKSYDEM
TVTWTSGYDISEAYPFVEWGMVVAGAAAPTRTAAGTLTFNRGSMCGEPARTVGWRDPGFIHTA
FLRDLWPNKEYYYKIGHELSDGSIVWGKQYTFRAPPFPGQNSLQRIIVFGDMGKAERDGSNEFA
NYQPGSLNTTDRLVEDLDNYDIVFHIGDLPYANGYISQWDQFTAQVAPITAKKPYMIASGNHER
DWPNTGGFFDVKDSGGECGVPAETMYYYPAENRANFWYKVDYGMFRFCIADSEHDWREGTD
QYKFIEQCLSTVDRKHQPWLIFAAHRVLGYSSNWWYADQGSFEEPEGRESLQRLWQRHRVDVA
FFGHVHNYERTCPMYQSQCVSGERRRYSGTMNGTIFVVAGGGGSHLSDYTSAIPKWSVFRDRDF
GFVKLTAFNHSSLLFEYKKSSDGKVYDSFTVERDYRDVLSCVHDSCLPTTLAS
>OsPAP27c
MGAARGVLQAALLLAAAAAAFLLVSPAAAAAVNSTSATLDNIQPLSTLNMAAARVAMDAGSAI
RASPELLGTNGEDSAWVTVNFTTPAPTDGHWIALFSPADFDLIMGGKQSSSRINAAGEDEAPAGL
PIAPIKYKFANISPSFMSSGSGDTSFLLINQRYDYAFGLFSGGKDNPKLVAVSNKISFANPKAPVFP
RLSQGKGWNEMAVTWTSGYNVDEAYPFVEWTMNEKENARARRSPADTLTFTRNHLCGKPAN
AEGYRDPGFIHTAFLKNLWPNREYSYQIGHELLDGTIVWGKSSTFRASPSPGQASLQRIVIFGDM
GLGQSDGSNELAGFQPGAQVTTERLIKDLPNYDAVFHIGDLSYANGFLAQWDQFTAQISPVASR

VPYMVASGNHERTSRDTGGFYGGDDSHGECGVPAETYFRAPAAANRGKPWYAADHGMFRFCV
GDTEHDWRPGTAQHAFLDGCFAAADRKHQPWLVFAAHRPLGYSSNEYYAREGSFSEPMGRTL
QPLWQKHRVDLAVYGHVHNYERTCPVYENTCTAAPAAAGGGGNGSSPAAAYTGALGGTIHVV
AGTGGARLRGYAGGEWPQWSAARSESYGYVKLTARDHSRLELEFIRSDDGEVLDAFSITRGYK
DVLACAVDACDPHTLAN
>OsPAP9b
MARLLLLLVLIGGICAAAAASSPLLPAAETMRVSFAGKSEFRTVNRKPLGTCLDPSPYLEISVSTG
GAPLPDEAFLNVTVSGVRRPDRSHWLAMITPSNSSVLGCPLNGVNYIETGDLASLPLLCHYPVKA
QYLTSDPGYLGCKASACQKRRASGTCKVRTCAATLAFHVINFRTDVEFVLFSGGFATPCVLKRS
GALPFANPAKPLHGHLSSVDSKATSMRLTWVSGDARPQQVQYGTGKTATSVATTFTHKDMCSI
AVLPSPAKDFGWHDPGYIHSALMTGLQPSQSYNYRYGSDSVGWSNTTEFRTPPAAGSGELSFVIF
GDMGKAPLDPSVEHYIQPGSTSVAKAVAAEMQTGKVDSIFHIGDISYATGFLVEWDFFLHLITPL
ASQVSYMTAIGNHERDYAGSGSVYPTPDSGGECGVPYESYFPMPASGRDKPWYSIEQGSVHFVV
MSTEHEWSEKSDQYNWMEMDLSSVDRSRTPWVIFIGHRPMYSSSSGIPPSVDPNFVSSVEPLLLN
HKVDLVFFGHVHNYERTCAVYQGNCKGMPKKDAKGVDTYDNSNYAAPVHAVVGAGGFNLDG
FPKIGLHSWSLSRISEFGYARVHATKTDMLVQFVNSNTSAVQDQFRIVKGAR
>OsPAP9a
MGWRFALLLLHVLLCLVNGVSCGRTSSYVRTEYPSTDIPLESEWFAVPNGYNAPQQVHITQGDY
NGKAVIVSWVTVAEPGTSEVLYGKNEHQYDQRVEGTVTNYTFYDYKSGYIHHCLVDGLEYNTK
YYYKIGSGDSAREFWFETPPAIDPDASYTFGIIGDLGQTFNSLSTLQHYEKSEGQTVLFVGDLSYA
DRYQHNDGVRWDSWGRLVERSTAYQPWIWSAGNHEIEYRPDLGETSTFKPYLHRCHTPYLASK
SSSPMWYAVRRASAHIIVLSSYSPFVKYTPQWTWLKYELKHVDREKTPWLIVLMHSPMYNSNE
AHYMEGESMRAAFEKWFVKYKVDLVFAGHVHAYERSYRISNINYNITSGNRYPVPDKSAPVYIT
VGDGGNQEGLASRFSDPQPDYSAFREASYGHSILQLKNRTHAIYQWNRNDDGKHVPADNVVFH
NQYWASNTRRRRLKKKHFHLDQIEDLISVFMLLFLLFLLAAGEAAAAAAATTLTATPAKLTQSD
REITIRWSGLPDPDGLDYVGIYSPPTSSDRDFLGYLFLNGSATWRTGTGELTLPRLPNLRAPYQFR
LFRWPAREYSYHHIDHDGNPLPHGRHRVAASGEVAFDSPSRPDQVHLSFADGVDEMRVMFVCG
DGGRRVVRYGPAKEEGEGWKEVAAEVRTYEQKHMCDSPANSSVGWRDPGFVFDGLMKGLEP
GRRYFYKVGSNSSGWSDTYSFISRDNEANETIAFLFGDMGTYIPYNTYVRTQDESLSTVKWILRD
IQALGDKPAFISHIGDISYARGYAWVWDHFFNQIEPIAANTPYHVCIGNHEYDWPLQPWKPWWA
TGIYGTDGGGECGIPYSVKFRMPGNSFVPTGNGAPDTRNLYYSFDSGVVHFVYMSTETNFVQGS
DQYNFIKADLEKVNRSRTPFIVFQGHRPMYTSSNEARDFAHRQQMLQNLEPLLVTYKVTLALWG
HVHRYERFCPMKNFQCVNMSSSFVYPGAPVHLVIGMGGQDYQPFWQPRKDHPDVPVYPQPERS
MYRGGEFGYTKLVATKEKLTLTYIGNHDGQVHDMVEIFSGQVSNNNGVPEVIDDTKLSTGVSTK
LKIPLFSLEIVGSVMFALVLGFSLGFLIRRKKEAAQWTPVKNEET
>OsPAP26
MGWRFALLLLHVLLCLVNGVSCGRTSSYVRTEYPSTDIPLESEWFAVPNGYNAPQQVHITQGDY
NGKAVIVSWVTVAEPGTSEVLYGKNEHQYDQRVEGTVTNYTFYDYKSGYIHHCLVDGLEYNTK
YYYKIGSGDSAREFWFETPPAIDPDASYTFGIIGDLGQTFNSLSTLQHYEKSEGQTVLFVGDLSYA
DRYQHNDGVRWDSWGRLVERSTAYQPWIWSAGNHEIEYRPDLGETSTFKPYLHRCHTPYLASK
SSSPMWYAVRRASAHIIVLSSYSPFVKYTPQWTWLKYELKHVDREKTPWLIVLMHSPMYNSNE
AHYMEGESMRAAFEKWFVKYKVDLVFAGHVHAYERSYRISNINYNITSGNRYPVPDKSAPVYIT
VGDGGNQEGLASRFSDPQPDYSAFREASYGHSILQLKNRTHAIYQWNRNDDGKHVPADNVVFH
NQYWASNTRRRRLKKKHFHLDQIEDLISVF
>OsPAP10a
MVDRIGAAWWCACAVGMLVVGACLAGETSEYRRQLGSAVDMPLDADVFRAPPGRNAPQQVH
ITQGNHDGTAMIISWVTTIEPGSSTVLYGTSEDNLNFSADGKHTQYTFYNYTSGYIHHCTIKKLEF

DTKYYYAVGIGQTVRKFWFRTPPKSGPDVPYTFGLIGDLGQSYDSNITLAHYESNSKAQAVLFV
GDLCYADNYPYHDNVRWDTWARFVERNVAYQPWIWTAGNHEIDFAPELGETKPFKPYSYRYP
TPYKASGSTAPFWYSVKRASAYIIVLASYSSYGKYTPQYKWLEAEFPKVNRSETPWLIVLLHAP
WYNSYNYHYMEGESMRVMYEPWFVKYKVDLVFAGHVHAYERTHRISNVAYNIVNGQCTPVH
DQSAPVYITIGDGGNQEGLATNMTAPQPGYSAFRESSFGHAILDIKNRTHAYYTWHRNQDGNAV
AADSMWFTNRYWQPTDESLDDSQ
>OsPAP18
MEERAGARRRPPMAVPPLLLFLLLLSSFSSCAAAASGAPVGEDYVRPPAAARRCGLHHRKALLS
LFPWSKKKDSSSASDPQQVHISLAGEKHMRVTFVTDDNSVPSVVDYGTEAGTYTSTSQGESTSY
SYLMYSSGKIHHVVIGPLNDNTVYYYRCGGHGPEFQFKTPPSQFPLSLAVVGDLGQTSWTTSTLN
HIKQCAHDMLLLPGDLSYADYMQHLWDSFGTLVEPLASTRPWMVTEGNHEKERIPFFKSGFQSY
NARWKMPYEESESTSNLYYSFKVAGVHAIMLGSYTDYDESSDQYAWLKADLAKVDRKRTPWL
IVLLHAPWYNSNWAHQGEGDSMMAAMEPLLYAAHVDMVIAGHVHAYERAERVYKGGLDPCG
AVHITIGDGGNREGLAHRYRNPKPAWSVFREASFGHGELKIVNATHAHWTWHRNDDEEPVRTD
DVWITSLAGSGCIQDGSHEYRKILMSP
>OsPAP10d
MGFGFTVRSFWFTTPPRPGPDVAFRLGLIGDIGQTFDSNATLTHYEASGGDAVLFMGDLSYADK
YPLHDNNRWDTWGRFSERSVAYQPWIWVAGNHEIDYAPELGETKPFKPFTHRYPTPHLASASPE
PYWYSVKLASVHIIVLSSYSAFAKYTPQWKWLEAELGRVNRSETPWLIMASHSPWYNSNNFHY
MEGESMRAQLEKMAVDARVDLVFAGHVHAYERSFRVSNIRYNITDGLCTPVRDRRAPVYVTIG
DGGNIEGLADEMTWPQPPYSAFREDSFGHAVLDIKNRTHAYYAWYRNDDGAKVAADAVWFTN
RFHMPNHDDSTPTPTKRHYYG
>OsPAP20a
MAMAMTNTALAFFLLVAAASLLSLPPPSLAVTSPYVRPKPRATLSLLKDDDDGRKPEQVHISAV
GSDKMRVTWITGGDAPATVEYGTTSGQYPFSATGSTNTYSYVLYHSGNIHDVVIGPLQPSTTYFY
RCSNDTSRELSFRTPPASLPFKFVVAGDLGQTGWTESTLRHIGGDDYDMLLLPGDLSYADLYQPR
WDTYGRLVEPLASARPWMVTQGNHEVERIPLVEPHAFKAYNARWRMPFDAGASPSGSNLYYSF
DVAGGAVHVIMLGSYADYAAGSAQHRWLRRDLAAVDRARAAFVVALVHAPWYNSNEAHRGE
GDAMRAAMEELLRGARVDAVFAGHVHAYERFARVYGGKEDPCGAVHVTIGDGGNREGLAGS
YVDPQPAASAFREASFGHGRLEVVNATHALWTWHRNDDDEAVVADQAWITSLASNPACNK
>OsPAP10c
MGMLRWGAHLLLLLLAAATWTCAGAGAGVTSEYRRKLEATVDMPLDADVFRVPPGYNAPQQ
VHITLGDQTGTAMTVSWVTANELGSNTVRYGSSPEKLDRAAEGSHTRYDYFNYTSGFIHHCTLT
GLTHATKYYYAMGFDHTVRTFSFTTPPKPAPDAPFKFGLIGDLGQTFDSNSTLAHYEANGGDAV
LFVGDLSYADNYPLHDNNRWDTWARFVERSVAYQPWIWTAGNHELDYAPELGETVPFKPFTHR
YPTPYRAAGSTEPFWYSVKIASAHVIVLASYSAYGKYTPQWTWLQEELATRVDRKLTPWLIVLM
HSPWYNSNNYHYMEGETMRVQFERWLVDAKVDVVLAGHVHSYERSRRFANIDYNIVNGKATP
AANVDAPVYITIGDGGNIEGIANNFTVPQPAYSAFREASFGHATLEIKNRTHAHYAWHRNHDGA
KAVADAVWLTNRYWMPTNDDV

>OsPAP21b
MTCNYALLSQVVSSSGLSPGWPPMMMATAAMAASSCDRGDTRKKLQITVVFLVRTLLLACIIAR
GVLALIRVAFRVAVVAPARSLVAVAGAAFSAVNARCAWCLEQAALGRSCTGTVLGDAVVGAM
ASSWRLLLQGITSLVFLCARGADEYVRPPPSPLVLTAHGKPASHPQQVHISMVGEKNMRISWVT
DDLNAPSVVEYGTSPGKYTASATGDHTTYRYFLYKSGAIHHATIGPLEASTTYHYRCGKAGDEF
TLRTPPARLPVEFVVVGDLGQTKWTASTLSHIGGGGGDYDVLLLPGDLSYADTQQPLWDTFGRL

VQPLASARPWMVTEGNHEIEALPVVGIAPFAAYNARWRMPREESGSPSNLYYSFDAAGGAAHV
VMLGSYAEFEEGSPQRAWLERDLAGVDRRRTPWLLALVHAPWYNTNEAHQGEGERMRRAME
SLLYEARVDVVFAGHVHAYERFTRIYDNEADSRGPMYITIGDGGNREGLALKFIKGHKSAHLSEF
REASFGHGRLRVLNETSAVWTWHRNDDQFATVRDEVWLHSLAAGEPAATVASAAGGGGGHPA
DEL
>OsPAP21a
MTRRADDLLVAGTLVISIVFFRCAAAVAATEYVRPPPGRVIFTEHTKPASHPQQVHVSLVGANH
MRVSWITEDKHVKSVVEYGKVSGNYTASATGEHTSYRYFLYSSGKIHHVKIGPLDPGTVYYYRC
GMAGDEFGLRTPPAALPVELAVAGDLGQTEWTASTLSHVGRSDYDVLLVPGDLSYADAQQPL
WDSFGRFVQKYASRRPWMVTEGNHEVEAAMALPGWPRPFTAYAARWRMPYEESGSGTSLYYS
FDAAGGAVHVVMLGSYADFNSSSEQYRWLARDLAAVDRGATPWVVVLLHAPWYNTNAAHEG
EGEAMRKAMERLLYEARVDIVFAGHVHAYERFTRVYNNEANPCGPVHITIGDGGNREGLAFDF
RKNHKLAPLSLMREASFGHGRLSVVNATAARWTWHRNDDADSTVRDEIWLESLAANGACQQS
SSAAAAADSQNDEL
>OsPAP23
MAAPAAACDLRFLLVGLLLVVVVGSRLVRPPDGGGIPTTLDGPFEPATRAFDRALRQGSDDVPL
TDPRLAPRARPPAPEQIALAASSDATSVWVSWVTGEAQVGSHLTPLDPSTVRSEVWYSERPSPTA
AAAGDVSGHYPHVARGKAEVYSQLYPYPGLLNYTSGAIHHVRLRGLRPATRYYYRCGDSSVRG
GAGLSGELSFETLPSSAAAAYPRRVAVVGDLGLTGNSTSTVEHLARNDPSLVVVVGDMTYANQ
YRTTGGRGVPCFSCSFPDAPLRESYQPRWDGWGRFMEPLTSRIPMMVIEGNHEIEPQGQGGAVT
FASYLARFAVPSEESGSNTKFYYSFNAGGIHFIMLGAYVDYNRTGAQYSWLEKDLRKIDRRVTP
WVVAAWHPPWYNSYSSHYQEFECMRQAMEGLLYQHGVDIVFSGHVHAYERMNRVFNYTLDP
CGPVYITIGDGGNIEKIDIDHADDPGKCPGPGDNHPEFGGVCHLNFTSGPAKGKFCWEKQPEWSA
FRESSFGHGILEVVNSTYALWTWHRNQDAYGEDSVGDQIYIVRQPDKCLLQTTSASSENNCPSEG
CPSLVSNSGYGAQKDIIRSGHLIWNASLVIWMILISTVFMKGNLCSRF
>OsPAP20b
MAMASVAALRLVVLLAAAVPLLPPPAASLAVTSTYVRPTARATLSVLHDGDGRTPQQVHISAV
GSDKMRVTWITDDDAPATVEYGTVSGEYPFSAAGNTTTYSYVLYHSGNIHDVVIGPLKPSTTYF
YRCSNDTSRELSFRTPPASLPFKFVVVGDLGQTGWTASTLRHVAADVYDMLLLPGDLSYADFYQ
PRWDTFGRLVEPLASARPWMVTEGNHEVERIPVIHPRPFTAYDARWRMPHDAGASPSGSNLYYS
FDVAGGAVHVVMLGSYAGYAAGSAQHRWLRRDLAGVDRAKTAFVVALVHAPWYNSNRAHR
GEGDAMRAAMEELLYGARVDAVFAGHVHAYERFARVYGGGEDACGPVHVTVGDGGNREGLA
TRYVDPQPAASAFREASFGHGRLEVVNATHALWTWRRNDDDEAVVADEVWITSLASNPACNK
KYSISLY
>OsPAP15
MRMRVSLLLLAAAAVAAAAEAAPSSTLAGPTRPVTVPPRDRGHAVDLPDTDPRVQRRVKGWA
PEQIAVALSAAPSSAWVSWVTGDFQMGAAVEPLDPTAVASVVRYGLAADSLVRRATGDALVYS
QLYPFDGLLNYTSAIIHHVRLQGLEPGTEYFYQCGDPAIPAAMSDIHAFRTMPAVGPRSYPGKIAI
VGDLGLTYNTTSTVEHMVSNQPDLVLLLGDVSYANLYLTNGTGTDCYSCSFANSTPIHETYQPR
WDYWGRYMEPVTSRIPMMVVEGNHEIEEQIDNKTFASYSSRFSFPSTESGSFSPFYYSFDAGGIHF
IMLAAYADYSKSGKQYKWLEKDLAKVDRSVTPWVIAGWHAPWYSTFKAHYREAECMRVAME
ELLYSYAVDVVFTGHVHAYERSNRVFNYTLDPCGPVHISVGDGGNREKMATSYADEPGRCPDP
LSTPDPFMGGGFCGFNFTSGPAAGSFCWDRQPDYSAYRESSFGHGILEVKNETHALWRWHRNQ
DLYGSVGDEIYIVREPDNLQPVQLGGGGGGRRRGGGGAGMVSLAAYSPCSTVAGVPKNKGNGA
ASSTHKESIMRHVVVQCATSWDTPRTSTTNGSHAEPSAVVKAGTAPLIQALKSTANQDVSCFHFP
GHNRGKASPPSLSELIGSRTFLHDLPELPELDDLFSPKGVILDAQKRAAELFGSFKTWFLVNGSTC
GIQASVMATCSPGDYLIIPRNCHISVISALVLSGAVPKYIVPEYNSGWDIAGGITPSQVDKVVKELE

EDRKKVGAVLVTSPTYHGICSNIQGIVNVCHLQGIPVIVDEAHGAHFRFHRNFPSSATEQGADLV
VQSTHKVLCSLTQSSMLHMAGDLVDADKVSQCLQLLQSSSPSYLLLSSLDAARAQLSENAESFD
EPVSMALETKHQLRIIPGISVLDLSSFLSDFPAIDPLRITLSASDLQLSGYEADDFLAEEHQIVSELV
GTQAVTFAVNLGTRRHDVQRLVHSVKHLSEKYFSENGSSSRKENPASSPLDKFSIKLTPREAFFL
KKRRVSIEDSLGEICGELICPYPPGIPVLIPGEIVTQDSLSYLMDVRDNGIAISGAADGELKSIMVCN
V
>OsPAP3a
MDAPVITLLVFLVAVAAATAAAEMPRMEHPRKGDGSLSLLAVGDWGRRGAYNQSMVAAQMG
IVGEKMDIDFVISTGDNFYKNGLTGVDDKAFEESFSDIYTAKSLHKPWYTVLGNHDYRGDALAQ
LSPVLRKVDSRWICIKSFVVSAEIADFFFVDTTPFVLKYWTDPKNSKYDWRGVSPRETYIANVLK
DLEDALEQSKAPWKIVVGHHAIRSVSQHGDTKELLEHLLPILKAHGVDLYLNGHDHCLEHISSRD
SKIQYLTSGPGRRRGAGCRRRTRTRWSSSTTGRDSCRCG
>OsPAP3c
MARRSSSRACAAMATAAVFALLAATATASGLVRVEHPAKSDGSLSLLVVGDWGRKGTYNQSR
VAEQMGKVGEKLNIDFVISTGDNFYEDGLTGVDDQAFEESFTDIYTAKSLQKPWYLVLGNHDYR
GDVLAQLSPVLRKIDQRFICMRSFIVNAEIVDFFFIDTTPFQLKYWTRPKDHHYDWRGVAPRQKY
ITNLLKDMDEAMKKSTAKWKIAVGHHTIRSVSDHGDTKELLQLLLPVLKVNGIDFYINGHDHCL
EHISSRDSPIQYFTSGGGSKAWRGVLQPNSDKLQFFYDGQGFMSLQINQDQADFIFYDVSGNILY
KWSKSKANYLQPSTYITEA
>OsPAP21c
MANSGKKLMDGGGVAFQTLPRAAGDDNADDDYVRPPPRPLVSTVHDKPATHPQQVHISVVGA
NRMRICWVTDDDDGRSSPPSVVEYGTSPGEYTASATGDHATYSYSDYKSGAIHHVTIGPLEPATT
YYYRCGAGEEEELSLRTPPAKLPVEFVVIGDVGQTEWTAATLSHIGEKDYDVALVAGDLSYADG
KQPLWDSFGRLVQPLASARPWMVTEGNHEKEKTPPPPPVAGAGAGVRLSPSRFAAYNARWRMP
REESGSPSSLYYSFDAAGGAAHVVMLGSIQLLLIDVINRGIMIDYKTRIYDNEANSQGPMYITIGD
GGNVDGHSDKFIEDHELAHLSEFREMSFGHGRLRIVSETKAIWTWHRNDDQHATVRDVVVLES
MAGAKTN
>OsPAP10b
MVMEAACVLAVVVVVMAFLSPAARGGVTSTYRRSLQALPDMPIDADVFRPPPGFNAPEQVHIT
LGDQTGRAMTVSWVTPKLPDSNVVRYGLRADNLTHTANGTFRRYSFGRKYRSGFIHHATLTGL
DYGTKYHYAVGSGDTASARSFSFTTPPKPGPDVPYKFGLIGDLGQTFHSNDTLSHYEACGGDAV
LFIGDLSYADNHPGHDNNRWDTWARFVERSVAYQPWIWTTGNHELDFAPELGETTPFKPFTNR
YPTPFGASGSTRPLWYSVRMASAHVIVLASYAAYGKYTPQWRWLEGELRRVDRAVTPWLIVCV
HSPWYSSNGYHYMEGESMRVEFERWLVDAKADVVLAGHVHSYERTRRVSNVAYDIANGMAT
PVFNRSAPVYINIGDGGNIEGLADDFRWPQPDYSVFREASFGHATLQIVNRTHAFYEWHRNSDG
VKVVADHAWFTNRYWFPTDTN
>OsPAP7a
MMMRCGWSAAAVVAVVVMVGVMVASPVAGELARVEHPTKEDGSLAVLVVGDWGRKGQYN
QTLVATQMGVIGEELAADFILSTGDNFYNDGLTGDNDTASFQESFTNIYTADSLQKPWYIVLGNH
DYTGDALAQQSPAIRAVDSRWTSINKSFIVDSDIAEFFLVDTVPFVQKYWNESKFDWRQVAPRD
TYLSTLLTDLGDAMSQSNATWKIVVGHHTISSGCEHGNTTDLVAMLLPVLKTYGADMYINGHD
HCLQRITSIDSPLEFITSGGGSRAWAGKFKQTSDKLEFIYDGQGFLSMQLTMAEASFAFYDVTGA
VLYSWQLAKSTSTN
>GmPAP1
MFCEISSLKLKPSCLLSTNKLIFLTVTEMGESKFVSLAFLLVCLVVQRVWSHGYHPLSKVAVHKA
TVSLLDLAYIKASPAVLGLQEQTAEWVTLEYSSPIPSIDDWIGVFSPANFSASTCPKENRRVYPPLL
CSAPIKYQYANYSSPLYKETGKGFLKLLLINQRSDFSFALFSGGLSNPKLVAVSDKIAFANPNAPL

YPRLALGKSWNEMTVTWTSGYGINDAEPFVQWGPKEGDRMHSPAETLTFTRDSMCGAPARTV
GWRDPGYIHTSHLKELWPNKIYEYRIGHKLNNVTYIWSGNYQFTAPPFPGQKSLQRVVIFGDMG
KGEVDGSNEYNNFQHGSINTTQQLIQDLENIDIVFHIGDICYANGYLPQWDQFTAQVEPIASAVPY
MIASGNHERDWPGTGSFYENMDSGGECGVLAQTMFYTPASNRAKLWYGDMTSYISSNYNQ*
>GmPAP2
MELKQQKLLLVLILTLLFATATPDSEYVRPLPRKTLTTIPWDSISKAHSSYPQQVHISLAGDKHMR
VTWITDDKHSPSYVEYGTLPGRYDSIAEGECTSYNYLLYSSGKIHHAVIGPLEDNTVYFYRCGGK
GAEFELKTPPAQFPITFAVAGDLGQTGWTKSTLAHIDQCKYDVYLLPGDLSYADCMQHLWDNF
GKLVEPFASTRPWMVTEGNHEEENILLLTDEFVSYNSRWKMPFEESGSTSNLYYSFEVAGVHVI
MLGSYADYDVYSEQYRWLKEDLSKVDRKRTPWLLVLFHVPWYNSNKAHQGAGDDMMAAME
PLLYAASVDLVIAGHVHAYERSKRLYNGRLDPCGAVHITIGDGGNREGLAHKYINPQPKWSEFR
EASFGHGELKIVNSTHAFWSWHRNDDDEPVKADDIWITSLVSSRCVDQKTHELRSTLTMPENSE
LRGLAKVM*
>GmPAP3
MAFPILLHTLCFLLFPQPLLSQENFVRQPAGQLIITPHHGSHSDPQQVHISLVGKEKMRVSWITED
KHTESVVEYGTKAGEYREKATGLHTSYQYFLYNSGKIHNVVIGPLQPGTTYFYRCGGSGPDFSF
KTPPPKFPIEFVIVGDLGQTEWTASTLKHVDSNDYDVFLLPGDLSYADSQQPLWDSFGRLVEPYA
SKRPWMVTEGNHEIESFPIIYPQGFQAYNARWPMPFQQSGSTSNLYYSFEVTATHFIMLGSYTDF
DAQSQQYTWLQSDLANIDRAKTPWVIVLLHAPWYNTNEAHQGEGESMRQAMEELLYEARVDL
VFAGHVHAYERFTRIYDNKADSCGPMYVTIGDGGNREGLALMFKNPPSPLSLYREPSFGHGRLRI
LNETHAHWSWHRNNDADAVVADGVWIESLSSSKACSKTPDQQDAANEEL*
>GmPAP4
VHISQVGQNKMRISWITDSPTPAKVSYGPSPSVNASSAIGTTSSYRYLVYESGEIHNVVIGPLNPNT
VYYYRLGDPPSSQTYNFKTPPSQLPIKFAVVGDLGQTDWTRSTLEHVNKSNYDMLLLPGDLSYA
DFIQDLWDSFGRLVEPLASQRPWMVTQGNHEVEMIPLIHTTPFTAYNARWLMPFQESGSNSNLY
YSFDVAGVHVIMLGSYTDFDSSSPQYKWLQNDLQKVNRRITPWVVVLIHAPWYNSNTAHQGEP
ESVNMKASMEDLLYQARVDVVFEGHVHAYERFTRVYKDKANNCAPMYITIGDGGNREGLATK
YINPKPTISIFREASFGHGTLEVFNVSHARWTWHKNDNDEAVISDFVWLTSFSSNPSCK*
>GmPAP5
MKTRKAILKFQLINQRADFSFGLFSGGLSNMTVTWTSGYDIDEGLLLNGNPPLLLVLYTLPHRPR
PNNCDNSRGTSGECLQERSPLDPMDDAEVEDVDVGGVFGGFEDGLGDSLKRMLHVAAFVVSGH
FAEFGWDARVALNIYGAFNGKEYIKAQKLRTLTFNRNSMCDQLIKDLDYYDIVFHIEDMPYANG
YTSQGDQFTAQVQEISSTVPYIIASGNHERDWPNTGSLFDTPDSGGECGVFAETMYYFPAENRAK
FWYKADYGLFRFCVADSEHDWREGSEQYKFIEHCLATIDRKHQPWLIFSAHRPLDYSSNDWYG
KEGSFEEPMGRESLQKLWQKYKVEIPFYGHVHNYERMCPIYQNQCVNQEKHQLLWHCERNNSC
GCWWWGKSLVRLHTNTPYLESLQGSLDYGFGKLTGIQSFISLV*
>GmPAP6
MKMKIEERVNMNLVLVVAWLVQLNIVYGFAHIHGFGEQPLSRIAIHKAVVSLHSSASITATPSLL
GTKGEDTQWVTVDIDYPDPSADDWVGVFSPAKFNASTCPPVNDPKEVIPYICSAPIKTGKASLKF
QLINQRADFSFALFSGGLLNPKLVAVSNFISFVNPKVPLYPRLAQGKSWDEMTVTWTSGYDINEA
TPFVEWGPKGKTQVQSPAGTLTFGRNSMCGSPARTVGWRDPGFIHTSFLKNLWPNLVYTYQLG
HLLSNGSYIWSKKYSFKSSPYPGQDSLQRVIIFGDMGKAERDGSNEYNAYQPGSLNTTDQLIKDL
ENIDIVFHIGDITYANGYLSQWDQFTAQVEPIASTVPYMIASGNHERDWPNTGSFYSTTDSGGEC
GVLAQNMFFVPAENRANFWYAMDYGMFRFCIADTEHDWREGSEQYKFIEHCLATVDRQKQPW
LIFAAHRVLGYSSDFWYGVEGSFEEPMGRESLQRLWQKYKVDIAFYGHVHNYERTCPIYQNQC
VNDERSHYSGVVNGTIHVVAGGAGSHLSNFSQVTPKWSLYRDYDFGFVKLTAFSHSSLLFEYKK
SSDGKVYDSFTISRDYKDVLACVHDSCEATTSAT*

>GmPAP7
MDRLASWSLEIGEEKELTTNPKLHSSGQTSGHRYSRETPNSYSRGGAANEYRAFPCYTKWSLYG
SYWRAIRHRFCIDDPAFDDSFTKIYTASSLQKQWYSVLGNHDYRGNVEARLSPVLTNLDKRWLC
LRSFTVNAEVAEFYFVDTTPFVDKYFTEPKDMSIYDWSGILPRKQYISNLLKDVDLALQQSNAK
WKIVVGHHTIRSAGLHGNTDELVKQLLPILEANNIDLYINGQDHCLQHIGSLGSAIQFLASGGGSK
AWRGVVNWWKPEEMKFYYDGQGFMSVKITETEIDIVFYDVYGHVLHKWNAS*
>GmPAP8
MASSLNQRMLFPLMFVIGMFCLMVTPSIAELPIFKHPPKKQQSLNILVLGDWGRKGTYNQSLVA
NQMGIVGEKLDIDFVISTGDNFYEDGLKGVDDPAFYQSFVDMYTAPSLQKTWYTVLGNHDYRG
DVGAQLSPILKQKDSRWLCMRSFILDGEIVEFFFVDTTPFVEEYFTDPGEHTYDWEGVLPRLAYL
SELLKDVDLALAQSKAKWKMVVGHHTINSAGHHGNTEELKQLLVPILEANDVDAYINGHDHCL
QHIIDNNSGIHFITSGGGSKAWSGDVKPWKLEELKLYYDGQGFMSMQITKSKADIIFYDAFGKVL
HTWSISKDRNVAAWI*
>GmPAP9
MTDISKSLLFTFTIIISFGLCILYASAELQRFAHSSKHDGALSFLVLGDWGRRGAYNQSLVAFQMG
KVGEKLDIDFVVSTGDNFYDNGLTSDHDNAFQESFTKIYTAKSLQNQWYSVLGNHDYRGDAEA
QLSPVLREIDSRWLCLRSFIVDSELVEIFFVDTTPFVDEYFTEPPEHKYDWRGIGPQKSYISNLLKD
LELALRGSTAKWRIVVGHHAIRSVGHHGDTQELINRLLPILQANNVHFYMNGHDHCLEHISDTES
PIQFLTSGAGSKAWRGDIEGMNRRGVNFFYDGQGFMSVKLTQTDATIEFYDVFGNVLHRLTSSK
QLHSSM*
>GmPAP10
MAGLGIWMGFVSLCLLSVSVSGLLQRLEHPVKADGSLSLMVIGDWGRKGTYNQSQVATQMGR
VAAKLNIDFVISTGDNFYDDGLTGIDDPAFEISFSKIYTAKSLQKQWYSVLGNHDYRGDVEAQLN
PILQKIDPRWICQRSFIVDTEIAEFFFIDSTPFVDKYFLKPKDHKYDWRGVLPREKYLSKLLKDLEI
ALKDSTAKWKIVVGHHPVRSIGHHGDTKELIRQLLPILEENNVDMYINGHDHCLEHISSRSSQIQF
LTSGGGSKAWKGDMDKDKKDGIKFYYDGQGFMSVELEETNAKVVYFDIYGKVLHVVNLPKGL
GTSVSAI*
>GmPAP11
MDEKTTTFVRDDSLSVDMPIDSDVFRVPPGYNAPQQVHITQGDHVGKGVIISWITPHEPGSSTVK
YWAENSEFELKAHGFYLAYKYFNYTSGYIHHCTIHNLEFDTKYYYEVGIGNTTRQFWFKTPPPV
GPNVPYTFGLIDDFPYHDNTKWDTWGRFTERIAAYQPWIWTAGNHEIDFAPELGETRPFKPYTC
RYHLPYTASNSTSPLWYSIKRASTYIIVLSSYSAFGKYTPQYKWLVKELPKVNRTETPWLIVLMH
SPMYNSYVNHYMEGETVRVLYEKWFVEYKVDVVFAGHVHAYERSKRVSNIAYSIVNGLHNPIN
DQSAPVYITIGDGGNIEGLATAMTEPQPSYSAYREASFGHGILDIKNRTHAYFSWNRNQDGLPSW
VTPACHPSGGIRVMEQSSGLLGWALAAFCWPVRSKLAANTSGFQQWRDNRLLQQQSTMA*EE
MGLNWHRWVVSAFLLVAWALILDTGEICFCMGGKTSDFLRNDYLSLDMPIDSDTFRVPPGYNA
PQQVHITQGDHVGKGVIISWISPHEPGSSTVIYWAENSEFKWQAHGFFLTYKYFNYTSGYIHHCT
VHNLEFDTKYYYEVGIGNTTRQFWFKTPPPVGPDVPYTFGLIGDLGQTYNSNRTLTHYEQSPAK
GQTILYVGDLSYADDYPLHDNIRWDTWGRFTERIAAYQPWIWTAGNHEIDFAPQLGETRPFKPY
TARYHVPYKASDSTSPLWYSIKRASAYIIVMSSYSALGKYTPQYKWLEKELPKVNRTETPWLIVL
MHSPIYNSYVTHYMEGETVRVMYEKWFVEYKVDVVFAGHVHAYERSERVSNIAYNVVNGLCR
PINDQSAPVYITIGDGGNLEGLATAMTEPQPSYSAYREASFGHGILDIKNRTHAHFSWNRNQDGY
AVVADSVWLHNRYWNHPEQTSLAAFRILNMMVMIFPIPSIAYEPRGEVGDNEACDPSGGFRVIE
QLSGLLGWTVAAICGPARSKLGVEPRVLSEGPSFVAPRLEGTA*
>GmPAP12
MEGFFGNCFNMILLMLCFTNLSIAFAQSHMNGFGEQPLAKIAIHKTVLALHSSASIIAVPFVLGTK
GEDTQWVTVELESPIPSVDDWVGVFSPANFNSATCPDTDGIGWVEEPYICTAPIKYKYANYSNRN

YAKTGKAILKFQLINQRADFSFALFSGGLSDPRLVAISNSISFANPKAPVYPRLALGKSWDEMTVT
WTSGYDINEAVPFVEWGPKGGKKTRSHAGTLTFNRNSMCGEPARTVGWRDPGFIHTSFLKELW
PNFRYTYKLGHMLSNGSYVWSKKYSFKASPYPGQNSLQRVIIFGDMGKAERDGSNEYADYQPG
SLNTTDQLVKDLENYDIVFHIGDMPYANGYISQWDQFTAQVQEISSTVPYMIASGNHERDWPNT
GSFYDTPDSGGECGVPAETMYYFPAENRAKFWYKADYGLFRFCIADSEHDWREGSEQYKFIEHC
LATVDRKHQPWLIFSAHRPLGYSSNLWYGMEGSFEEPMGRESLQKLWQKYKVDIGFYGHVHNY
ERVCPIYQNQCVNEEKHHYSGTVNGTIHVVVGGGGSHLSDFTPSPPIWSLYRDVDYGFGKLTAF
NHSYLLFEYKKSSDGEVYDSFTISRDYRDVLACVHDGCEKTTLAT*

>GmPAP13
MSMSFLFTFTIIISFGLCILYASAELQRLSHSSKHDGALSFLVLGDWGRRGAYNQSQVSFQMGKV
GEKLDIDFVVSTGDNFYDNGLTSDHDNAFQESFTQIYTAKSLQKQWYSVLGNHDYRGDAEAQL
SPVLREIDSRWLCLRSFIVDSELVEIFFVDTTPFVEEYFTEPQEHKYDWRGIGPQKPYITNLLKDLE
LALRESTAKWKIVVGHHAIRSVGHHGDTQELINQLLPILQANNIDFYMNGHDHCLEHISDTESPIQ
FLTSGAGSKAWRGDIKGMNRRDVNFFYDGQGFMSVKLTQTDATIEFYDVFGNVLHRLTSSKQL
HSSM*

>GmPAP14
MGLQLVFIGTIALCLVVSSAVLERFEQALKQDGSLSFLVIGDWGRKGAYNQSKVAFQMGVIGQQ
LDIDFVISTGDNFYDSGLTGIDDPDFDTASSLQKQWYSVLGNHDYRGNVEAQLSPVLTNLDKRW
LCLRSFICLFTFFVSEVAEFFFVDTTPFVDKYFIEPKDHVYDWSGILPRKQYISNLLKDVDLALQQS
NAKWKIVVGHHTIRSAGVHGNTDELVKQLLPILEANNIDLYINGHDHCLQHISSFDSAIQFLTCGG
GSKAWRGVVNWWKPEEMKFYYDGQGVMSVKVTETEIDIVFYDVYGHVLHKWNTSKQLHASW
*

>GmPAP15
MGTQRSKPSCTIVAIFLAFCCFVSSSKAKLESLQHAPKADGSLSFLVVGDWGRKGAYNQSLVAF
QMGVIGEKLDVDFVISTGDNFYDNGLTGVFDPSFEESFTKIYTAPSLQKKWYNVLGNHDYRGNA
KAQISHVLRYRDNRWVCFRSYTLNSENVDFFFVDTTPFVDKYFIEDKGHNYDWRGILPRKRYISN
LLKDVDLALRQSTATWKVVIGHHTIKSIGHHGDTQELLIHFLPLLKANNVDLYINGHDHCLEHIS
SLDSSVQFLTSGGGSKAWRGDTKQSEGDEMKFYYDGQGFMSVHISQTQLRISFFDVFGNAIHKW
NTCKFDSCDM*

>GmPAP16
MASSLNQRMVFPVMVVVGMFCLLVTPSIAELPRFKHPPKKQQSLNILVLGDWGRKGTYNQSLV
ANQMGIVGEKLDIDFVISTGDNFYEDGLKGVDDPAFYQSFIHMYTAPSLQKTWYTVLGNHDYR
GDVEAQLSPILKQKDSRWLCMRSFILDGEIVEFFFVDTTPFVEEYFTDPGEHTYDWEGVLPRLAY
VSKLLKDVDSALAQSKAKWKMVVGHHTINSAGHHGSTEDLKQLLVPILEANNVDAYINGHDHC
LQHIIDNNNGIHFITSGGGSKAWSGDVKPWKLEELKLYYDGQGFMSMQITKSTAYIIFYDAFGKV
LHTWSISKDRNVAAWI*

>GmPAP17
MMMSGMGNSRVLIFSLLVLATFQQVVSDEHQPLSKVAIHKTTLALDERAYIKATPSVLGLKGQN
TEWVTLQYSNPKPTIDDWIGVFSPANFNASTCPAENIWVNPPFLCSAPIKYQYANFSSHGYKNTG
KGSLKLQLINQRSDFSFALFTGGLTNPKLVAVSNKVSFINPNAPVYPRLAQGKTWDEITVTWTSG
YGISDAEPFVEWGPKGGNLVKSPAGTLTFDHNTMCGAPARTVGWRDPGYIHTSFLKELWPNQE
YKYKLGHRLFNGTIIWSQEYQFKASPFPGQNSLQRVVIFGDLGKAEADGSNEYNNFQPGSLNTTK
QIVQDLKDIDIVFHIGDLCYASGYLSQWDQFTAQIEPIASTVPYMTASGNHERDWPDTGSFYGTL
DSGGECGVPAQTTFYVPAENREKFWYSVDYGMFRFCIANTELDWRKGSEQYKFIENCLATVDR
QKQPWLIFLAHRVLGYSSAGFYAAEGSFEEPMGREDLQYLWQKYKVDIAMYGHVHNYERTCP

VYQNICTNKEKNNYKGSLDGTIHVVVGGGGASLAEFAPINTTWSIFKDHDFGFVKLTAFDHSNFL
FEYKKSSDGQVYDSFRISREYRDILACTVDSCPATTLAS*
>GmPAP18
MLMIIDSPELEVHCCRAADPTFAEVSVVFTVHTQMELKLLLITVLMMVSLSATAAADYIRPQPRK
TFHLPWHSKPSSYPQQVHISLAGEQHMRVTWITDDNSAPSIVEYGTSPGRYDSVAEGETTSYSYL
LYSSGKIHHTVIGPLEHNSVYYYRCGGQ
GPQFQLRTPPAQLPITFAVAGDLGQTGWTKSTLDHIDQCKYNVHLLPGDLSYADYIQHRWDSFG
RLVQPLASARPWMVTQGNHEVESIPLLKDGFLSYNSRWKMPFEESGSNSNLYYSFEVAGVHIIM
LGSYADYDEYSEQYGWLKEDLSKVDRERTPWLIVLFHVPWYNSNTAHQGEGADMMASMEPLL
YAASADLVLAGHVHAYERSKRVYNKRLDPCGSVHITIGDGGNKEGLAPKYINPQPIWSEFREASF
GHGELQIVNSTHAFWSWHRNDDDEPVKSDDIWITSLTSSGCVDQKRNELRNKLMTP*
>GmPAP19
MASITFSLLQFHRAPILLLILLAGFGHCHIPSTLEGPFDPVTVPFDPALRGVAVDLPETDPRVRRRV
RGFEPEQISVSLSTSHDSVWISWVTGEFQIGLDIKPLDPKTVSSVVQYGTSRFELVHEARGQSLIYN
QLYPFEGLQNYTSGIIHHVQLKGLEPSTLYYYQCGDPSLQAMSDIYYFRTMPISGSKSYPGKVAV
VGDLGLTYNTTTTIGHLTSNEPDLLLLIGDVTYANLYLTNGTGSDCYSCSFPLTPIHETYQPRWDY
WGRFMQNLVSNVPIMVVEGNHEIEKQAENRTFVAYSSRFAFPSQESGSSSTFYYSFNAGGIHFIM
LGAYINYDKTAEQYKWLERDLENVDRSITPWLVVTWHPPWYSSYEAHYREAECMRVEMEDLL
YAYGVDIIFNGHVHAYERSNRVYNYNLDPCGPVYITVGDGGNREKMAIKFADEPGHCPDPLSTP
DPYMGGFCATNFTFGTKVSKFCWDRQPDYSAFRESSFGYGILEVPFLSLPHVPTPSMVVQMLFIF
NKLIYL*

>GmPAP20
MGVVEGLLALALVLSVCVMCNGGSSSPFIRKVEKTVDMPLDSDVFAVPPGYNAPQQVHITQGD
LVGKAVIVSWVTVDEPGSSEVHYWSENSDKKKIAEGKLVTYRFFNYSSGFIHHTTIRNLEYKTKY
YYEVGLGNTTRQFWFVTPPEIGPDVPYTFGLIGDLGQSFDSNKTLSHYELNPRKGQTVLFVGDLS
YADNYPNHDNVRWDSWGRFTERSVAYQPWIWTAGNHEIDFAPEIGETVPFKPYTHRYHVPYKA
SQSTSPFWYSIKRASAHIIVLASYSAYGKYTPQYKWLEEELPKVNRTETPWLIVLMHSPWYNSYN
YHYMEGETMRVMYEPWFVQYKVDVVFAGHVHAYERSERVSNVAYNIVNGLCAPVKDQSAPV
YITIGDGGNLEGLATNMTEPQPEYSSFREASFGHAIFDITNRTHAHYSWHRNQDGVAFEADSVWF
FNRYWHPVDDSTAHVSH*
>GmPAP21
MRVSWITDDKHSESVVEYGTKKGEYSTKATGEHTSYHYFLYESGKIHHVVIGPLQPNTIYYYRC
GGSGSEFSFKTPPLKLPIEFVVVGDLGQTEWTTSTLKHVDSKDYDVFLLPGDLSYADTHQPLWDS
FGRLVEPYASRIPWMVTEGNHEIETFPIIQPNGFKAYNARWPMPYKESGSTSNLYYSFDVASTHVI
MLGSYTDFDAHSQQYTWLQSDLAKIDRKRTPWVIALLHAPWYNTNEAHQGEGEDMRQAMEEL
LYEARVDLVFAGHVHAYERFTRIYDNKADSCGPLYVTIGDGGNREGLALSSSLPWMVRDGNQEI
ETFPPLQPKRFQGLQCSPAHALRGSGCTSYLYHSFDVARTHVKMWDSHTHLHTHSHPYTWLQS
DLDKSDRKRTPRVIAYLHAPRYNNNEAHQGEGEDMRQAREELLYVVRVDLFKKPPSPLSLYREP
SFGHGRLRIVNETHAYWSWHRNNDTDTFVADGVWIESLSNSKACWNAQGQHVAHEEL*
>GmPAP22
AIVDGGGVPTTLDGPFKPVTVPLDQSFRAVDLPDTDPLVQRTVQGFQPEQISLSLSVSHDSVWIS
WITGEFQIGDNIEPLDPETVASIVQYGRLGRSMRHNATGYSIVYSQLYPFEGLQNYTSGIIHHVRL
TGLRPNTLYQYQCGDPSLSAMSDVHYFRTMPVSGPKSYPSRIAVVGDLGLTYNTTSTVDHMTSN
HPDLILLVGDVSYANLYLTNGTGADCSSCSFSNTPIHETYQPRWDYWGRYMQPLISSVPVMVIEG
NHEIEEQAENQTFVAYSSQFAFPSEESGSSSTFYYSFNAGGIHFIMLGAYISYDKSGDQYRWLERD

LASVDREVTPWLIATWHAPWYSTYGAHYREAECMRVEMEDLLYKYGIDIVFNGHVHAYERSN
RVYNYTLNPCGPVYITVGDGGNREKMAITHADEPGQCPEPSTTPDDYMX*
>GmPAP23
MGVVEGLLALGLILNVCVVCNGGTSSPFVRKVEKAVDMPLDSDVFAIPPGYNAPQQVHITQGDL
VGKAVIVSWVTVDEPGSSEVRYWSENSDQKKIVEGKLVTYRFFNYTSGFIHHTTIRNLEYNTKY
YYEVGLGNTTRQFWFVTPPEIGPDVPYTFGLIGDLGQSFDSNKTLSHYELNPRKGQTVLFVGDLS
YADNYPNHDNIRWDSWGRFTERSVAYQPWIWTAGNHEIDFAPEIGETVPFKPYTHRYHVPYKAS
QSTSPFWYSIKRASAHIIVLASYSAYGKYTPQYKWLEKELPKVNRTETPWLIVLMHSPWYNSYN
YHYMEGETMRVMYEPWFVQYKVDVVFAGHVHAYERSERVSNVAYNIVNGLCAPVNDKSAPV
YITIGDGGNLEGLATNMTEPQPKYSAFREASFGHAIFDITNRTHAHYSWHRNQDGVAVEADSLW
FFNRYWHPVDDSTAHVSH*
>GmPAP24
MPLHSDVFVAPSGYNAPQQVHITQGDQVGRAMIVSWVTVDEPGKSLVHYWSDASQHKRVAKG
NHVTYRYFNYSSGFIHHCTLRDLEFNTKYYYEVGIGHTTRQFWFVTPPEVHPDAPYTFGLIGDLG
QTFDSNKTLVHYESNPHKGQAVLYVGDLSYADNHPNHDNVRWDTWGRFVERSTAYQPWIWTT
GNHELDYAPEIDETEPFKPFRHRYYVPYKASGSTEPFWYSVKIASAHIIVLASYSAYGKYTPQYE
WLEAELPKVDRTKTPWLIVLVHSPWYNSYNYHYMEGETMRVMFEPWFVKYKVDVVFAGHVH
AYERSERISNTGYNITNGRCRPLKDQSAPVYINIGDGGNIEGLASNMTNPQPEYSAYREASFGHAI
LEIKNRTHAHYSWHRNEDEYAVTADSMWFFNRYWHPVDDSTTK*
>GmPAP25
MFQYSKAMLLNLVLVSFVLLSSIRDGSAGITSSFVRPQWPGVDIPVDHEVFAVPKGYNAPQQVHI
TQGDYDGKAVIVSWVTPDEPGTRHVQYGTSKDKFKTSAEGTVANYTFYNYKSGYIHHCLIEGLE
YKTKYYYRIGSGDSARDFWFETPPKVGPDTPYKFGIIGDLGQTFNSLSTLEHYLESGGEAVLYVG
DLSYSDEHDYKDMGLRWDTWGRFAERSAAYQPWMWNVGNHEVEFLPEVGEVEPFKNYLYRY
TTPYSASKSTSPLWYAVRRASAHIIVLSSYSPFVKYTPQYIWLKEELARVDRKKTPWLIVLVHKPL
YSSNVAHYMEGEAMRSVFETWFVQYKVDVIFAGHVHAYERSYRYSNIDYNITGGRRYPIPDKSA
PIYITIGDGGNLEGLASSYLDPQPEYSAFREASYGHATLEIKNRTHAIYHWYRNDDGKKVPADSL
VLHNQYWGSNGRKQNEVIDEVVNVK*
>GmPAP26
MEGAKSCSCFATISVALVLLLLPNVAVVCHGGKTSTFIRKVEKTEDMPLHSDVFVSPSGYNAPQQ
VHITLGDQVGRAMIVSWVTLDEPGKSLVHYWSDDCPHKRVAKGNHFTYRYFNYSSGFIHHCTL
RDLEFNTKYYYEVGIGHTTRQFWFVTPPEVHPDTPYTFGDLGQTFDSNKTLAHYESNPHKGQAV
LYVGDLSYADNYPNHDNVRWDTWGRFVERSTAYQPWIWTTGNHELDYAPEIDETEPFKPFRHR
YHVPYQASGSTEPFWYSIKIASAHIIVLASYSAYGKYTPQYEWLEAELPKVDRTKTPWLIVLVHS
PWYNSYSYHYMEGETMRVMFEPWFVKYKVDVVFAGHVHAYERSERISNIGYNITNGRCGPSKD
QSAPVYINIGDGGNIEGLARNMTNPQPEYSAYREASFGHAILEIKNRTHAHYSWHRNEDEYAVT
ADSMWFFNRHWHPVDDSTTTK*
>GmPAP27
MIERKIEAEVHCCLSQVVTVAVIMELKQQLLLLILTLLFATATPQYVRPLPRKTLTIPWDSISKAHS
SYPQQVHISLAGDKHMRVTWITDDKHSPSYVEYGTLPGRYDSIAEGECTSYNYLLYSSGKIHHA
VIGPLEDNTVYFYRCGGKGPEFELKTPPAQFPITFAVAGDLGQTGWTKSTLAHIDQCKYDVYLLP
GDLSYADCMQHLWDNFGKLVEPLASTRPWMVTEGNHEEENILLLTDEFVSYNSRWKMPYEESG
STSNLYYSFEVAGVHVIMLGSYADYDVYSEQYRWLKEDLSKVDRKRTPWLLVLFHVPWYNSN
KAHQGAGDDMMAAMEPLLYAASVDLVIAGHVHAYERSKRVYNGRLDPCGAVHITIGDGGNRE
GLAHKYINPQPKWSEFREASFGHGELKIVNSTHTFWSWHRNDDDEPVKADDIWITSLASSGCVD
QKTHELRSTLLTP*
>GmPAP28

MWLASFRSLLCKCFIPRWLGLCRLIKTTLIPLERRMLLAMLLNLVLASFVFLSFIRDGSAGITSSFI
RSEWPAVDIPLDHEAFAVPKGYNAPQQVHITQGDYDGKAVIISWVTTEEPGHSHIQYGTSENKFQ
TSEEGTVTNYTFHKYKSGYIHHCLIEGLEYETKYYYRIGSGDSSREFWFKTPPKVDPDSPYKFGII
GDLGQTFNSLSTLEHYIQSGAQTVLFVGDLSYADRYQYNDVGLRWDTWGRFVERSTAYHPWL
WSAGNHEIDYMPYMGEVVPFKNYLYRYTTPYLASNSSSPLWYAVRRASAHIIVLSSYSPFVKYT
PQYMWLKEELKRVEREKTPWLIVLMHVPLYNSNGAHYMEGESMRSVFESWFIEYKVDVIFAGH
VHAYERSYRYSNVDYNITGGNRYPLPNKSAPVYITVGDGGNQEGLASRFLDPQPEYSAFREASY
GHSTLEIKNRTHAIYHWNRNDDGKKVPTDSFVLHNQYWGHNRRRRKLKHFLLKVIDEVASM*
>GmPAP29
MLFSLSCVIVDGGVPTTLDGPFKPVTVPLDQSFRGNAVDLTDTDPLVQRTVEGFQPEQISLSLSAS
HDSVWISWITGEFQIGDNIEPLDPETVASIVQYGRFGRSMRHQATGYSLVYSQLYPFEGLQNYTS
GIIHHVRLTGLRPNTLYQYKCGDPSLSGMSDVHYFRTMPASGPKSYPSRIAVVGDLGLTYNTTST
VNHMTSNHPDLILLVGDVSCANLYLTNGTGADCYSCSFPNTPIHETYQPRWDYWGRYMQPLISS
VPIMVIEGNHEIEEQAENQTFVAYSSRFAFPSEESGSSSTFYYSFNAGGIHFIMLGAYISYDKSGDQ
YKWLERDLASVDREVTPWLIATWHAPWYSTYKAHYREAECMRVEMEDLLYKYGVDIVFNGH
VHAYERSNRVYNYTLDPCGPVYITVGDGGNREKMAITHADEPGQCPEPSTTPDDYMGGFCAFNF
TSGPAEGNFCWDRQPDYSAFRESSFGHGILEVKNETHALWIWHRNQDFYGSAGDEIYIVREPQN
CPPIKPELVIQFRNYHEQNLFFNLKVFFNFVGGC*
>GmPAP30
MLSFYNFECISDDDEWDGEKAVSEEHQPLSKVAIHKTTLALDERAFIKATPNVLGLKGQNTEWV
TLQYSNPKPTVDDWIGVFSPANFNASTCPAENIWVNPPFLCSAPIKYQYANFSSHGYKNTGKGSL
KLQLINQRSDFSFALFTGGLTNPKLVAVSNKVSFINPNAPVYPRLAQGKTWDEMTVTWTSGYEIS
DAEPFVEWGPKGGNLVKSPAGTLTFDRNTMCGAPARTVGWRDPGYIHTSFLKELWPNREYKYK
LGHKLFNGTIIWSQEYQFKASPYPGQNSLQRVVIFGDMGKAEADGSNEYNNFQPGSLNTTKQIIQ
DLKDIDIVFNIGDLSYANGYLSQWDQFTAQIEPIASTVPYMTASGNHERDWPDTGSFYGNLDSGG
ECGVLAQTMFYVPAENREKFWYSVDYGMFRFCIANTELDWRKGSEQYKFIENCLASVDRQKQP
WLIFLAHRVLGYSSAGFYVAEGSFEEPMGREDLQYLWQKYKVDIAMYGHVHNYERTCPVYQNI
CTNKEEHNYKGSLDGTIHVVVGGGGASLAEFAPINTTWSIFKDHDFGFVKLTAFDHSNLLFEYK
KSSDGQVYDSFKISRQYRDILACTVDSCPPTTLAS*
>GmPAP31
MAVAETHIPTTLDGPFDPVTRRFDPSLRRGSDDLPMTHPRLRKNVTSNFPEQIALAISSPTSMWVS
WVTGDAQIGLNVTPVDPASVGSEVWYGKKSGKYTSVGKGDSVVYSQLYPFEGLWNYTSGIIHH
VKLKGLEPGTRYYYKCGDSSIPAMSQEHYFETFPKPSPNNYPARIAVIGDLGLTSNSTSTIDHLNY
NDPSMILMVGDLTYANQYLTTGGKGASCYSCAFPDAPIRETYQPRWDGWGRFMEPLTSEIPMM
VIEGNHEIEPQAGGITFKSYLTRFAVPAEESGSKSNFYYSFDAGGIHFIMLGAYVDYNSTGAQFA
WLKKDLQSVDRSVTPWLVAAWHSPWYNSYASHYQEFECMRLEMEELLFRYRVDIVFDGHVHA
YERMNRVFNYTLDPCGPVYITVGDGGNIEKVDVDHADDPGKCPSAGDNIPEFGGVCKSNFSTGP
AKGNFCWNKQPEWSAFRESSFGHGILEVVNSTYALWTWHRNQDNYKENAVGDQIYIVRQPEX*
>GmPAP32
MRVSWITEDKHAESVVEYGTKAGEYSAKATGVYTSYQYFFYNSGKIHNVVIGPLQPGSTYFYRC
GGSGPEFSFKTPPPRCPIEFVIVGDLGQTEWTASTLKHIDSSDYDVFLLPGDLSYADSQQPLWDSF
GRLVEPYASKRPWMVTEGNHEIEIFPIIYPQGFQAYNARWPMPFQQSGSTSNLYYSFEVAGTHVI
MLGSYTDFDSQSLQYTWLQSDLANIDRVKTPWVIVLLHAPWYNTNEAHQGEGESMRQAMEEL
LYEARVDLVFAGHVHAYERFTRIYDNKADSCGPMYVTIGDGGNREGLALMFKNPSSPLSLYREP
SFGHGRLRILNETHAHWSWHRNNDADAVVADGVWIESLSSSKACSKTPYQQYAANEEL*
>GmPAP33

MGMRMRVPGFMLLLIIGIFELDAVYGYVRPPPRKTLFVPHADQDSHSPQQVHISQVGQNKMRIS
WITDSPTPAKVMYAPSPSGNTVSATGTTSSYRYLVYESGEIHNVVIGPLNPNTVYYYRLGDPPSS
QTYNFKTPPSQLPIKFAIVGDLGQTDWTKSTLEHVKKSNYDMLLLPGDLSYADFNQDLWDSFGR
LVEPLASQRPWMVTQGNHEVETIPLLHKTPFTAYNARWLMPFQESGSNSNLYYSFDVAGVHVI
MLGSYTDFDPSSPQYKWLQNDLQTVNKRTTPWIVVLIHAPWYNSNTAHQGEPESINMKVAMED
LLYQARVDVVFAGHVHAYERFTRVYKDKANNCAPMYITIGDGGNREGLATKYMDPKPTISIFRE
ASFGHGTLEVFNVSHARWTWHKNDNDEAVDSDFVWLTSFSSIPSCK*
>GmPAP34
MASPSSPLHSAAILCIYFVLPAFAELQRFQHQPKHDGSLNFLVIGDWGRKGHYNQSLVATQMGK
MGDKLDLDFVVSTGDNFYNSGLKGVNDPLFLQSFSNIYTAKSLRKQWYSVLGNHDYRGNALAQ
LSPLLRKIDRRWFCQRSFILNAGVAEFFFIDTTPFMRKYFNNSNRHYDWRGVLPRQKYLKTLLKD
LEEELRKSTARWKIAVGHHAIRSIGHHGDSPELVKHLVPVLKANNVDMYINGHDHCLQHISSTDS
PLLYLTSGAGSKAWRGDVKETHFDVKFFYDGQGFMSVQMTENDTNFAFYNVFGEKIHHWKVT
KSTMHPSI*
>GmPAP35
MIPDLPLPFLFSLFIIFFHLAESKPSLTATPTTLPASGATVNLRWSGIPSPSDLDFLAIYSPPTSPHDN
FIGYLFLSQSATWRTGSGNLSLPLVDLRSNYSFRIFSWTRAEINPKRQDHDHNPLPVTRHLLAFSE
EVSFAPHRGPQQIHLAFVGAHGKEEDMRVMYITRDPRETYVRYGEREDKLDGIAVARVERYERE
HMCDAPANTSVGWRDPGFIHDAVLIGLKKGQRYYYKVGNDNGGWSATQSFVSRNSDSDETIAF
LFGDMGTAVPYNTFLRTQDESISTMKWILRDVEALGDTPAFVSHIGDISYARGYSWLWDHFFAQI
EPVASQVAYHVCIGNHEYDWPLQPWKPDWASYGKDGGGECGVPYSLRFNMPGNSSELTGNAA
APPTRNLYYSFDMGAVHFVYISTETNFVPGSKQYDFLKHDLESVNRSKTPFVVVQGHRPMYTTS
HENRDAALRGKMLEHLEPLLVNNNVTLALWGHVHRYERFCPLNNFTCGVNAGHNAGDKKGYT
VHIVIGMAGQDWQPVWEPRPDHPDDPIFPQPKWSLYRGGEFGYTRLVATKQKLVLSYVGNHDG
EVHDQLEILASGEVVSGDGGCSIADANSKAGNVIVESTLSWYVKGGSVLLLGAFMGYVFGYVTS
ARKKSEVPESNWTPVKTEET*
>CaPAP18c
MELIKFILVTISTLLISATITAEYVRPLPRNTLNIQWPWDSKSHSYPQQVHISLAGDKHMRITWITE
DKHSSSFVEYGTLPGRYDSMAEGECTSYNYLLYSSGKIHHTVIGPLEHDTVYFYRCGGQGPEFQL
KTPPAQYPITFAVAGDLGQTGWTKSTLDHIDQCKYDVYLLPGDLSYADCMQHLWDSFGRLVEP
LASARPWMVTEGNHEEENILFLTDEFVSYNSRWKMPFEESGSTSNLYYSFEVAGVHVIMLGSYA
DYDESSEQYTWLMADLSKVDRTRTPWLLVLFHVPWYNSNRAQQGAGDDMMAVMEPLLYAAS
VDLVIAGNVHAYERSKRVYNGRLDRCGAVHITIGDGGNREGLAHRYLLNFRFLDRKVNVHISLA
GDKHMRITWITEDKHSSSFVEYGTLPGRYDSMAEGECTSYNYLLYSSGKIHHTVIGPLEHDTVYF
YRCGGQGPEFQLKTPPAQYPITFAVAGDLGQTGWTKSTLDHIDQCKYDVYLLPGDLSYADCMQ
HLWDSFGRLVEPLASARPWMVTEGNHEEENILFLTDEFVSYNSRWKMPFEESGSTSNLYYSFEV
AGVHVIMLGSYADYDESSEQYTWLMSDLSKVDRTRTPWLLVLFHVPWYNSNKAHQGAGDDM
MAVMEPLLYAASVDLVIAGHVHAYERSKRVYNGRLDRCGAVHITIGDGGNREGLAHRYINPQP
NWSEFREASFGHGELKIVNSTHAFWSWHRNDYDESVKADDIWITSLVGSGCVDHKRHELRSML
MTP*
>CaPAP26
MKPLLLHLVLATFVFFSSIRDGCAGITSSFVRSDWPSVDIPLNHEAFAVPKGYNAPQQVHITQGD
YDGKAVIISWVTPEEPGSNHVQFGTSENKFKATAEGTVSNYTFYKYKSGYIHHCLVEGLEYNTK
YYYRIGSGESSREFWFETPPKVGPDAPYKFGIIGDLGQTFNSLSTLEHYIESGAQAVLFVGDLSYA
DRYEYTDVGLRWDTWGRFVEKSTAYQPWMWSAGNHEIEYMPYMGEVVPFKSYLQRYSTPYL
ASKSSSPLWYAFRRASAHIIVLSSYSPFVKYTPQWQWLREELKNVDREKTPWLIVLMHVPLYNS
NDAHYMEGESMRVVFESWFIEYKVDMIFAGHVHAYERSYRFSNVDYNITSGNRYPVADKSAPV

YITVGDGGNQEGLASRFMDPQPEYSAFREASYGHSTLEIKNRTHAIYHWNRNDDGKKVPTDSFV
LYNQYW
>CaPAP10
MDMPLDSDVFDVPQGYNAPQQVHITQGDLVGKAVIVSWVTEDEPGSNAVRYWSENSHRKKLA
KGKIQTYRFFNYTSGFIHHATIRKLKYNTKYYYEVGLENTTRQFWFITPPEIGPDVPYTFGLIGDL
GQSFDSNKTLSHYELNPRKGQTVLFVGDLSYADNYPNHDNVRWDTWGRFTERSVAYQPWIWT
VGNHEIDFAPEIGEPKPFKPYSHRYHTPYKASQRKYTPQYKWLEQELPTVNRTETPWLIVLMHSP
WYNSYAYHYMEGETMRVMFESWFVKYKVDVVFAGHVHAYERSERVSNIAYNIVNGICTPIKD
QSAPVYITIGDGGNLEGLATNMTEPQPEYSAFREASFGHAIFDIKNRTHAHYSWHRNQDGYAVQ
ADSLWFFNRFWNPLDDSTTHVSH
>CaPAP22
MVKCNMSLVVIPFLLHILWFLMFPHTIQSQGNAFSRQPTSQFIFTPHSLSDSDPEQVHISLVGKDH
MRVSWITEEKDTESIVEYGTKEGEYSKKSIGDHTSYNYFLYKSGKIHHVVIGPLNPNTTYFYRCG
GSGPEFSLKTPPSKLPIDFVVVGDLGQTEWTKSTLKHIDSKDYDVFLLPGDLSYADSHQPFWDSY
GRLVEPYASRRPWMVTEGNHEIETIPIIQPHAFKSYNARWIMPYNESGSTSNLYYSFEVASSHIIM
LGSYTDFDAQSKQYKWLQYDLLNIDRNITPWVIVLLHAPWYNTNEAHQGEGESMRQAMEELLY
EARVDMVFAGHVHAYERFTQIYDNKHDSCGPLYITIGDGGNREGLALKFKQPQSPLSMYRESSF
GHGRLRIVNETHAHWSWHRNNDADAFVADNVWMNSLSNSKECWETLKQQVSHGEL
>CaPAP21
MAKYTKSLISFPILLHTLCFLLFPQPLLSQDNDFVRQPASQLIITPHQRSNSDPQQVHISLVGKDKM
RVSWITEDNEAKSVVEYGTKEGVYSEKSMGEHTSYQYFFYNSGKIHNTVIGPLEPNTTYFYKCG
GLGPEFSFKTPPSKFPIEFVIVGDLGQTEWTASTLKHVDKSDYDVFLLPGDLSYADSQQPLWDSF
GRLVEPYASKRPWMVTEGNHEIEIFPIIYPNGFKAYNARWPMPFQQSGSNSNLYYSFEVAGTHII
MLGSYSDFNVESQQYNWLQLDLAKIDRVKTPWVITLLHAPWYNSNEAHQGEGESMRQAMEEL
LYEARVDLVFAGHVHAYERFTRIYDNQADSCGPMYVTIGDGGNREGLALKFEKPTSPLSLFREPS
FGHGRLRILNETHAHWSWHRNNDTNAIVADGIWIESLSNLKACSETPNNQVAHEEL*
>CaPAP15A
MPSRSFFRLFGFNYPMMTSLLPHFILLLFVPFTNCHIPTTLEGPFVPVTVPFDTSLRGVAVDLPDTD
PRVRRQVRGFEPEQISLSLSTTFDSVWITWITGEYQIGYNIKPLDPKIVSSVVQFGTSRFELVNEAK
GQSLIYNQLYPFEGLQNYTSGIIHHVRLTGLEPSTLYYYQCGDPSLHAMSDIYYFRTMPISDPQSY
PGRIAIVGDLGLTYNTTTTISHMTSNEPDLVLLIGDVTYANLYLTNGTGSNCYSCSFPQTPIHETY
QPRWDYWGRFMQNLVSRVPIMVVEGNHEIEKQARDKTFVAYSSRFAFPSEESGSSSTFYYSFNA
GGVHFIMLGAYINYGKTAEQYKWLERDLANVDKTITPWLIATWHPPWYSTYEAHYREAECMR
VEVEELLYSYGVDIVFNGHVHAYERSNRVYNYNLDPCGPVYITVGDGGNREKMAINFTDEPGH
CPDPSTTPDPYMGGFCATNFTFGPAASKFCWDRQPDYSAFRESSFGYGILEVKNETWALWTWYR
NQDSYKEVGDQIYIVRQPEICPVPQRLYRDCVASF*
>CaPAP15b
MVVDGGVGIPRTIDGPFKPVTVPLDKSFRGHAVDIPDTDPLVQRTVQGFQPEQISLSLSTSHDSV
WVSWITGEFQIGENIEPLDPEKVASIVKYGRFGRSINRQAVGYSLVYSQLYPFEGLQNYTSGIIHH
VRLTGLRANTLYQYQCGDPSLSAMSDIHYFRTMPVSGPKSYPSRIAVVGDLGLTYNTTSTVDHM
AINHPDLILLVGDASYANMYLTNGTSSDCYSCSFSNTPIHETYQPRWDYWGRYMETLISSVPIMV
VEGNHEIEAQAENKTFVAYSSRFAFPSEESGSSSTFYYSFNAGGIHFIMLGAYISYNKSGDQYKWL
ERDLASVDREVTPWLVATWHPPWYSTYKSHYREAECMRVEMEDLLYTYGVDIVFNGHVHAYE
RSNRVYNYTLDPCGPVYITVGDGGNREKMAITHADEPGNCPEPSTTPDDFMGGFCAFNFTSGPA
AGKFCWDQQPDYSAFRESSFGHGILEVKNETHALWSWHRNQDMYDIAGDAIYIVRQPDKCPPV
KTEG*

>CaPAP18b
MKELKLILTVLLLLSVTTIADDYVRPQPRKTLHLPWHSKSSSYPQQVHISFAGDKHMRVTWITDD
TSAPSVVEYGTLPGKYGSVAEGETTSYSYLFYSSGKIHHTVIGPLEPNSVYFYRCGGEGPEFELKT
PPSQFPITFAVAGDLGQTGWTKSTLDHIDRCKYDVNLIPGDLSYADYIQHRWDSFGRLVQPLASA
RPWMVTQGNHEVEHIPLIKDGFISYNSRWKMPFEESGSSSNLYYSFEVAGAHIIMLGSYADYDEY
SEQYTWLKTDLSKVDRKRTPWLLVLFHVPWYNSNTAHQGEGGDMMQTMEPLLYAANVDLVF
AGHVHAYERSKRVYNGRLDPCGAVHITIGDGGNKEGLAHKYITPQPKWSDFREASFGHGELKIV
NSTHAFWSWHRNDDDEPVKSDDIWINSLVSSGCVDQKRTEIP*
>CaPAP20
MRISWITHEPTSATVEYGPSPSANAFSANGETTFYNYVTYLSGHIHNVVIGPLKPNTVYYYRLGES
DKTYNLKTTPSQFPIKFAVVGDLGQTEWTVSTLQHIKDSNYDMLLLPGDLSYADVIQPLWDSFG
RLVEPLASERPWMVTTGNHDVEKIPIIHRTPFTAYNARWKMPFQESGSDSNLYYSFDVSGVHVI
MLGSYTDFDSDSAQYQWLKGDLENINRGKTPWVFVLVHAPWYNSNEAHQGERESVGMKASM
EDLLYQARVDVIFAGHVHAYERFKRVYNDESDDCAPIYINIGDGGNREGLAKKYQDPQPDISMF
REASFGHGTIDVVNASHALWSWHKNDNEERVASDTVWLTSLSSNHACIA*
>CaPAP27b
MKQKCNMIITLAVAALSCFLNSNIVLVFAHVNGFGEQPLSKIAIHKTVFSLHSNASVTAIPSLLGT
KGEDTQWVTLDIDFPDPSVDDWVGVFSPANFNSSTCPPLNDPKEQIPFICSAPIKYKFVNYSNSHY
TKTGKASLSFQLINQRADFSFALFSGGLSNPKLVAVSNFISFANPKVPLYPRLAQGKSWDEMTVT
WTSGYDINEATPFVEWGADGKMPVQSPAGTLTFGRNSMCGSPARTVGWRDPGFIHTSFLKNLW
PNLVYTYRLGHILSNGSYIWSKKYSFKSSPYPGQDSLQRVVIFGDMGKAERDGSNEYSNYQPGSL
NTTDQLIKDLENIDIVFHIGDISYANGYISQWDQFTAQVEPVASTVPYMIASGNHERDWPNTGSF
YDTTDSGGECGVLAETMFYVPAENRAKFWYATDYGMFRFCIADTEHDWREGSEQYKFIEHCLA
TVDRQKQPWLIFAAHRVLGYSSDFWYGLEGSFEEPMGRESLQRLWQKYKVDIAFYGHVHNYER
TCPIYQNQCVNTEKSHYSGTVNGTIHVVVGGAGSHLSNFSQVTPKWSLYRDFDFGFVKLTAFNH
SSLLFEYKKSRDGKVYDSFTVSRDYKNVLACVPDGCEATTLAS*
>CaPAP1a
MREAKLVFLAFLLVCSLLQKVWSHGKQPLSKVNIHKATLSLLDLAHIKASPSLLGLQGQIAEWV
TIEYSSPIPSTDDWIGVFSPANFSASTCPKENVRIFPPLLCSAPIKFQNASYLNPQYKITGKGFLKLQ
LINQRSDFSFALFSGGLSNPKLVAVSNKISFANPNAPVYPRLALGKSWNEMTVTWTSGYGISDAE
PVVEWGPKGEDHVHSPAGTLTFTRDSLCGAPAKSVGWRDPGYIHTSYLKELRPNIIYEYKIGHRL
NNGTYIWSKQYQFRAAPFPGQKSLQRVVIFGDMGKAEVDGSNEYNNFQQGSINTTQQLIQDLEN
IDMVFHIGDICYANGYISQWDQFTAQVEPIASVVPYMIASGNHERDWPGTGSFYGNMDSGGECG
VLAETMFYVPASNRAKFWYSIDYGMFRFCVADTEHDWREGTEQYKFIEHCLASVDRQKQPWLI
FVAHRVLGYSSCICYAEEGSFAEPMGRESLQKLWQKYKVDIAIYGHVHNYERTCPIYQNICTSEE
KHNYKGTLNGTIHIVAGGGGASLSTFTYLKTIWSIFKDYDYGFVKLTAFDHSTLLFEYKKSRDGK
VYDSFKISRDYRDILACAMDSCPSSTMAF*
>CaPAP18a
MRITWLTEDKHSSSFVEYGTLPGRYDSMAEGECTSYNYLLYSSRKIHHTVIGPLEHDTVYFYRCG
GQGPEFQLKTPPAQIPITFAVAGDLGQTGWTKSTLDHIDQCKYDVYLLPGDLSYADYGKMPFEE
SGSTSNLYYSFEVAGVHVIMLGSYADYDESSEQYTWLMADLSKVDRTRTPWLLVLFHVPWYNS
NRAQQGAGDDMMAVMEPLLYAASVDLVIAGNVHAYERSKRVYNGRLDRCGAVHITIGDGGNR
EGLAHRYINPQPNWSEFREASFGHGELKIVNSTHAFWSWHRNDYDESVKADDIWITSLVGSGCV
DHKRHELRSMLMTP
>CaPAP1b
MLLFSLLTFAIFQQVVSDVHQPLSKVAIHNTVFALDQGASIKATPNLLGLKGQNTEWVTLQYSNP
NPKIDDWIGVFSPANFSASTCPGENRLVNPPFLCSAPIKFQYANFSSHCYKNTGKGSLKLQLINQR

SDFSFALFTGGLTNPKLVAVSKKVSFVNPNAPVYPRLAQGKTWDEITVTWTSGYGISDAEPFVE
WGPKEGNLVKSPAGTLTFDRNTMCGAPARTVGWRDPGYIHTSFLKELWPNKEYTYKLGHRLVN
GTTIWSKKYEFKSSPYPGQNSVQRVVIFGDMGKAEADGSNEYNNFQPGSLNTTNQIIQDLKDIDV
VFHIGDLCYANGYLSQWDQFTAQIEPIASKVPYMTASGNHERDWPGSGSFYGNLDSGGECGVLA
QTMYSVDYGMFRFCIAHTELDWRKGTEQYNFIEKCLASVDRQKQPWLIFLAHRVLGYSSGDFY
VAEGSFEEPMGREDLQSLWQKYKVDIAMYGHVHNYERTCPIYQNICTDKEKHDYKGSLNGTIH
VVVGGGGAALADFAPINTTWSIFKDHDFGFVKLTAFNHSNLLLEYKKSSDGKVYDSFKISRDYR
DILACTADSCSSTSLAF
>CaPAP9
MILPATLFPLLLLLLILSSNLAQSKPSINVTPTTLTKSGDTVEIRWSGIESPSDLDWVGIYSPPTSSHD
NFIGYLFLSKSPTWQSGSGSLSLPLVNLRSNYSFRIFRWTRSEINPKRKDHDNNPLPQTRNLLGFS
QEVSFVSGRGPDQIHLSFSDQEDAMRVMYVTWDPKESYVKYGEREEKMEGLVVARAKRYERE
HMCDAPANQSVGWRDPGYIHDALITGLKKGKRYYYKVGNDNGGWSATHSFVSRNSDSNETIAF
LFGDMGTATPYNTFLRTQDESISTMKWILRDVEALGDKPSFVSHIGDISYARGYAWLWDHFFAQI
EPVATKVAYHVCIGNHEYDWPLQPWKPDWANYGKDGGGECGVPYSLRFNMPGNSSEPTGTVA
PATRNLYYSFDVGAVHFVYISTETNFLPGSNQYNFLKHDLESVDRSKTPFVVVQGHRPMYTTSN
EVRDAQLRGKMLEHLEPLLVNNNVTLALWGHVHRYEKFCPLNNYTCGNSVGRKAGDKEGYTV
HLVIGMAGQDWQPIWEPRPDHPNDPIFPQPTRSLYRAGEFGYIRLVATKQKLVISYVGNHDGQV
HDTMEILRSGEVVNGNGNGNGGIDSAKPEVQIEESTLSWYVQGGSVLVLGAFMGYILGFISRAR
KQPESRSGFTPVKTEET*
>CaPAP27a
MCLGDVNGFGEQPLSKIAIHKTILALHSSASITASPLLLGNKGEDNEWVTVEVESPEPTNDDWVG
VFSPANFNSSTCPPIPNGVGKLETPYICSAPIKYKYANHSNPNYKKTGKASLKFQLINQRADFSFA
LFSGGLSNPKLVTISNFIAFANPKAPVYPRLAQGKSWNEMTVTWTSGYDISEAVPFVEWGPKGR
KQIQSAAGTLTFNRNSMCGEPARTVGWRDPGFIHTGFLKELWPNMRYTYRLGHFLSDGSYVWS
KRYSFKASPYPGQNSLQRVIIFGDMGKAERDGSNEYADYQPGSLNTTDQLIKDLDNIDIVFHIGDL
PYANGYISQWDQFTAQVQQISSTVPYMIASGNHERSWPNSGSFYDTRDSDSEHDWREGSEQYKF
IEHCLSTVDRKQQPWLIFSAHRPLGYSSNSWYAMEGSFEEPMGRESLQGLWQKYKVDIAFYGHV
HNYERVCPIYQNQCVNKEKTHYSGTVNGTIHIVVGGGGSHLSDFTTAPPVWSLYKDRDYGFGKL
TAFNHSYLLFEYKKSSDGKVYDSFTISRDYRDVLACVHDGCEKTTLAS*
>CaPAP23a
MAVSKSHIPTTLDGPFEPVTRRFDPSLRRGSGDLPMTHPRLRMNVTANFPEQIALAISSPTSMWVS
WITGNAQIGVNVTPLNPASIGSEVWYGKESGNYTNIGKGDSLVYSQLYPFEGLLNYTSGIIHHVQ
LEGLEPGTRYYYKCGDSSVPAMSQENIFETFPTPSPKNYPTRIAVIGDLGLTSNSSTTIDHLIYNDP
SMILMIGDLTYANQYVTTGGKGVPCFSCAFPDAPIRETYQPRWDGWGR*
>CaPAP23b
MEALLYQYRVDIVFNGHVHAYERMNRVYNYTLDPCGPIYITVGDGGNIEKVDVDHADEPGKCP
SAGDNIPEFGGVCHSNYSSGPAKGNFCWNKQPEWSAFRESSFGHGILEVVNSTYALWTWHRNQ
DSYKENAVGDQIYIVRQPELCLKHSKV
>CaPAP7
MALCLNQHMLSPIIFVVSVLCLLANHSIAEELPRFKHHLKPQQQSLNFLVVGDWGRKGNYNQSF
VAHQMGIVGENLNIDFVISTGDNFYDDGLVGVDDPAFYESFVDIYTAPSLQKIWYSVLGNHDYR
GDVEAQLSPILRQKDSRWLCLRSFILDGGIVEFFFVDTTPFVEKYFTEPEDHTYDWRGVLPRESYV
AELLKNVDSALKQSNAKWKIVVGHHTIKTAGHHGNTQELEELLLPILKSNNVEAYINGHDHCLE
HIIDKESGTQFFTSGGGSKAWRGDIKPWNPEELKLYHDGQGFMSVQITNTIADFVFYDVFGKVLH
TWTISKEHKAAE*
>CaPAP17a

MAYLSGNSFFVYFLFTIIFGLGILHASAELQRFTHTPKSDGSLSFLVLGDWGRRGEYNQSEVAFQ
MGEVGEKLDIDFVISTGDNFYDDGLTSEHDPNFEESFSKIYTAKSLQKQWYSVLGNHDYRGDAE
AQLSPFLRQIDSKWLCLRSFIVDSELAEIFFVDTTPFVQEYFTESEEHNYDWKGIYPPKTYISNLLK
DLDMALRESTAKWKIVVGHHAIKSIGHHGDTQELISQLLPILQANNIDFYMNGHDHCLEHINDTK
SPIQFLTSGAGSKAWRGDIEETNRKDVNFFYDGQGFMSVQLTQTDAIILFYDVFGKVLHRIISSKE
LRSSM*

>CaPAP3
MGGLGTSSLSSLWLWLWIPTFIILSVSVSAELQRFDHPVNAADASLTFLVIGDWGRKGTYNQSQV
AFQMGRVADKLNIDFVVSTGDNFYDDGLTGIHDPAFQYSFSNIYTANSLQNQWYNVLGNHDYR
GDVKAQLNPILQNIDHRWFCQRSFIVDAEIAEFFFVDTTPFVDKYFFKPKDHKYDWRGVLPRDK
YLSNLLKDLETALKDSTAKWKIVVGHHPVRSIGHHGDTKELVTHLLPILEANNVDMYINGHDHC
LEHISSTSSQIQFLTSGGGSKAWKGDIHNNERDGVKFYYDGQGFMSVEIQQMNVKVAYYDIFGN
VLHVLNLSKGLQSVI*
>CaPAP8
MVLQLVFIGTIIQCLMYSSAVLQVFEHAPKSDGSLSFLVLGDWGRRGAYNQSQVAFQMGVIGEQ
LDIDFVISTGDNFYDNGLTGIDDTSFDYSFTKIYTAPSLQKQWYNVLGNHDYRGDVEAQLSPVLT
NLDNRWFCMRSYVVNAEFVEFFFVDTNPFVDSYFTEPGEHVYDWRGIGPRKQYISNLLKDVDLA
LKESNAKWKIVVGHHPIRSAAHHGDTKELVNQLLPILEANNIDLFINGHDHCLQHISSLKSGIEFM
TSGGGSKAWKGDVKWWNPEEMKLYYDGQGFMSLHVIQTQINVAFYDVFGNVLHNWNTSKQL
QSTF*
>CaPAP17b
MASPSSHLHLAAIILCIYFVVPIFAELQRFQHQPKHDGSVSYLVIGDWGRKGRYNQSRVATQMGK
VGEKLDIDFVVSTGDNFYTNGLKGVNDPAFLKSFSKIYTAKSLQKKWYTVLGNHDYRGNVPAQ
LSPLLRKIDNRWFCQRSFILNAGVAEFFFIDTTPFINDYYNFSEHFYDWRGVSPRKFYLNNLLKEF
ESALMKSNAIWKIVVGHHAIRSIGHHGDSPELVKYLIPILKANHVDVYMNGHDHCLQHISSIDGQ
LLYLTSGAGSKAWRGDIKESQFDVVKFFYDGQGFMSVQMTDIDANFAFYDVFGENIHHWNLSK
YSMHSSV*
>CaPAP28
MDSYNTTKQKNWKNSLLYLTFIVAILHLIHQSHFSRKLIIGNEKVHIKKNPQLPLRFRSDGTFKILQ
VADMHYGSGTITRCRDVLASEFEFCSDLNTTMFLKRIIQAETPDFIAFTGDNIFGSSAPDAAESLFE
AFGPAMESGLPWAAILGNHDQESTMNREELMSLISHMDYSVSQINPLADSLTDSAKIDGFGNYN
LRVYGAPGSILANSSVLNLFFLDSGDRAVYQGIRTYGWIKDSQLQWMRRVSHELQGQEQDPLHP
TPPALAFFHIPIPEVRQLFYKEIVGRFQEGVACSRVNSPVLQTFVSMGDVKAVFIGHDHKNDFCG
NLDGIWFCYGGGFGYHGYGKVGWPRRARIILAELQKGKMSWTSVQRIMTWKRLDDEKLSKIDE
QILWNR*
>CaPAP29
MGMSFMVLVVTVSWFWSISTTCVLAAKQAYISPQQENQKLRFDQNGEFKILQVADMHYANGK
NTLCLDVLPSQNISCSDLNTTAFIQRMILAEKPNLIVFTGDNIFGYDSSDSAKSMDAAFAPAVASNI
PWVAVLGNHDQEGSLSREGVMKHIVGMKNTLSKLNPPEVRIIDGFGNYNLEVGGVQGTEFENK
SVLNLYFLDSGDYSKVPTIPGYDWIKPSQQLWFKRTSAELRDGNNGISSASVNSGFFTTLVEAGD
VKAVFTGHDHINDFCGKLMDIQLCYAGGFGYHAYGKAGWSRRARVVVASLEKTDKGSWGGV
KSIKSWKRLDDQQLTGIDGEVLWSKSFRGNPPWKFYTLRKLIVSMKIESVEDNKR
>CaPAP16
MVMVSWFWLIPATSSSSLPQQQENQKLRFDQNGEFKILQVADMHYADGKNTLCLDVLPSQNAS
CTDLNTTAFIQRMILAEKPNLIVFTGDNIFGFDSSDSAKSMDAAFAPAIASNIPWVAVLGNHDQEG

SLSREGVMKYIVGMKNTLSKLNPPEVHIIDGFGNYNLEVGGVQGTVFENKSVLNLYFLDSGDYS
KVPAIFGYDWIKPSQQLWFERMSAKLRKAYIKGPVPQKEAAPGLAYFHIPLPEYASFDSSNFTGV
KMEPDGISSASVNSGFFTTLVEAGDVKAVFTGHDHLNDFCGKLMDIQLCYAGGFGYHAYGKAG
WSRRARVVVASLEKTDKGSWGDVKSIKSWKRLDDQHLTGIDGEVLWSKSFRDYEMLDLKAQI
VLKPEEKNRTTIAHKFMNWEEKFCQVDKRFNDLWLMFIMY*
